# Hearing loss prevalence and years lived with disability, 1990–2019: findings from the Global Burden of Disease Study 2019

**DOI:** 10.1016/S0140-6736(21)00516-X

**Published:** 2021-03-13

**Authors:** GBD 2019 Hearing Loss Collaborators, Lydia M Haile, Kaloyan Kamenov, Paul Svitil Briant, Aislyn U Orji, Jaimie D Steinmetz, Amir Abdoli, Mohammad Abdollahi, Eman Abu-Gharbieh, Ashkan Afshin, Haroon Ahmed, Tarik Ahmed Rashid, Yonas Akalu, Fares Alahdab, Fahad Mashhour Alanezi, Turki M Alanzi, Hanadi Al Hamad, Liaqat Ali, Vahid Alipour, Rajaa M Al-Raddadi, Hubert Amu, Jalal Arabloo, Morteza Arab-Zozani, Judie Arulappan, Charlie Ashbaugh, Desta Debalkie Atnafu, Zaheer-Ud-Din Babar, Atif Amin Baig, Palash Chandra Banik, Till Winfried Bärnighausen, Amadou Barrow, Rose G Bender, Akshaya Srikanth Bhagavathula, Nikha Bhardwaj, Pankaj Bhardwaj, Sadia Bibi, Ali Bijani, Katrin Burkart, Christopher R Cederroth, Jaykaran Charan, Sonali Gajanan Choudhari, Dinh-Toi Chu, Rosa A S Couto, Amare Belachew Dagnew, Baye Dagnew, Saad M A Dahlawi, Xiaochen Dai, Lalit Dandona, Rakhi Dandona, Assefa Desalew, Deepak Dhamnetiya, Mandira Lamichhane Dhimal, Meghnath Dhimal, Kerrie E Doyle, Bruce B Duncan, Michael Ekholuenetale, Irina Filip, Florian Fischer, Richard Charles Franklin, Abhay Motiramji Gaidhane, Shilpa Gaidhane, Silvano Gallus, Farhad Ghamari, Ahmad Ghashghaee, Ghozali Ghozali, Syed Amir Gilani, Ionela-Roxana Glavan, Mahaveer Golechha, Bárbara Niegia Garcia Goulart, Veer Bala Gupta, Vivek Kumar Gupta, Samer Hamidi, Billy Randall Hammond, Simon I Hay, Khezar Hayat, Golnaz Heidari, Howard J Hoffman, Kathleen Pillsbury Hopf, Mehdi Hosseinzadeh, Mowafa Househ, Rabia Hussain, Bing-Fang Hwang, Ivo Iavicoli, Segun Emmanuel Ibitoye, Olayinka Stephen Ilesanmi, Seyed Sina Naghibi Irvani, Sheikh Mohammed Shariful Islam, Masao Iwagami, Louis Jacob, Sathish Kumar Jayapal, Ravi Prakash Jha, Jost B Jonas, Rohollah Kalhor, Nawzad Kameran Al-Salihi, Himal Kandel, Ayele Semachew Kasa, Gbenga A Kayode, Rovshan Khalilov, Ejaz Ahmad Khan, Mahalaqua Nazli Khatib, Soewarta Kosen, Ai Koyanagi, G Anil Kumar, Iván Landires, Savita Lasrado, Stephen S Lim, Xuefeng Liu, Stany W Lobo, Alessandra Lugo, Alaa Makki, Walter Mendoza, Amanual Getnet Mersha, Kebadnew Mulatu Mihretie, Ted R Miller, Sanjeev Misra, Teroj Abdulrahman Mohamed, Mokhtar Mohammadi, Abdollah Mohammadian-Hafshejani, Arif Mohammed, Ali H Mokdad, Mohammad Ali Moni, Sandhya Neupane Kandel, Huong Lan Thi Nguyen, Molly R Nixon, Jean Jacques Noubiap, Virginia Nuñez-Samudio, Bogdan Oancea, Victor Maduabuchi Oguoma, Andrew T Olagunju, Bolajoko Olubukunola Olusanya, Jacob Olusegun Olusanya, Hans Orru, Mayowa O Owolabi, Jagadish Rao Padubidri, Keyvan Pakshir, Shahina Pardhan, Fatemeh Pashazadeh Kan, Maja Pasovic, Shrikant Pawar, Hai Quang Pham, Marina Pinheiro, Akram Pourshams, Navid Rabiee, Mohammad Rabiee, Amir Radfar, Fakher Rahim, Vafa Rahimi-Movaghar, Mohammad Hifz Ur Rahman, Mosiur Rahman, Amir Masoud Rahmani, Juwel Rana, Chythra R Rao, Sowmya J Rao, Vahid Rashedi, David Laith Rawaf, Salman Rawaf, Andre M N Renzaho, Aziz Rezapour, Rezaul Karim Ripon, Voilet Rodrigues, Neeti Rustagi, Umar Saeed, Amirhossein Sahebkar, Abdallah M Samy, Milena M Santric-Milicevic, Brijesh Sathian, Maheswar Satpathy, Monika Sawhney, Winfried Schlee, Maria Inês Schmidt, Allen Seylani, Masood Ali Shaikh, Mohammed Shannawaz, Wondimeneh Shibabaw Shiferaw, Soraya Siabani, Anjali Singal, Jasvinder A Singh, Jitendra Kumar Singh, Deepika Singhal, Valentin Yurievich Skryabin, Anna Aleksandrovna Skryabina, Houman Sotoudeh, Emma Elizabeth Spurlock, Biruk Wogayehu Taddele, Animut Tagele Tamiru, Md Ismail Tareque, Rekha Thapar, Marcos Roberto Tovani-Palone, Bach Xuan Tran, Saif Ullah, Sahel Valadan Tahbaz, Francesco S Violante, Vasily Vlassov, Bay Vo, Avina Vongpradith, Giang Thu Vu, Jingkai Wei, Ali Yadollahpour, Seyed Hossein Yahyazadeh Jabbari, Yigizie Yeshaw, Vahit Yigit, Birhanu Wubale Yirdaw, Naohiro Yonemoto, Chuanhua Yu, Ismaeel Yunusa, Mohammad Zamani, Mikhail Sergeevich Zastrozhin, Anasthasia Zastrozhina, Zhi-Jiang Zhang, Jeff T Zhao, Christopher J L Murray, Adrian C Davis, Theo Vos, Shelly Chadha

## Abstract

**Background:**

Hearing loss affects access to spoken language, which can affect cognition and development, and can negatively affect social wellbeing. We present updated estimates from the Global Burden of Disease (GBD) study on the prevalence of hearing loss in 2019, as well as the condition's associated disability.

**Methods:**

We did systematic reviews of population-representative surveys on hearing loss prevalence from 1990 to 2019. We fitted nested meta-regression models for severity-specific prevalence, accounting for hearing aid coverage, cause, and the presence of tinnitus. We also forecasted the prevalence of hearing loss until 2050.

**Findings:**

An estimated 1·57 billion (95% uncertainty interval 1·51–1·64) people globally had hearing loss in 2019, accounting for one in five people (20·3% [19·5–21·1]). Of these, 403·3 million (357·3–449·5) people had hearing loss that was moderate or higher in severity after adjusting for hearing aid use, and 430·4 million (381·7–479·6) without adjustment. The largest number of people with moderate-to-complete hearing loss resided in the Western Pacific region (127·1 million people [112·3–142·6]). Of all people with a hearing impairment, 62·1% (60·2–63·9) were older than 50 years. The Healthcare Access and Quality (HAQ) Index explained 65·8% of the variation in national age-standardised rates of years lived with disability, because countries with a low HAQ Index had higher rates of years lived with disability. By 2050, a projected 2·45 billion (2·35–2·56) people will have hearing loss, a 56·1% (47·3–65·2) increase from 2019, despite stable age-standardised prevalence.

**Interpretation:**

As populations age, the number of people with hearing loss will increase. Interventions such as childhood screening, hearing aids, effective management of otitis media and meningitis, and cochlear implants have the potential to ameliorate this burden. Because the burden of moderate-to-complete hearing loss is concentrated in countries with low health-care quality and access, stronger health-care provision mechanisms are needed to reduce the burden of unaddressed hearing loss in these settings.

**Funding:**

Bill & Melinda Gates Foundation and WHO.

## Introduction

Hearing loss is common and can negatively affect multiple aspects of an individual's life when unaddressed or when individuals' communication needs are unsupported. Auditory deprivation can detract from quality of life and access to spoken communication, which can impede development of child spoken language^1^ and contribute to the risk of dementia^2^ and cognitive decline in older ages.^3^ Deafness in early life when unaddressed is associated with poor literacy outcomes and reduced employment opportunities in later life. The emotional effects of hearing loss can include loneliness, isolation, depression, and anxiety.^4^, ^5^, ^6^ In many countries, children with hearing loss rarely receive schooling^7^ and adults have a much higher unemployment rate compared with their hearing peers.^8^

The effect of hearing loss is broadly recognised by several stakeholders, including researchers, clinicians, policy makers, and people living with the condition.^9^ Announced during the celebration of World Hearing Day 2019, the *Lancet* Commission on Global Hearing Loss aims to examine how to reduce the burden of hearing loss, convening expert working groups on prevention, policy, technology, and protection.^10^ Efforts from *The Lancet* are complemented by plans from WHO to release the first-ever world report on hearing. The report seeks to utilise best available evidence to present a consistent narrative on hearing loss and hearing health care, call attention to priorities and best practices for hearing health care, and describe trends in the global distribution of hearing loss.^11^ Initiatives from researchers and other key decision makers underscore the relevance of hearing loss within the global health landscape and the condition's effects.

Researchers have called for urgent multi-disciplinary action on hearing health care, including comprehensive screening programmes, increased access to hearing devices, and noise-reduction strategies.^12^ Despite the effects of hearing loss on spoken communication and wellbeing, literature on the prevalence of hearing loss is relatively sparse, making the understanding of who is most affected and where resources should be allocated difficult.

**Research in context**

**Evidence before this study**

The prevalence of hearing loss is estimated annually through the Global Burden of Disease (GBD) study, a systematic overview of the prevalence of 369 diseases and injuries. The latest GBD study on hearing loss was based on estimates from 10 years ago. Since then, we have obtained new sources of data through systematic reviews of population-representative studies, collaborator input, and additional targeted searches. Sources up to Dec 31, 2008, were obtained from a previously published systematic review. In GBD 2013, we did a systematic review of sources published 2008–13 onward using the following PubMed search string: “(hearing loss[Title/Abstract] OR deafness[Title/Abstract] OR hearing loss[Title/Abstract]) AND (prevalence[Title/Abstract]) AND (“2008”[PDAT] : “3000”[PDAT]) AND (cross sectional OR survey)”.

In GBD 2016, we did an update systematic review of sources published between Nov 26, 2008, and Nov 30, 2016, using the following PubMed search string: “(hearing loss[Title/Abstract] OR deafness[Title/Abstract] OR hearing loss[Title/Abstract] OR audiometry[Title/Abstract]) AND (prevalence[Title/Abstract]) AND (“2008/11/26”[PDAT]: “3000”[PDAT]) AND (cross sectional OR survey)”, without any language restrictions. Sources that included self-reported data, did not report hearing loss in the less severe ear, were not population representative, or did not report a definitive decibel-level threshold for reported hearing loss were excluded. Sources that reported bilateral hearing loss according to the GBD reference definition were included.

**Added value of this study**

The GBD hearing loss paper published in 2010 was informed by 42 studies in 29 countries. Our analysis provides an update that is informed by 215 survey sources from 77 countries. Method changes for the GBD 2019 iteration include new methods of adjusting data using non-standard hearing thresholds and splitting data with gender unspecified by sex. These GBD estimates are accompanied by forecasts of hearing loss prevalence until 2050, providing a comprehensive picture of current and future disease trends. We found that 1·57 billion people (95% uncertainty interval 1·51–1·64) had hearing loss in 2019, contributing to 43·45 million (29·68–61·80) years lived with disability as the third largest cause of disability in GBD. The global number of individuals with hearing loss is also projected to increase by 56·1% (47·3–65·2) in the next 30 years despite stable age-standardised prevalence.

**Implications of all the available evidence**

This study accompanies the 2021 WHO World Hearing Report, which describes the burden of hearing loss using the GBD 2019 results and standards for ear and hearing care, and sets standards for policy and prevention. Our analysis provides evidence that hearing loss is largely caused by ageing, will increase in prevalence in coming years, and is more prevalent in countries with low health-care access. Cost-effective, preventive, and treatment interventions for hearing loss are available, including school-based screening programmes, hearing devices (eg, hearing aids or cochlear implants), and clinical management of ear disease. Health-care services must be prepared for large projected increases in burden, particularly in low-income and middle-income countries where ear specialists and audiologists are scarce.

The Global Burden of Diseases, Injuries, and Risk Factors (GBD) Study is a comprehensive attempt to quantify the contribution of hearing loss relative to other causes of ill health. The study provides annual data on the prevalence and associated levels of disability for 369 diseases and injuries, as well as 87 risk factors. Estimates are updated annually, most recently for GBD 2019—the most recent GBD paper on hearing loss was based on GBD 2010 estimates.^13^ In this Article, we aimed to estimate the prevalence, severity, and distribution of global hearing loss and added forecasts of future disease prevalence through 2050.

## Methods

### Overview

The 2019 GBD produced estimates for 369 diseases and injuries; high-level methods have been described in detail elsewhere.^14^ Data for the prevalence and incidence of non-fatal conditions were pooled in DisMod-MR 2.1, a Bayesian meta-regression tool (described elsewhere).^15^

In addition to prevalence and incidence, GBD uses the years lived with disability (YLDs) metric to compare the morbidity associated with different non-fatal conditions. YLDs are calculated by multiplying the prevalence of a condition with its associated disability weight, reflecting the severity of that disease relative to all other health states. Disability weights range from 0 (meaning perfect health) to 1, which is equivalent to death.

This study complies with the Guidelines for Accurate and Transparent Health Estimates Reporting (GATHER) recommendations (appendix p 16).^16^

### Definition of hearing loss

GBD defines hearing loss as the quietest sound an individual can hear in their better ear, taken as the pure-tone average of audiometric thresholds of 0·5 kHz, 1 kHz, 2 kHz, and 4 kHz. Hearing loss is reported in the GBD by seven mutually exclusive severity categories (table 1).^17^ Two health states were assigned to each severity category on the basis of the presence of tinnitus, a ringing or buzzing in the ears that is associated with hearing loss.^18^ Moderate-to-complete hearing loss consisted of all hearing loss greater than 35 dB and is most often associated with reduced functional outcomes in individuals.^11^ All hearing loss is defined as any hearing loss above 20 dB, ranging from mild to complete in severity.

**Table 1:** Hearing loss health states, lay descriptions, thresholds on pure tone audiometry, and disability weights

	**Lay description**	**Range**	**Disability weight (95% UI)**
Normal	Normal hearing	0–19 dB	0
Mild	Has great difficulty hearing and understanding another person talking in a noisy place (eg, on an urban street)	20–34 dB	0·010 (0·004–0·019)
Mild with ringing	Has great difficulty hearing and understanding another person talking in a noisy place (eg, on an urban street), and sometimes has annoying ringing in the ears	20–34 dB	0·021 (0·012–0·036)
Moderate	Is unable to hear and understand another person talking in a noisy place (eg, on an urban street), and has difficulty hearing another person talking even in a quiet place or on the telephone	35–49 dB	0·027 (0·015–0·042)
Moderate with ringing	Is unable to hear and understand another person talking in a noisy place (eg, on an urban street), and has difficulty hearing another person talking even in a quiet place or on the telephone, and has annoying ringing in the ears for more than 5 min at a time, almost every day	35–49 dB	0·074 (0·048–0·107)
Moderately severe	No lay description available	50–64 dB	0·092 (0·064–0·129)
Moderately severe with ringing	No lay description available	50–64 dB	0·167 (0·114–0·231)
Severe	Is unable to hear and understand another person talking, even in a quiet place, and unable to take part in a telephone conversation; difficulties with communicating and relating to others sometimes cause emotional effects (eg, worry or depression)	65–79 dB	0·158 (0·104–0·227)
Severe with ringing	Is unable to hear and understand another person talking, even in a quiet place, and unable to take part in a telephone conversation, and has annoying ringing in the ears for more than 5 min at a time, almost every day; difficulties with communicating and relating to others sometimes cause emotional effects (eg, worry or depression)	65–79 dB	0·261 (0·174–0·361)
Profound	Is unable to hear and understand another person talking, even in a quiet place, is unable to take part in a telephone conversation, and has great difficulty hearing anything in any other situation; difficulties with communicating and relating to others often cause worry, depression, and loneliness	80–94 dB	0·204 (0·134–0·288)
Profound with ringing	Is unable to hear and understand another person talking, even in a quiet place, is unable to take part in a telephone conversation, has great difficulty hearing anything in any other situation, and has annoying ringing in the ears for more than 5 min at a time, several times a day; difficulties with communicating and relating to others often cause worry, depression, or loneliness	80–94 dB	0·277 (0·182–0·388)
Complete	Cannot hear at all in any situation, including even the loudest sounds, and cannot communicate verbally or use a telephone; difficulties with communicating and relating to others often cause worry, depression, or loneliness	95+ dB	0·215 (0·143–0·307)
Complete with ringing	Cannot hear at all in any situation, including even the loudest sounds, and cannot communicate verbally or use a telephone, and has very annoying ringing in the ears for more than half of the day; difficulties with communicating and relating to others often cause worry, depression, or loneliness	95+ dB	0·316 (0·211–0·436)

Lay descriptions as listed were used in GBD disability weight surveys (appendix p 3). GBD=Global Burden of Disease. UI=uncertainty interval.

This manuscript was produced as part of the GBD Collaborator Network and in accordance with the GBD Protocol.

### Data processing

We used input data obtained via systematic reviews of epidemiological, population-representative surveys. Sources up to Nov 26, 2008, were obtained via a published systematic review, ^19^ and two additional systematic reviews done in 2013 and 2016 identified newer sources (appendix p 3). Sources were excluded that recorded self-reported hearing loss, were not population-representative, did not report bilateral (better ear) hearing loss, or did not use pure-tone audiometry to quantify hearing loss.

For data sources that reported prevalence by age and sex separately, we applied the proportion of female and male prevalence to age-specific data. We then ran a meta-regression on the log ratio of female and male prevalence in MR-BRT (meta-regression—Bayesian, regularised, trimmed; methodology has been described elsewhere^14^) and applied the model results to sex-split data reported for both sexes combined (appendix p 3).

Data sources that reported hearing loss by severity categories that differed from the GBD categories were adjusted using data from US National Health and Nutrition Examination surveys (NHANES).^20^ NHANES reported individual-level data on the exact decibel at which hearing loss was experienced. Adjustment factors were derived by running a meta-regression on the logit difference between the prevalence of the alternative and reference categories (appendix p 3). Data sources that reported prevalence in age groups larger than 20 years were split into 5-year age groups by applying the global age pattern of the GBD 2017 model.

### Modelling strategy

To model hearing loss, we first ran three DisMod-MR 2.1 models to estimate the prevalence of no hearing loss (0–19 dB), mild hearing loss (20–34 dB), and moderate-to-complete hearing loss (≥35 dB). Socio-demographic Index (a summary measure of fertility, education, and gross domestic product)^14^ was used as a covariate in each model. These models were rescaled so that prevalence rates summed to one for each age, year, sex, and location. Second, we ran five DisMod models for the more severe levels of hearing loss and rescaled these to the prevalence of at least 35 dB hearing loss. Third, we ran one DisMod model for hearing aid coverage (defined as the proportion of individuals who use a hearing aid) and another for the proportion of hearing loss attributable to age-related or other factors. Uncertainty of final estimates was derived from the uncertainty in input data, data manipulations, and predictive covariates. We estimated uncertainty by running each model until convergence, then taking the 975th and 25th ordered draw from 1000 posterior model runs as 95% uncertainty intervals (UIs) for each point estimate.

Fourth, severity-specific prevalence of hearing loss was adjusted to account for hearing aid usage (appendix p 4). We calculated hearing aid coverage rates for each severity, age, sex, and location, using survey data and regression methods (appendix p 4). The identified proportion of individuals at each severity level who used a hearing aid were then shifted to the category directly below. Adjusted hearing loss refers to estimates that were adjusted downwards to account for hearing aid usage. By contrast, unadjusted hearing loss refers to estimates that were not adjusted downwards for hearing aid usage.

Fifth, we estimated the prevalence of hearing loss due to underlying causes—ie, chronic otitis media, congenital birth defects, meningitis, and age-related or other factors (appendix p 4). From birth to age 20 years, we scaled hearing loss prevalence due to congenital birth defects, otitis media, age-related and other factors, and meningitis to the total prevalence of each severity level. Above age 20 years, the difference between total hearing loss prevalence and hearing loss prevalence due to otitis media, meningitis, and congenital birth defects was assigned to age-related and other hearing loss.

Sixth, we estimated the prevalence of hearing loss with and without tinnitus (appendix p 5). We estimated the proportion of individuals with tinnitus using NHANES data, which reported the proportion of respondents with hearing impairment who experienced ringing, roaring, or buzzing. In the absence of cause-specific estimates of tinnitus, we assumed the same distribution across all causes. We applied proportions from NHANES data to our estimates to split hearing loss prevalence into prevalence with and without tinnitus.

### Healthcare Access and Quality Indexand WHO regions

Results are presented by Healthcare Access and Quality (HAQ) Index as well as by WHO region. HAQ Index is a summary measure of the quality of national health systems that ranges from 0 to 100 and is based on analysis of avoidable deaths for 32 causes (methods described elsewhere).^21^

WHO member states are classified into six distinct regions: the Western Pacific region, South-East Asia region, European region, Region of the Americas, African region, and Eastern Mediterranean region (appendix p 6). Regional results in this paper are restricted to WHO member states and were produced in collaboration with WHO for the 2021 World Hearing Report.^11^

### Forecasting global and regional hearing loss prevalence

Forecasts of hearing loss prevalence were generated using age-specific prevalence rates for the years 1990, 1995, 2000, 2005, 2010, 2015, and 2019 as input data into a regression, with year, WHO region, and age as predictors. We included an interaction term between region and year and a cubic spline on age. Sex-specific regression models were run 1000 times, and the resulting coefficients were used to predict rates in the years 2030, 2040, and 2050. Predicted rates were multiplied by forecasted population for each WHO location to obtain case numbers, and then aggregated to obtain global estimates.^22^

## Results

Source counts were calculated by summing all surveys that were considered for incorporation into the model, including those that were eventually excluded from the analysis (because of poor survey methodology, etc). We counted a survey reporting data from multiple countries as multiple data sources. In total, input data consisted of 215 survey sources in 77 countries (surveys were only counted once in the total summation, even if they were used in multiple aspects of hearing loss estimation). Modelling of hearing loss prevalence considered 113 survey sources in 54 countries. Modelling of hearing aid coverage considered 100 survey sources in 44 countries. Other data came from ten data sources in two countries (data coverage and age-standardised coverage rates are shown in the appendix [pp 7, 9]).

An estimated 1·57 billion (95% UI 1·51–1·64) people had hearing loss in 2019, accounting for 20·3% (19·5–21·1) of the global population. Of these, 1·17 billion (1·12–1·22) people (74·3% [71·8–76·8]) had mild hearing loss. 12·65 million (10·34–15·48) individuals had complete hearing loss. Without adjustment for hearing aid use, 430·4 million (381·7–479·6) people globally had moderate-to-complete hearing loss in the better-hearing ear.

Globally, the number of people with moderate-to-complete hearing loss increased from 225·3 million (95% UI 197·6–250·9) in 1990, to 403·3 million (357·3–449·5) in 2019, a 79·1% (73·8–84·1) increase.

Age-standardised prevalence of moderate-to-complete hearing loss remained constant over the same period (age-standardised prevalence for all hearing loss is presented in the appendix [p 9]), ranging from 5·8% (95% UI 5·2–6·5) in the South-East Asia region to 3·5% (3·1–3·9) in the European region. The largest number of people with moderate-to-complete hearing loss resided in the Western Pacific region (127·1 million [112·3–142·6]), the South-East Asia region (103·4 million [90·9–115·4]), and the region of the Americas (58·8 million [51·7–66·2]; table 2).

**Table 2:** Global and regional prevalent cases (in millions) and age-standardised prevalence by severity of hearing loss

	**Age-standardised rate (95% UI)**	**Cases in millions (95% UI)**
**Hearing loss, ≥20dB**		
Global	19·3% (18·5–20·0)	1571·3 (1511·9–1635·5)
African region	18·9% (18·2–19·6)	136·7 (130·1–143·4)
Eastern Mediterranean region	14·9% (14·3–15·6)	78·8 (75·3–82·4)
European region	14·3% (13·7–15·0)	194·5 (185·4–204·0)
Region of the Americas	18·0% (17·3–18·9)	215·5 (205·9–225·6)
South-East Asia region	21·2% (20·4–22·0)	400·0 (385·2–415·8)
Western Pacific region	21·5% (20·7–22·3)	540·2 (519·6–562·0)
**Moderate-to-complete hearing loss, ≥35 dB**		
Global	5·1% (4·5–5·6)	403·3 (357·3–449·5)
African region	5·4% (4·8–6·1)	38·7 (33·1–44·5)
Eastern Mediterranean region	4·7% (4·1–5·2)	21·3 (18·6–23·9)
European region	3·5% (3·1–3·9)	52·8 (46·2–59·6)
Region of the Americas	4·7% (4·2–5·3)	58·8 (51·7–66·2)
South-East Asia region	5·8% (5·2–6·5)	103·4 (90·9–115·4)
Western Pacific region	5·1% (4·5–5·7)	127·1 (112·3–142·6)
**Mild hearing loss, 20–34 dB**		
Global	14·2% (13·6–14·8)	1167·9 (1116·4–1219·6)
African region	13·4% (12·8–14·0)	98·0 (93·4–102·5)
Eastern Mediterranean region	10·3% (9·8–10·7)	57·6 (54·8–60·4)
European region	10·8% (10·3–11·4)	141·7 (134·8–148·9)
Region of the Americas	13·3% (12·6–14·0)	156·7 (148·0–165·1)
South-East Asia region	15·3% (14·7–16·0)	296·6 (284·0–309·3)
Western Pacific region	16·4% (15·7–17·1)	413·2 (393·3–431·4)
**Moderate hearing loss, 35–49 dB**		
Global	3·3% (2·9–3·8)	268·8 (235·5–302·4)
African region	3·2% (2·8–3·6)	22·5 (19·0–26·3)
Eastern Mediterranean region	3·0% (2·6–3·3)	13·6 (11·8–15·4)
European region	2·4% (2·1–2·7)	36·2 (31·4–41·3)
Region of the Americas	3·3% (2·9–3·7)	41·1 (35·9–46·3)
South-East Asia region	3·8% (3·4–4·3)	69·1 (60·1–78·0)
Western Pacific region	3·3% (2·9–3·7)	85·5 (75·0–96·9)
**Moderately severe hearing loss, 50–64 dB**		
Global	1·1% (0·9–1·3)	85·0 (71·6–101·3)
African region	1·4% (1·2–1·6)	8·8 (7·3–10·6)
Eastern Mediterranean region	1·1% (0·9–1·3)	4·7 (3·9–5·5)
European region	0·7% (0·6–0·9)	11·8 (9·6–14·3)
Region of the Americas	0·9% (0·8–1·1)	11·7 (9·7–14·1)
South-East Asia region	1·3% (1·1–1·6)	21·9 (18·1–26·3)
Western Pacific region	1·0% (0·9–1·2)	25·8 (21·5–31·2)
**Severe hearing loss, 65–79 dB**		
Global	0·3% (0·2–0·3)	19·6 (15·8–24·1)
African region	0·5% (0·4–0·6)	3·7 (2·9–4·6)
Eastern Mediterranean region	0·3% (0·2–0·3)	1·2 (1·0–1·5)
European region	0·1% (0·1–0·2)	1·8 (1·4–2·3)
Region of the Americas	0·2% (0·2–0·2)	2·3 (1·8–2·9)
South-East Asia region	0·3% (0·2–0·3)	4·8 (3·8–6·0)
Western Pacific region	0·2% (0·2–0·3)	5·7 (4·5–7·1)
**Profound hearing loss, 80–94 dB**		
Global	0·2% (0·2–0·3)	17·3 (13·7–21·5)
African region	0·2% (0·2–0·3)	2·0 (1·5–2·7)
Eastern Mediterranean region	0·2% (0·1–0·2)	1·1 (0·8–1·4)
European region	0·1% (0·1–0·2)	1·6 (1·3–2·0)
Region of the Americas	0·2% (0·1–0·2)	2·1 (1·7–2·7)
South-East Asia region	0·2% (0·2–0·3)	4·8 (3·7–6·1)
Western Pacific region	0·3% (0·2–0·3)	5·5 (4·4–6·9)
**Complete hearing loss, ≥95 dB**		
Global	0·2% (0·1–0·2)	12·6 (10·3–15·5)
African region	0·2% (0·2–0·2)	1·7 (1·3–2·1)
Eastern Mediterranean region	0·1% (<0·1–0·1)	0·7 (0·5–0·8)
European region	0·1% (<0·1–0·1)	1·4 (1·1–1·7)
Region of the Americas	0·1% (0·1–0·2)	1·6 (1·3–1·9)
South-East Asia region	0·2% (0·1–0·2)	2·8 (2·2–3·5)
Western Pacific region	0·2% (0·2–0·2)	4·5 (3·7–5·5)

UI=uncertainty interval.

Between 1990 and 2019, the global number of YLDs attributable to hearing loss increased by 73·6% (95% UI 67·1–79·2), from 25·02 million (16·96–35·34) to 43·45 million (29·68–61·80). Of the YLDs attributed to hearing loss in 2019, 65·2% (52·5–76·6) were caused by moderate-to-complete cases and 34·8% (23·4–47·5) were caused by mild cases. In 2019, 7·00 million (4·76–10·06) YLDs were attributable to occupational noise exposure (appendix p 5).

**Figure 1:**
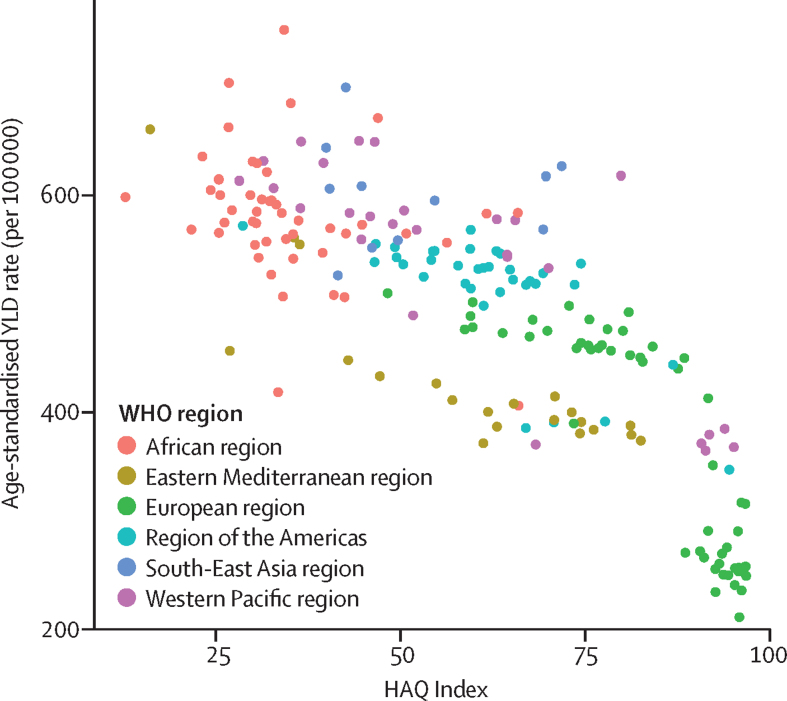
Age-standardised YLD rates for all hearing loss (per 100 000 population) by HAQ Index HAQ=Healthcare Access and Quality. YLD=years lived with disability.

The burden of all hearing loss is greatest at lowest levels of the HAQ Index, with a difference of more than 3 times in YLD rates between countries with the lowest and highest HAQ Index values (figure 1). A linear regression analysis of age-standardised national YLD rates of hearing loss and HAQ Index found that HAQ Index explained 65·8% of the variation in YLD rates (adjusted *r*^2^ 0·66).

**Figure 2:**
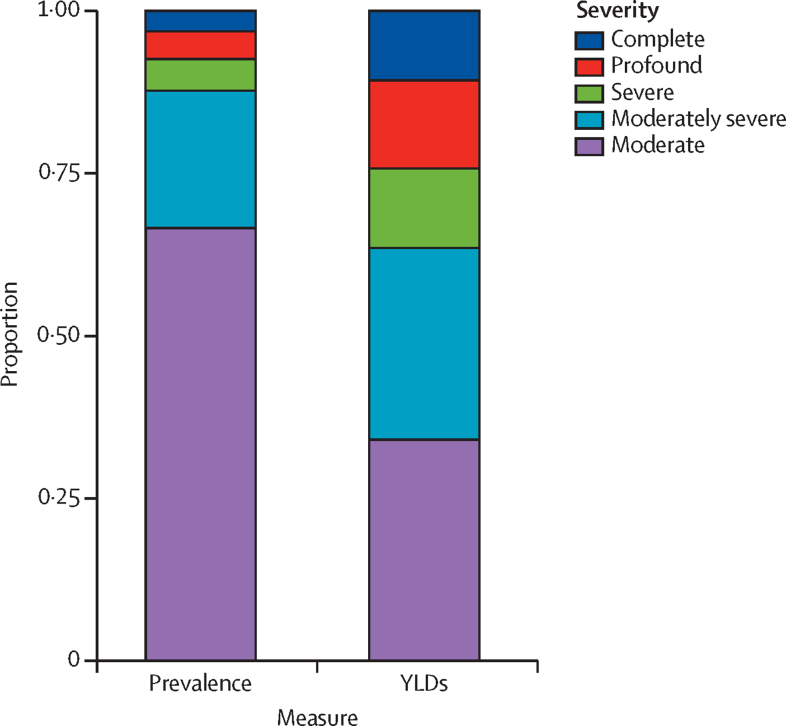
Proportion of individuals with moderate-to-complete hearing loss by measure and severity YLD=years lived with disability.

Although 66·6% (95% UI 62·9–69·9) of moderate-to-complete hearing loss cases were moderate in severity (35–49 dB), this severity accounted for 34·0% (26·2–42·6) of disability attributed to moderate-to-complete hearing loss (figure 2). Of all YLDs attributable to moderate-to-complete cases, 34·0% (26·2–42·6) were attributable to moderate cases, followed by moderately severe (29·6% [25·6–33·8]), profound (13·6% [10·5–17·2]), severe (12·1% [9·7–14·8]), and complete cases (10·7% [8·3–13·6]).

**Figure 3:**
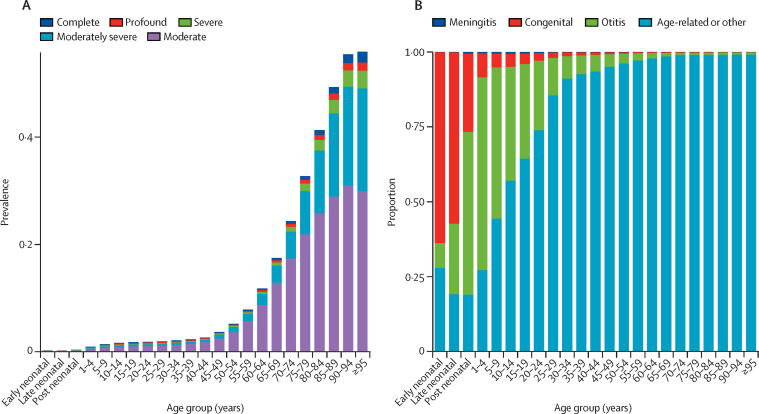
Prevalence of hearing loss 35 dB or greater by age, severity, and cause Prevalence of hearing loss 35 dB or greater by age and severity (A) and proportion of individuals with hearing loss by age and cause for all severities (B).

Across the age spectrum, hearing loss was most severe in those approximately younger than 5 years and those older than 70 years, meaning that the proportion of cases that were moderately severe, severe, profound, or complete was much larger in these age groups (appendix p 10). Hearing loss was also more prevalent at older ages, most notably after age 50 years (figure 3). In 2019, 62·1% (95% UI 60·2–63·9) of all people with hearing impairment were older than 50 years, and 4·4% (3·9–5·0) were younger than 15 years (69·7 million individuals [59·9–78·8]). Compared with other disease categories in the GBD, age-related and other hearing loss was the third largest cause of global YLDs in 2019, and the leading cause of global YLDs for individuals older than 70 years. Full rankings are available at GBD Compare.

The cause of hearing loss changed from infections and congenital birth defects to other factors over age (figure 3). Under age 5 years, hearing loss was mostly attributable to otitis media (63·7% [95% UI 56·9–70·2]). By comparison, 96·2% (94·5–97·6) of hearing loss was attributable to age-related and other factors for adults aged 50–54 years.

Between 1990 and 2019, the crude prevalence rate of all hearing loss increased by 27·8% (95% UI 26·6–29·0), from 15·9% (15·3–16·6) in 1990 to 20·3% (19·5–21·1) in 2019. By contrast, the global age-standardised prevalence rate of all hearing loss remained stable, changing from 19·1% (18·4–19·9) in 1990 to 19·3% (18·5–20·0) in 2019. The increase in hearing loss cases while age-standardised reates remained stable indicates that increases in prevalent cases are driven by population growth and ageing.

**Figure 4:**
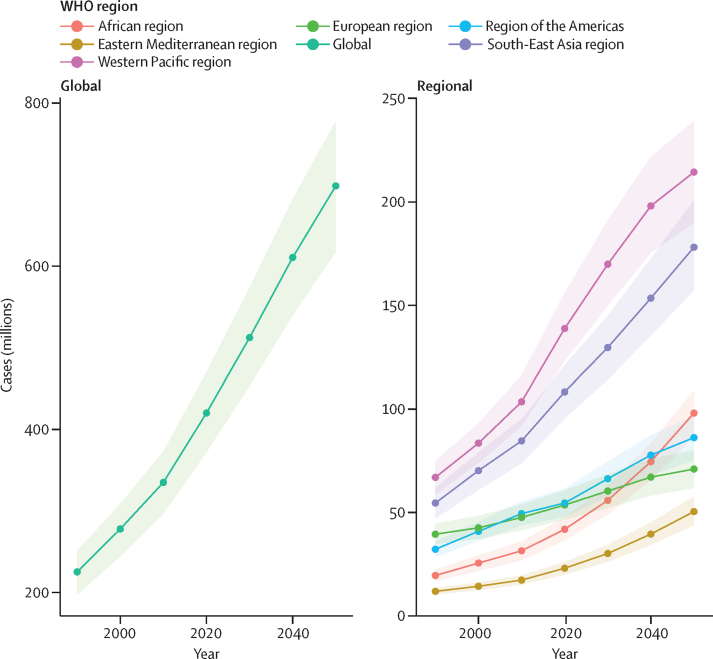
Prevalence of hearing loss 35 dB or greater, 1990–2019, with forecasts to 2050, by WHO region Shading represents 95% UI. UI=uncertainty interval.

By 2050, a projected 2·45 billion (95% UI 2·35–2·56) people will have hearing loss, a 56·1% (47·3–65·2) increase from 2019, despite stable age-standardised prevalence (appendix p 15). An estimated 698·4 million (617·7–777·8) people will have moderate-to-complete hearing loss in 2050, with most people with moderate-to-complete hearing loss residing in the Western Pacific region (214·5 million [190·1–239·1]; figure 4). The greatest percentage increase is projected in the African region, where the number of those with moderate-to-complete hearing loss is predicted to increase from 38·70 million (33·08–44·47) to 98·08 million (86·94–109·09), an increase of 154·9% (110·5–208·0). This region is followed by the Eastern Mediterranean region, where cases are projected to rise from 21·25 million (18·62–23·88) to 50·45 million (43·84–57·59), a projected increase of 138·4% (96·7–184·8).

## Discussion

Responsible for over 40 million YLDs, hearing loss was ranked as the third most common cause of YLDs in the GBD. Compared with other disease categories in the GBD, age-related and other hearing loss was the third largest cause of global YLDs in 2019 after low back pain and migraine, and was ranked first among sensory disorders. Age-related and other hearing loss was the leading cause of global YLDs compared with all causes that were explicitly modelled in the GBD for individuals older than 70 years.

Over 1·5 billion people live with hearing loss, 403 million (26%) of whom have moderate-to-complete hearing loss in their better ear. All others have mild hearing loss. Although referred to as mild, such hearing loss can cause difficulties depending on its nature and the individual's hearing needs.^6^, ^23^ This effect is especially marked in children who are developing language skills and gaining education.^23^ Among adults, those with moderate-to-severe hearing loss in the better-hearing ear are most likely to benefit from clinical attention and interventions such as hearing aids.^24^ The burden of such hearing loss is concentrated mostly in low-income and middle-income countries, which exhibit higher age-standardised rates of moderate-to complete-hearing loss compared with those of high-income countries. Moreover, the burden of disability attributable to hearing loss is concentrated in countries with poor health-care access, where hearing aid coverage is low and individuals are least likely to receive the care they need.

The distribution of individuals with moderate-to-complete hearing loss varies across regions. Geographical variation could be attributable to several factors, including the prevalence of occupational noise exposure, preventable infections such as chronic otitis media and meningitis, and health-care access. A clear association exists between ageing and hearing loss, given that most of those with hearing loss are older than 50 years. The progression of hearing loss with age also means that high-income countries have the highest all-age prevalence of hearing loss, driven mainly by their ageing population profile.

Although hearing loss is common in old ages, globally 70 million children aged 0–15 years also live with this condition. Although the prevalence of hearing loss is low in children compared with in adults, hearing loss was most severe in both the youngest and oldest individuals. When unaddressed, childhood hearing loss can affect spoken language development, literacy skills,^25^ and education, which might affect cognition and social wellbeing.^4^, ^6^, ^8^ These facts underline the importance of this affected population. Two of the causal factors that are responsible for hearing loss in children, otitis media and meningitis, are preventable. Our results align with WHO estimates that 60% of hearing loss in children is due to preventable causes such as ear infections and vaccine-preventable diseases.^26^

The clear association between hearing loss and age means that nearly everyone, if they live long enough, will have some degree of hearing loss, and at least 50% will have moderate-to-complete hearing loss requiring intervention. As the world population grows and ages over the coming decades,^22^ the need for hearing care will rise. The results of this study anticipate that by 2050, 698 million people will have moderate-to-complete hearing loss that could benefit from rehabilitation services. Rehabilitation services refer to comprehensive and holistic approaches that can include hearing aids and implants; rehabilitative therapy; sign language learning; other sensory substitutions like speechreading; and use of assistive technologies (eg, personal FM systems) and services (eg captioning). These interventions are detailed in the WHO world report on hearing.^11^ Although overall numbers are forecasted to be highest in the Western Pacific region in 2050, the greatest percentage increase is projected in the African region and the Eastern Mediterranean region.

Although hearing loss poses a substantial burden that will increase in coming years, methods of addressing this burden are available and cost-effective. Models indicate that passive screening in conjunction with hearing aid provision is an efficient intervention, with a cost of almost US$1000 per disability-adjusted life-year averted.^27^ GBD estimates suggest that there is an 83% unmet need for hearing aids globally, calculated as the proportion of individuals with moderate-to-severe hearing loss who do not use a hearing aid.^28^ Other studies show that early rehabilitation along with use of hearing devices such as cochlear implants are also cost-effective, despite large costs associated with initial technology investments and the risks of infectious complications after implantation.^29^, ^30^, ^31^, ^33^ For children with severe hearing loss, access to sign language and support for speechreading can improve access to education.^32^ The treatment of infections that can cause hearing loss (such as otitis media or meningitis) is similarly cost-effective, with variation in cost by treatment strategy.^27^ Although cost-effective interventions for hearing loss exist, they remain prohibitively expensive for health systems in low-income and middle-income countries. These methods of reducing burden should be introduced to meet high need, accompanied by scale-up of health systems and universal health coverage for effective administration of interventions.

Our study was developed in close collaboration with WHO and links closely with its world report on hearing. This report further elaborates on the public health implications of the rising prevalence of hearing loss. With the figures and projections noted in our study as its basis, the world report outlines the framework of a global public health response that is focused on integrated people-centred ear and hearing care. By the use of available technological solutions with evidence-based public health approaches, hearing loss can be prevented, identified, treated, and rehabilitated, to curtail the condition's projected increase in prevalence and overcome the adverse affects on individuals and society. This approach includes cost-effective interventions and strategies such as hearing screening for newborn babies, school-aged children, adults, and older adults as well as the use of hearing technology (eg, hearing aids or cochlear implants) and clinical management of ear diseases.^27–31,33^ Health-care services need to be prepared for the projected increases in burden, particularly in low-income and middle-income countries where ear specialists and audiologists are scarce.

We acknowledge the limitations of this study, most importantly the sparsity of the data on which these estimates are based. The lack of prevalence data is most marked in low-income countries, which have increased prevalence of severe hearing loss and insufficient access to audiometric technologies required for diagnosis. Data on hearing aid coverage is particularly sparse, with no severity-specific data outside of high-income countries and almost no data sources at all in Asia, north Africa, or Central Africa. This data gap might lead to overestimation of hearing aid coverage (and therefore underestimation of hearing loss prevalence) in low-income or under-represented locations. Moreover, we exclusively relied on NHANES data to calculate severity adjustment factors for hearing loss prevalence and split prevalence by presence of tinnitus, neglecting geographical variation in the prevalence of hearing loss and tinnitus. Additional research is needed to gain a deeper understanding of hearing loss prevalence, concurrence with tinnitus, and hearing aid coverage in areas where data are limited.

Data on the causes of hearing loss are also sparse because few surveys report prevalence of hearing loss by underlying cause. This difficulty presents challenges because the causes of hearing loss differ substantially in children compared with in adults.^26^, ^34^ Because more data exist on the cause of hearing loss in children, we scaled cause-specific hearing loss prevalence to total prevalence up to age 20 years. After this age, we treated age-related and other hearing loss as the residual between total prevalence and other causes of hearing loss. This residual approach has the potential to mask the contribution of other non-age factors to hearing loss burden. Moreover, other causes of hearing loss such as labour complications, vaccine-preventable diseases, non-occupational noise exposure, diabetes,^35^ and ototoxic medications such as aminoglycosides were not accounted for in our estimates because of insufficient data. Strengthened data on the causes of hearing loss in older ages (as opposed to assuming that all hearing loss in old age is age-related) would improve this method.

Although meta-regression tools enable us to leverage information from data-rich locations to estimate prevalence in locations with no data, data sparsity can make distinguishing between true variation in prevalence and measurement error particularly difficult. This issue is compounded by few predictive covariates or risk factors, because the association between hearing loss and other factors (like noise exposure) is not well quantified and likely to be masked by age. Data on self-reported hearing loss or audiometric data recorded with mobile technologies could potentially assess disease burden in locations where audiologists are sparse. However, telehealth methodologies are not yet widely used, and the generalisability of self-reported data is unclear.

GBD estimates of hearing loss are adjusted for hearing aid coverage, shifting the proportion of people with a hearing aid in each severity category to the category directly below. This adjustment might be crude in nature, masking the effect of hearing health care in reducing disability and neglecting variation in treatment effect between individuals. Whereas providers in eye care refer to best-corrected vision loss to mobilise resources towards those who are most amenable to treatment, no complementary best-corrected measure for hearing loss exists. Acquisition of data on best-corrected hearing loss could contribute to an understanding of the proportion of individuals with hearing loss who are amenable to intervention.

A systematic review of hearing loss prevalence will be completed for GBD 2020 to identify new sources of information in data-sparse locations. Additionally, we aim to revise modelling methods in upcoming years, assessing how prevalence is adjusted on the basis of hearing aid coverage, the proportion of individuals with hearing loss concurrent with tinnitus, and the severity distribution of hearing loss due to otitis media. We aim to forecast the avoidable burden of hearing loss with rapid implementation of hearing health-care interventions, provided additional data on intervention coverage is obtained. This forecast could provide policy makers with tools to effectively allocate hearing health-care services and plan for future scenarios.

The results of this study point to a growing public health challenge, which needs global attention and a definitive response. The increasing prevalence of hearing loss, high contribution to global disability, and large unmet need for hearing aids together serve as a call for urgent attention. Cost-effective, preventive, and treatment solutions to mitigate the effects of hearing loss exist, including childhood screening programmes, earplugs and other noise-reduction technologies, hearing devices, and early treatment of otitis media and meningitis. Health system capacity must be scaled up to address growing needs, particularly in low-income settings.

Correspondence to: Prof Theo Vos, Institute for Health Metrics and Evaluation, University of Washington, Seattle, WA 98195, USA tvos@uw.edu

## Data sharing

Data presented in this manuscript are made publicly available at https://vizhub.healthdata.org/gbd-compare.
