# Supplementary material for: Hearing loss prevalence and years lived with disability, 1990–2019: findings from the Global Burden of Disease Study 2019
Source: Lancet. 2021 Mar 13;397(10278):996–1009. doi: 10.1016/S0140-6736(21)00516-X (PMC7960691; doi:10.1016/S0140-6736(21)00516-X)
Supplement: Supplementary appendix [file mmc1.pdf]

THE LANCET

Supplementary appendix

This appendix formed part of the original submission and has been peer reviewed. We post it as supplied by the authors.

Supplement to: GBD 2019 Hearing Loss Collaborators. Hearing loss prevalence and years lived with disability, 1990–2019: findings from the Global Burden of Disease Study 2019. *Lancet* 2021; **397:** 996–1009.

Appendix 1 Supplementary methods and results to "Global hearing loss prevalence and years lived with disability, 1990–2019: findings from the Global Burden of Disease Study 2019"

This appendix provides supplemental figures and more detailed results for "Global hearing loss prevalence and years lived with disability, 1990–2019: findings from the Global Burden of Disease Study 2019."

Portions of this appendix have been reproduced or adapted from Vos et al.[1] References are provided for reproduced sections.

Table of contents

List of figures and tables 2

Figures 2

Tables 2

Section 1. Statement of GATHER compliance 3

Section 2. Data seeking and access 3

Section 2.1. Hearing loss and hearing aid coverage input data[1] 3

Section 2.2. Data access 3

Section 3. Supplemental methods[1] 3

Section 3.1. Input data processing 3

Section 3.2 Adjusting for hearing aid coverage 4

Section 3.3. Modelling hearing loss by aetiology 4

Section 3.4. Modelling hearing loss with and without tinnitus 5

Section 3.5. Modelling hearing loss attributable to occupational noise exposure 5

Section 4. References 5

Section 5. Figures and tables 6

Figure S1. WHO member states by region 6

Figure S2. Geographical distribution of hearing loss prevalence input data before outliering 7

Figure S3. Geographical distribution of input data of hearing aid coverage data before outliering 7

Figure S4. Plot of both sex prevalence data pre- and post- sex split, hearing loss 20-34 dB, with 95% confidence intervals 8

Figure S5. Flow chart of hearing aid adjustment methodology 8

Figure S6. Map of age-standardised hearing loss prevalence (per 100,000 persons), 2019, by country, all severities 9

Figure S7. Map of age-standardised hearing loss prevalence (%), 2019, by country, all severities 9

Figure S8. Proportion of moderate to complete hearing loss cases, by age and severity, 2019 10

Figure S9. Cases of hearing loss, 1990–2019, both sexes, all severities, with forecasts to 2050, globally and by WHO region, with 95% UIs 11

Table S1. Sex split model coefficients for hearing loss and hearing aid coverage data, log space, with 95% UIs 11

Table S2. MR-BRT cross-walk adjustment factors for data adjustment, by severity category, with 95% UIs 12

Table S3. Covariates in Dismod-MR 2.1 models, exponentiated beta coefficients, with 95% UIs 14

Table S4. Age-standardised prevalence (per 1) and cases of hearing loss (in thousands), by country and severity, 2019, with 95% UIs 14

Table S5. Linear Regression of age-standardised YLD rate and Healthcare Access and Quality (HAQi), model results 14

Table S6. Age-standardised prevalence (per 1) and cases of hearing loss, projected from 2020 to 2050, by severity and WHO region 15

Table S7. GATHER checklist 16

Author's Contributions 17

List of figures and tables

Figures and tables are available in section 5.

Figures

Figure S1. WHO member states by region
Figure S2. Geographical distribution of hearing loss prevalence input data before outliering
Figure S3. Geographical distribution of hearing aid coverage input data before outliering
Figure S4. Plot of both sex prevalence data pre- and post- sex split, hearing loss 20-34 dB, with 95% confidence intervals
Figure S5. Flow chart of hearing aid adjustment methodology
Figure S6. Map of age-standardised hearing loss prevalence (per 100,000 persons), 2019, by country, all severities
Figure S7. Map of age-standardised hearing aid coverage (%), 2019, by country, all severities
Figure S8. Proportion of moderate to complete hearing loss cases, by age and severity, 2019
Figure S9. Cases of hearing loss, both sexes, all severities, 1990–2019, with forecasts to 2050, globally and by WHO region, with 95% UIs

Tables

Table S1. Sex split model coefficients for hearing loss and hearing aid coverage data, log space, with 95% UIs
Table S2. MR-BRT cross-walk adjustment factors for data adjustment, by severity category, with 95% UIs
Table S3. Covariates in Dismod-MR 2.1 models, exponentiated beta coefficients, with 95% UIs
Table S4. Age-standardised prevalence (per 1) and cases of hearing loss (in thousands), by country and severity, 2019, with 95% UIs
Table S5. Linear regression of age-standardised YLD rate and Healthcare Access and Quality (HAQi), model results
Table S6. Age-standardised prevalence (per 1) and cases of hearing loss, projected from 2020 to 2050, by severity and WHO region
Table S7. GATHER checklist

Section 1. Statement of GATHER compliance

This study complies with the Guidelines for Accurate and Transparent Health Estimates Reporting (GATHER) recommendations (appendix section 5, table 4 with GATHER checklist).[2]

Section 2. Data seeking and access

Section 2.1 Hearing loss and hearing aid coverage input data[1]

To model hearing loss we utilised prevalence data from population-representative surveys. We obtained data sources up to 2008 from a previously published systematic review[7]. In GBD 2013, we conducted a systematic review using the following PubMed search string:

(hearing loss[Title/Abstract] OR deafness[Title/Abstract] OR hearing loss[Title/Abstract]) AND (prevalence[Title/Abstract]) AND ("2008"[PDAT] : "3000"[PDAT]) AND (cross sectional OR survey)

An additional systematic review was conducted during GBD 2016 using the following PubMed search string:

(hearing loss[Title/Abstract] OR deafness[Title/Abstract] OR hearing loss[Title/Abstract] OR audiometry[Title/Abstract]) AND (prevalence[Title/Abstract]) AND ("2008/11/26"[PDAT]: "3000"[PDAT]) AND (cross sectional OR survey)

This search was conducted on November 30, 2016. The search obtained 239 results, of which. 17 were accepted and added to hearing loss models.

We utilized systematic reviews and self-reported survey data to estimate hearing aid coverage. Survey sources included the WHO Multi-Country Survey Study on Health and Responsiveness (MCSS), the WHO Studies on Global Ageing and Adult Health (SAGE), and the Integrated Public Use Microdata Series (IPUMS).

Section 2.2 Data access

Data inputs are made publicly available through the Global Health Data Exchange (http://ghdx.healthdata.org/). Analytical code is stored at https://github.com/ihmeuw/.

Section 2.3 Construction of hearing loss disability weights

Hearing loss disability weights were derived from various surveys, in which participants were asked to make pairwise comparisons of different health conditions described in lay language with less than 30 words. Survey respondents valued hearing health states with tinnitus as “worse” than hearing health states without tinnitus, justifying the relatively higher disability weights associated with these states[8].

The moderate hearing loss category was split into moderate and moderately severe hearing loss per guidance from expert GBD hearing loss collaborators after health states were already established for the disability weight surveys used to derive disability weights. To construct this modified disability weight, we averaged the disability weights for the categories immediately above and below, but no lay description was added.

Section 3. Supplemental methods[1]

Section 3.1 Input data processing

To sex-split input data, we ran a MR-BRT model on the log ratio of female/male prevalence (Appendix 5 Table S1). MR-BRT methodology is described in detail elsewhere1. The exponentiated form of the model coefficients (betas) is the ratio of female to male prevalence within the dataset, which is then applied to “both” sex data-points to split into male and female data-points (representative example in Supplemental Figure 4). A negative coefficient means that female prevalence is lower than male prevalence, and a positive coefficient means that female prevalence is higher than male prevalence.

Input data were then adjusted to reference GBD severity categories, utilising data from the National Health and Nutrition Examination Survey (NHANES)[6]. Data from the 1999–2000, 2001–2002, 2003–2004, 2005–2006, 2007–2008, and 2009–2010 iterations of the survey were utilised because audiometry examinations were included in these years. NHANES utilized pure-tone air conduction audiometry for both ears of each participant. We aggregated NHANES microdata by age and sex to calculate the prevalence of each reference and alternative severity category. We then ran a MR-BRT model on the logit difference between the prevalence of an alternate category and its corresponding reference category to derive adjustment factors (Appendix 5 Table S2). A negative coefficient

means that the prevalence of the alternative category was less than the prevalence of the reference category, and data were adjusted upwards. A positive coefficient means that the prevalence of the alternative severity category was higher than the reference category, and data were adjusted downwards.

Section 3.2 Adjusting for hearing aid coverage

To adjust prevalence estimates for hearing aid coverage, we first modelled hearing aid coverage, based on population-representative surveys of hearing-impaired individuals. Hearing aid coverage was calculated as the proportion of hearing-impaired individuals who stated that they utilised a hearing aid. We utilized 35 population-representative studies from 28 countries. Data was primarily concentrated in Western Europe (18 sources), followed by High-income North America (8). The geographic distribution of data sources is visualised in Supplemental Figure 3.

Hearing aid coverage data were pooled in DisMod-MR 2.1 to estimate hearing aid coverage for each location, age, and sex. Socio-demographic Index (SDI) was a covariate in the model and predictive of hearing aid coverage (Supplemental Table 3).

To calculate severity-specific hearing aid coverage, we utilised microdata from the NHANES and Nord-Trondelag surveys,[3] which provided hearing coverage information by age, sex, and severity. We ran a logistic regression on age with binary indicators for severity and sex to calculate the proportion of individuals who used a hearing aid for each age, sex, and severity (equation shown below):

$$logit(coverage_{Norway\ and\ US}) = \beta age + \beta sev + \beta sex$$

We assumed that individuals with complete hearing loss did not use a hearing aid.

We then calculated the severity-specific hearing aid coverage rate for each location. We scaled estimates of total hearing aid coverage from our DisMod model by dividing the value produced in each location by the value produced for Norway. This was done to correct for any bias created by using adjustment factors calculated with predominantly Norway data. This was additionally done to account for differences in hearing aid coverage by SDI, as our severity-specific data comes exclusively from high-income countries. We multiplied the scaled hearing aid coverage value for each location by each of the six proportions of severity-specific coverage. This gave us the proportion of individuals in each severity category who use a hearing aid (equation below):

$$coverage_{location,severity} = coverage_{Norway\ and\ US,severity} * \frac{coverage_{location,all\ severities}}{coverage_{Norway,all\ severities}}$$

A flow chart illustrating this adjustment is found in Appendix 5 (Supplemental Figure 5).

Section 3.3. Modelling hearing loss by aetiology

We assumed that all hearing loss occurring at the time of birth was due to congenital abnormalities.

Chronic otitis was modelled as using data from surveys and published literature. The most recent systematic review of otitis media prevalence was conducted for GBD 2019 utilizing the following PubMed search string:

(otitis media[Title/Abstract] AND (inciden*[Title/Abstract] OR prevalen*[Title/Abstract] OR remission[Title/Abstract] OR duration[Title/Abstract]) AND ("2017/10/01"[PDAT] : "3000"[PDAT]) NOT (animals[MESH] NOT humans[MESH]))

Studies that were not population-representative, not a source of primary data, or had a sample size under 150 were excluded. In total, 83 sources from 27 countries were utilised for modelling.

Chronic otitis media data were sex-split, then pooled in DisMod-MR 2.1. Lag-distributed income (LDI) was utilised as a covariate (exponentiated beta= .63 [95% UI: .61–.67]). We assumed chronic otitis media cases resulted in either mild or moderate hearing loss, based on the distribution of mild and moderate hearing loss cases reported in Fria and colleagues 1985.5

Meningitis was modelled using 925 inpatient hospital data, inpatient claims data, surveillance data, and published literature sources from 108 countries. The most recent systematic review was conducted for GBD 2019, utilising the following PubMed search string:

("meningitis"[MeSH Terms] OR "meningitis"[Title/Abstract]) AND ("case fatality rate"[Title/Abstract] OR "mortality"[MeSH Terms] OR "mortality"[Title/Abstract] OR "fatality"[Title/Abstract]) NOT ("animals"[MeSH Terms] NOT "humans"[MeSH Terms]) AND (1990[DP] : 3000[DP]) AND ("Meningitis, Haemophilus"[MeSH Terms] OR "Haemophilus"[Title/Abstract] OR "Meningitis, Pneumococcal"[MeSH Terms] OR "Pneumococcal"[Title/Abstract] OR "Meningitis, Meningococcal"[MeSH Terms] OR "Meningococcal"[Title/Abstract] OR "Meningitis, Viral"[MeSH Terms] OR "Viral"[Title/Abstract] OR "Streptococcus agalactiae"[MeSH Terms] OR "Streptococcus agalactiae"[Title/Abstract]).

This search returned 2983 results, of which 134 were accepted.

All meningitis sequelae, including hearing loss, were estimated by applying post-discharge proportions of health consequences calculated by a meta-analysis by Edmond and colleagues.[4] Details of this split have been described elsewhere.[1]

Section 3.4. Modelling hearing loss with and without tinnitus

Data on the proportion of individuals with hearing loss who had tinnitus were obtained from NHANES surveys[6]. Data from years 1999–2000, 2001–2002, 2003–2004, and 2011–2012 were included. For individuals with mild hearing loss, the occurrence of ringing at least once a month was defined as a mild hearing loss with tinnitus case. For moderate through severe hearing loss, ringing, roaring, or buzzing "at least once a day" was defined as a moderate hearing loss with tinnitus case. For complete hearing loss, individuals who responded that they "almost always" had ringing was defined as a complete hearing loss with tinnitus case.

NHANES data were utilised to calculate confidence intervals, assuming a binomial distribution. Proportions were then applied to prevalence estimates to split into hearing loss with and without tinnitus.

Section 3.5. Modelling hearing loss attributable to occupational noise exposure

Occupational noise exposure was defined as the proportion of the population occupationally exposed to 85+ dB of noise, based on population distributions across 17 economic activities. Data from the International Labour Organization were used to estimate employment to population ratios and proportions of the population employed in different economic activities, by location, age, sex, and year. The development status of each location then informed the estimate of the rate at which workers in each economic activity were exposed to occupational noise. Exposure was estimated for ages 15 and older, using spatiotemporal Gaussian process regression (ST-GPR) models. Methods have been described in greater detail elsewhere[1].

The relative risk of hearing loss among those with occupational noise exposure was estimated based on data from published meta-analyses. The most recent systematic review was completed in GBD 2016. The theoretical minimum-risk exposure level was assumed to be exposure to less than 85 dB of noise. The population attributable fraction for occupational noise exposure was calculated utilising the formula as outlined in the GBD 2019 methods Appendix[1].

Section 4. References

1 Vos T, Lim SS, Abbafati C, *et al.* Global burden of 369 diseases and injuries in 204 countries and territories, 1990–2019: a systematic analysis for the Global Burden of Disease Study 2019. *The Lancet* 2020; **396**: 1204–22.

2 Stevens GA, Alkema L, Black RE, *et al.* Guidelines for Accurate and Transparent Health Estimates Reporting: the GATHER statement. *The Lancet* 2016; **388**: e19–23.

3 Engdahl B, Tambs K, Borchgrevink HM, Hoffman HJ. Screened and unscreened hearing threshold levels for the adult population: results from the Nord-Trøndelag Hearing Loss Study. *Int J Audiol* 2005; **44**: 213–30.

4 Edmond K, Clark A, Korczak VS, Sanderson C, Griffiths UK, Rudan I. Global and regional risk of disabling sequelae from bacterial meningitis: a systematic review and meta-analysis. *Lancet Infect Dis* 2010; **10**: 317–28.

5 Fria TJ, Cantekin EI, Eichler JA. Hearing Acuity of Children With Otitis Media With Effusion. *Arch Otolaryngol* 1985; **111**: 10–6.

6 NHANES Survey Methods and Analytic Guidelines. https://wwwn.cdc.gov/nchs/nhanes/analyticguidelines.aspx (accessed Oct 27, 2020).

7 Pascolini D, Smith A. Hearing Impairment in 2008: a compilation of available epidemiological studies. Int J Audiol 2009; 48: 473–85.

8 Salomon JA, Haagsma JA, Davis A, et al. Disability weights for the Global Burden of Disease 2013 study. Lancet Glob Health 2015; 3: e712-723.

Section 5. Figures and tables

Figure S1. WHO member states by region

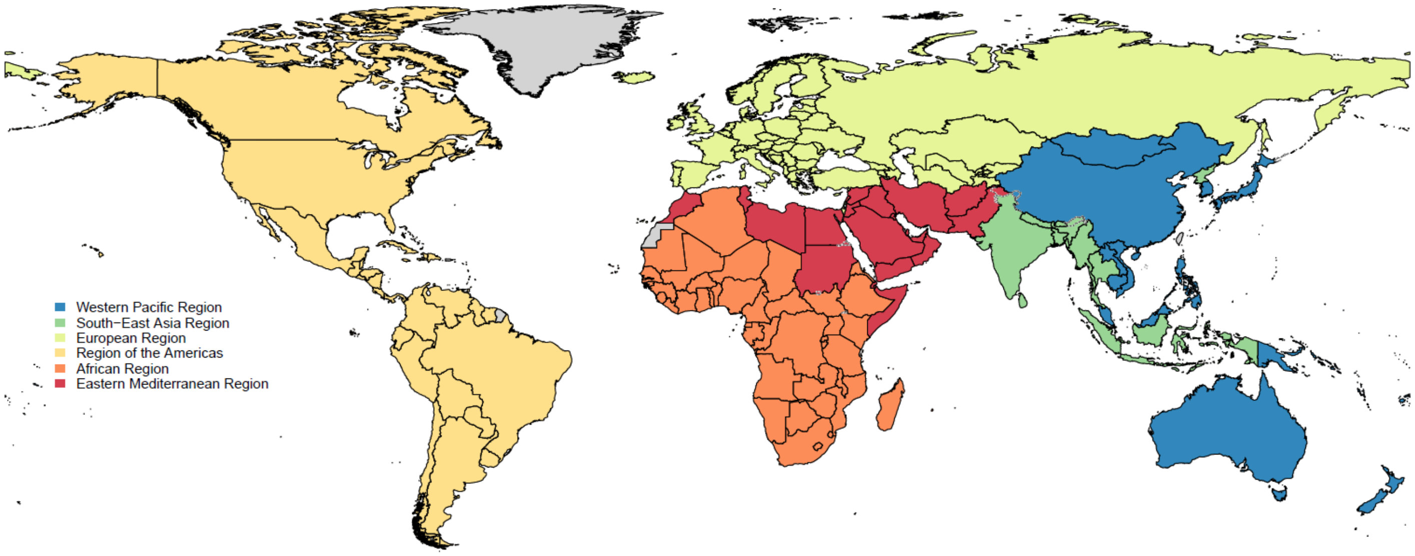

Figure S2. Geographical distribution of hearing loss prevalence input data before outliering

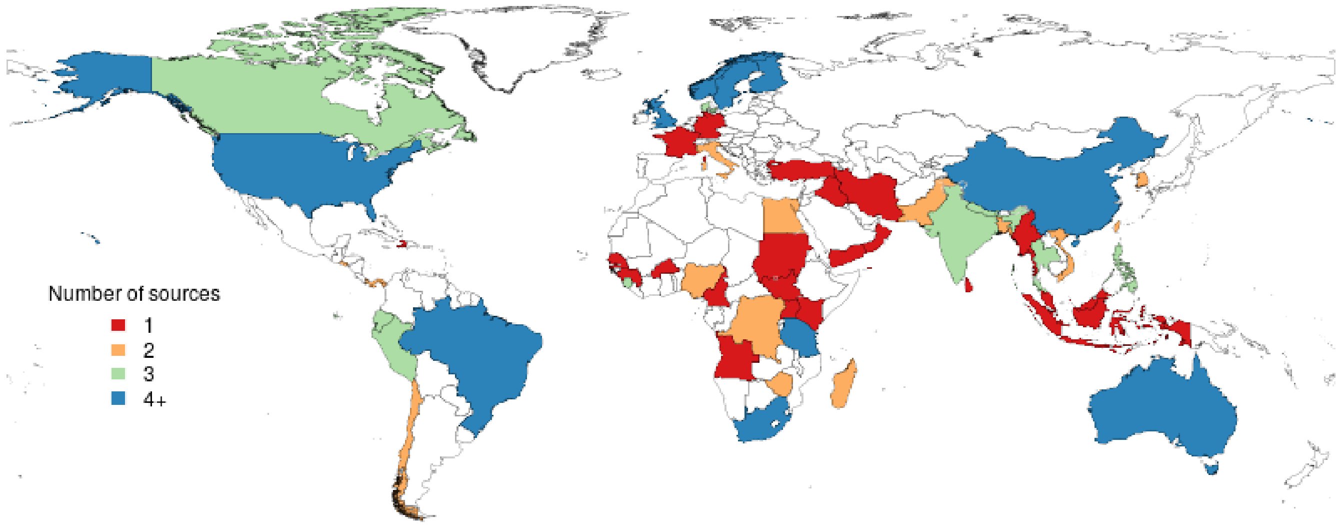

Figure S3. Geographical distribution of input data of hearing aid coverage data before outliering

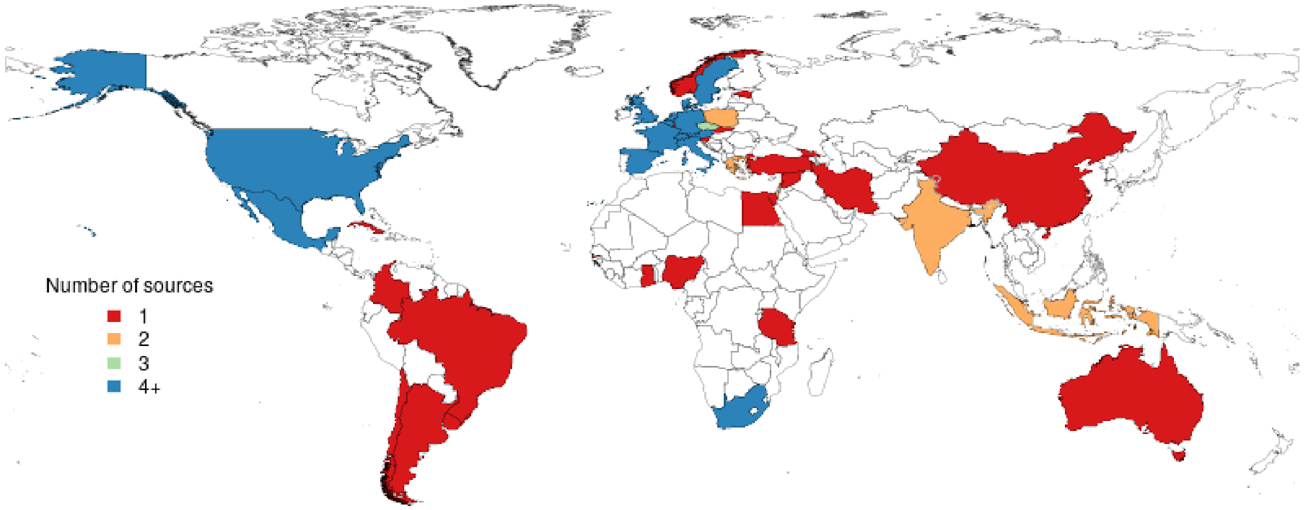

Figure S4. Plot of both sex prevalence data pre- and post- sex split, hearing loss 20–34 dB, with 95% uncertainty intervals

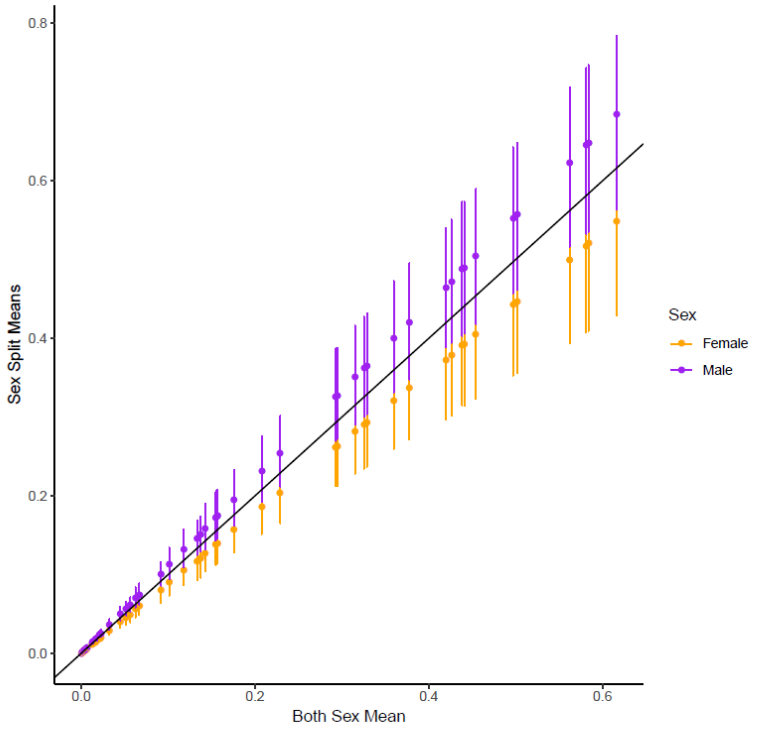

Figure S5. Flow chart of hearing aid adjustment methodology

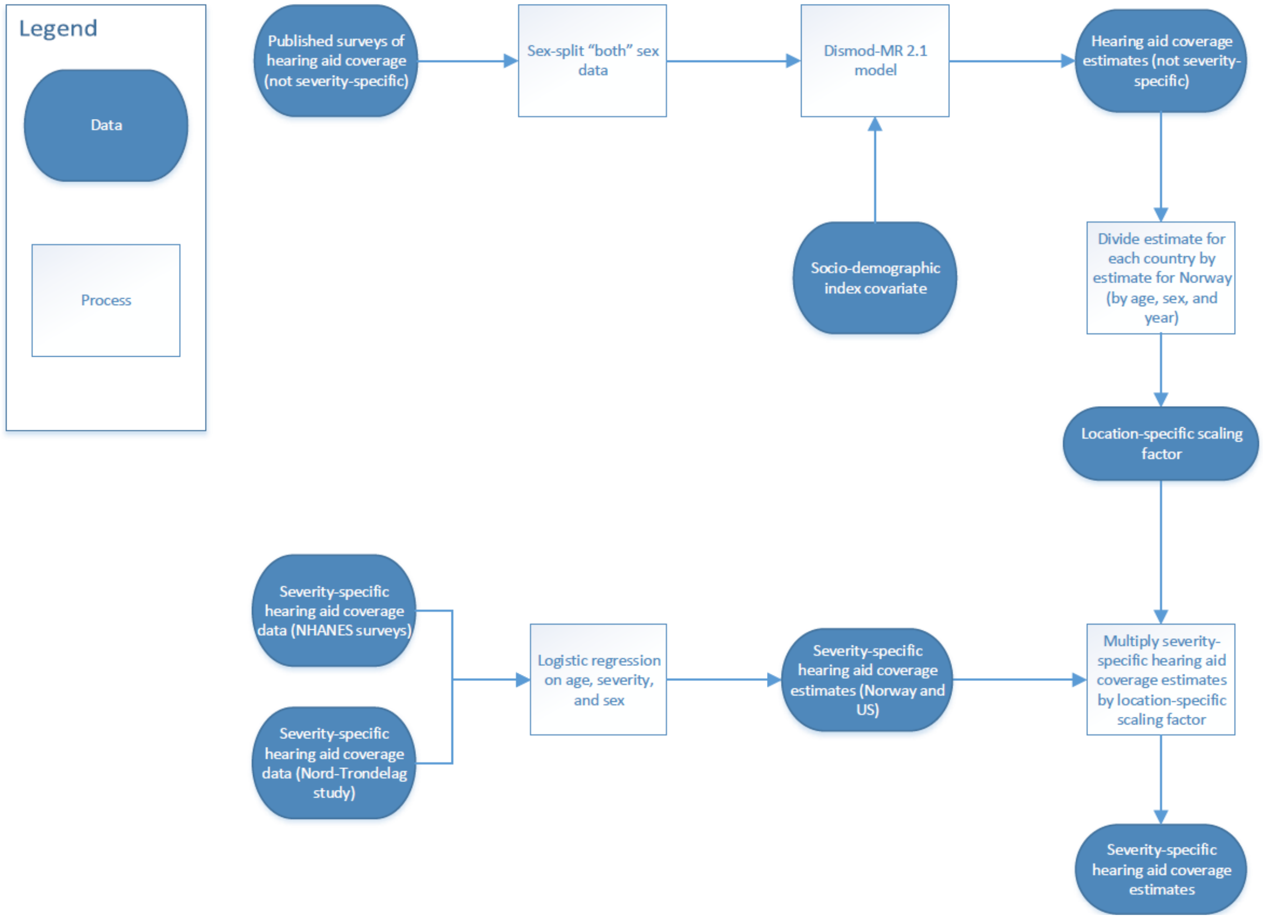

Figure S6. Map of age-standardised hearing loss prevalence (per 100,000 persons), 2019, by country, all severities

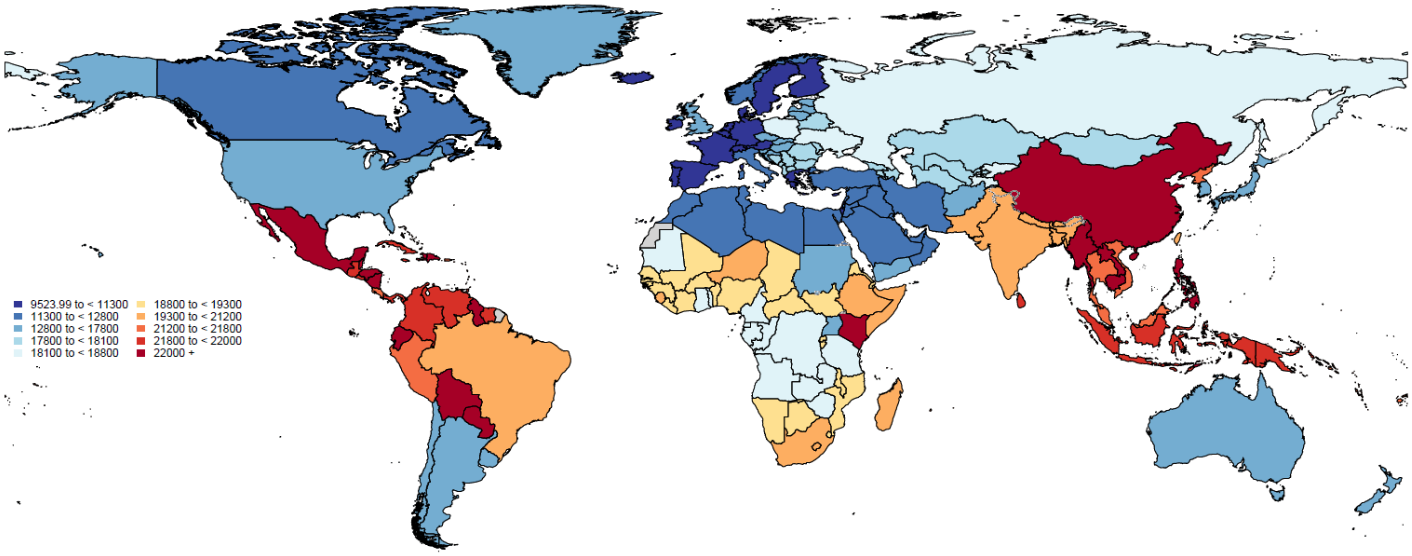

Figure S7. Map of age-standardised hearing aid coverage (%), 2019, by country, all severities

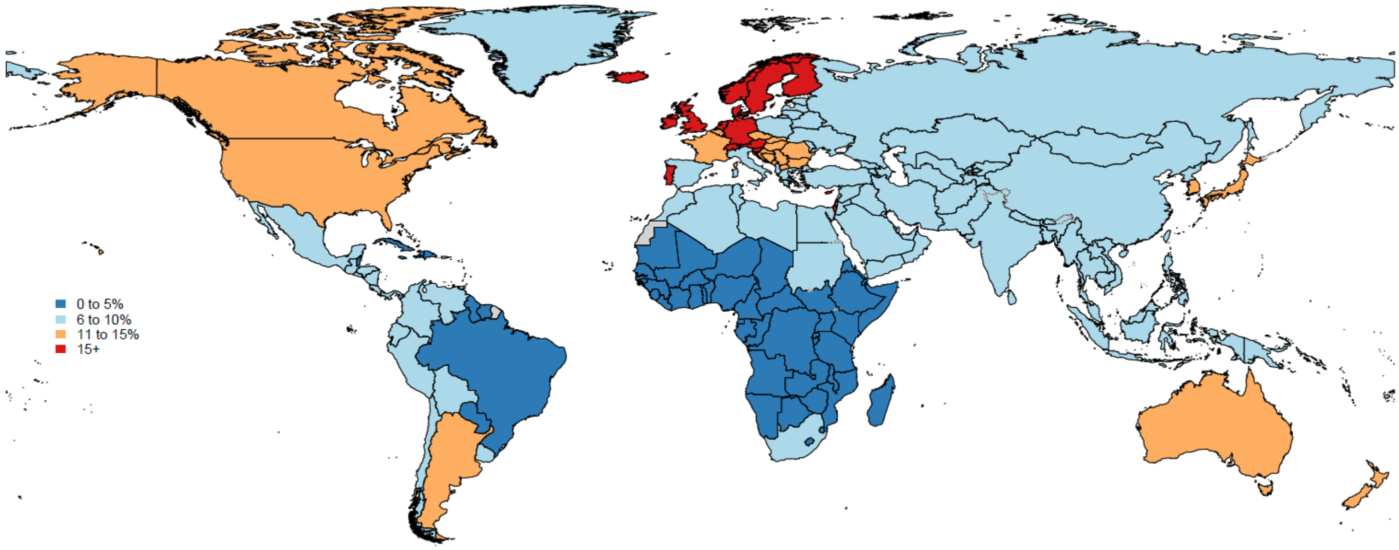

Figure S8. Proportion of moderate to complete hearing loss cases, by age and severity, 2019

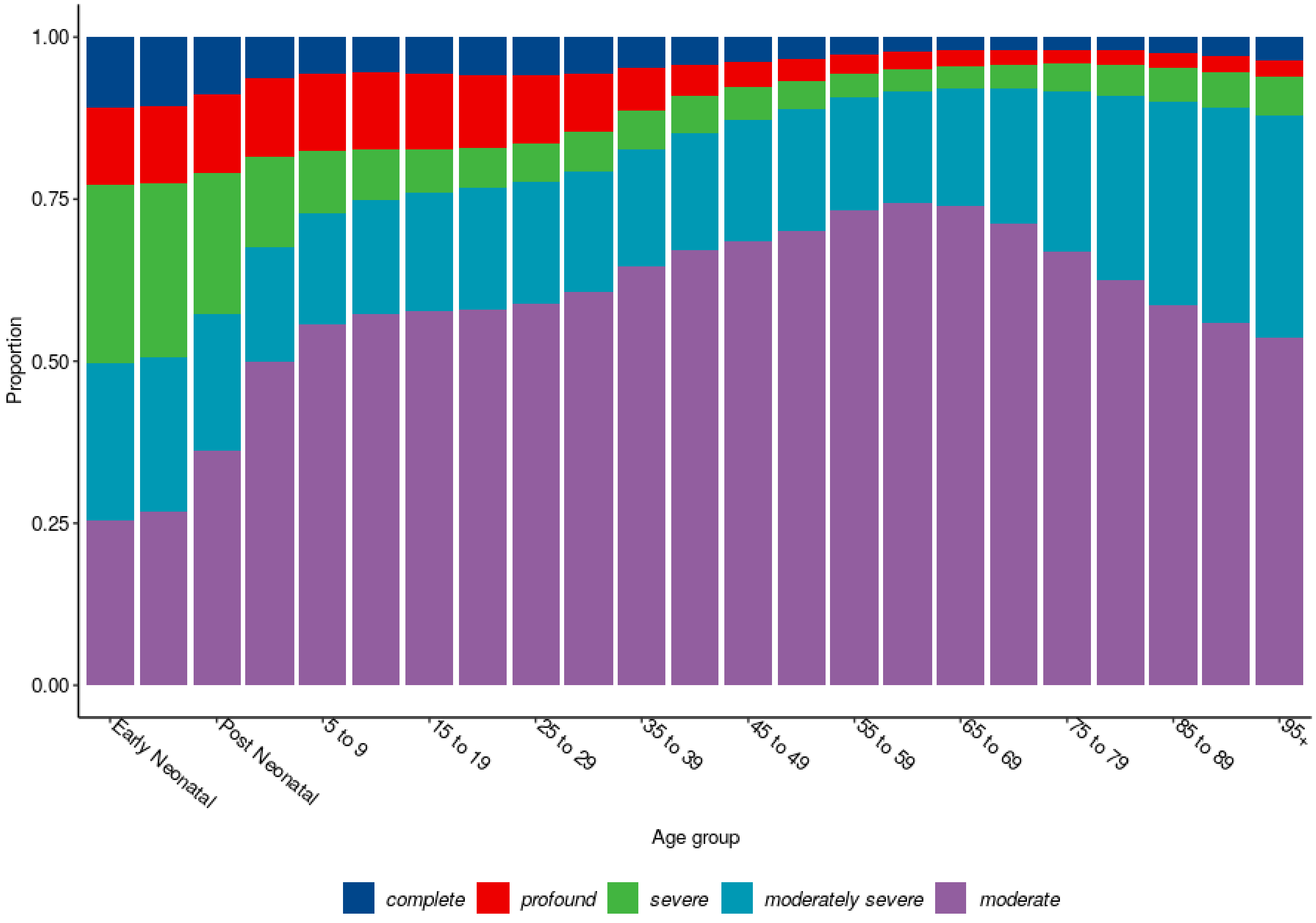

Figure S9. Cases of hearing loss, 1990–2019, both sexes, all severities, with forecasts to 2050, globally and by WHO region, with 95% UIs

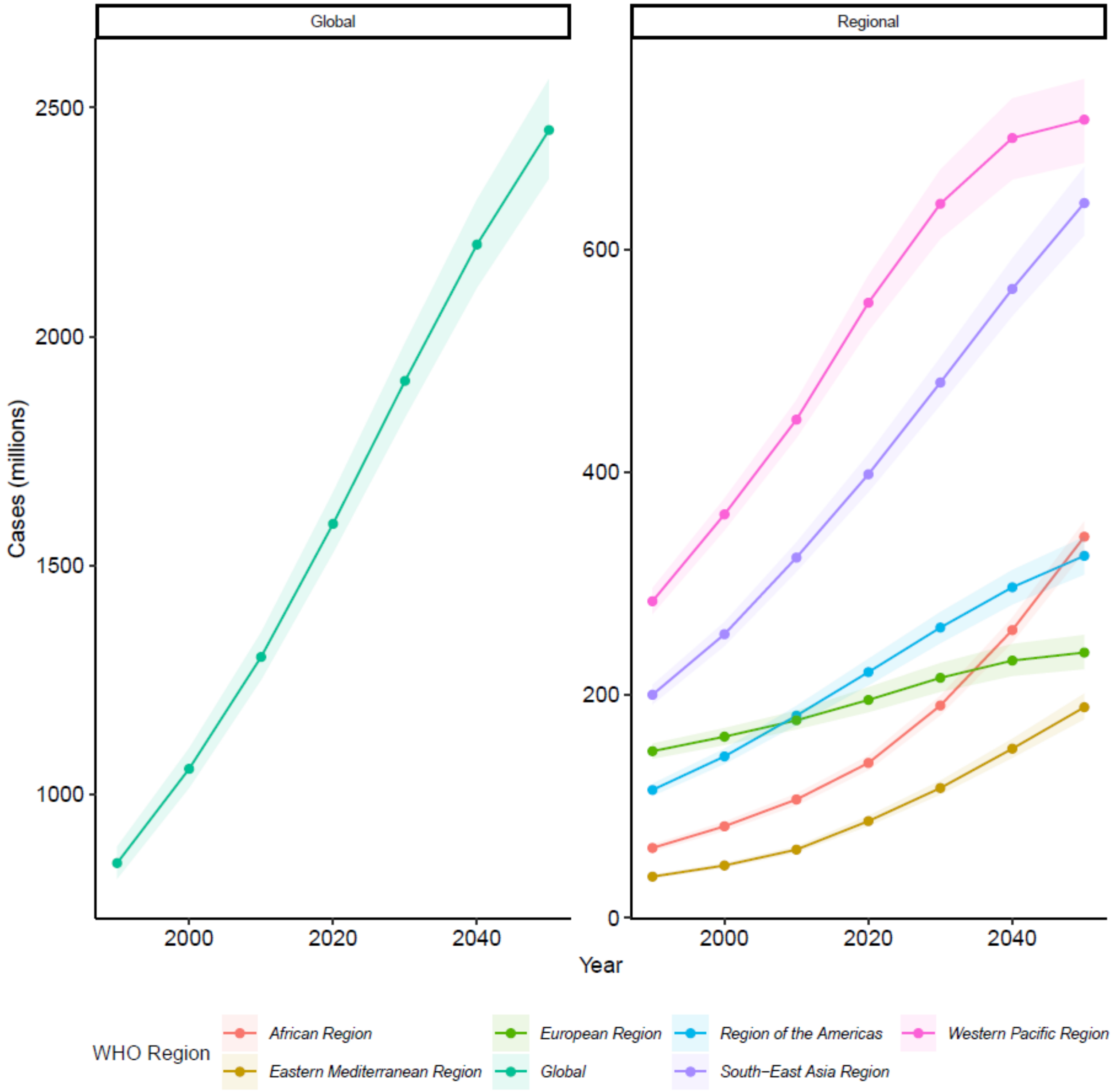

Table S1. Sex split model coefficients for hearing loss and hearing aid coverage data, log space, with 95% UIs

Model	Coefficient (95% UI)
Hearing Loss, 0-19 dB	0.0171 (-.0163 — 0.0505)
Hearing Loss, 20-34 dB	-0.211 (-0.583 — 0.161)
Hearing Loss, 35-49 dB	-0.240 (-0.511 — 0.031)

Hearing Loss, 35+ dB	-0.139 (-0.48 — 0.202)
Hearing Loss, 50-64 dB	-0.468 (-0.629 — -0.307)
Hearing Loss, 65-79 dB	-0.383 (-0.536 — -0.230)
Hearing Loss, 80-94 dB	-0.0738 (-0.562 — 0.414)
Hearing Loss, 95+ dB	-0.149 (-0.317 — 0.019)
Hearing aids (proportion)	-0.0176 (-0.146 — 0.111)

Table S2. MR-BRT cross-walk adjustment factors for data adjustment, by severity category, with 95% UIs

Reference category (dB)	Alternate category (dB)	Beta coefficient, logit (95% CI)
**0–19**	0-24	0.60 (0.54 to 0.67)
	0-25	0.70 (0.64 to 0.77)
	0-29	1.13 (0.68 to 1.59)
	0-30	1.24 (0.83 to 1.68)
	0-39	1.67 (-0.04 to 3.58)
	0-40	1.71 (-0.05 to 3.53)
**20–34**	0-24	3.40 (-1.46 to 8.28)
	0-25	3.49 (-1.53 to 8.29)
	0-29	3.82 (-0.85 to 8.29)
	0-30	3.89 (-0.24 to 8.42)
	0-39	4.48 (0.61 to 8.55)
	0-40	4.50 (0.86 to 8.14)
	20-39	0.27 (0.02 to 0.52)
	20-40	0.29 (0.003 to 0.59)
	20-200	0.52 (-0.35 to 1.32)
	21-39	0.12 (-0.29 to 0.52)
	25-39	-0.39 (-1.04 to 0.34)
	26-40	-0.50 (-1.36 to 0.28)
	26-99	-0.03 (-1.65 to 1.73)
	26-200	-0.03 (-1.74 to 1.54)
	30-40	-1.06 (-2.24 to 0.007)
	30-200	-0.37 (-2.12 to 1.43)
**35–49**	0-39	5.18 (0.16 to 10.08)
	0-40	5.24 (0.41 to 10.17)
	20-39	1.45 (0.04 to 2.85)
	20-40	1.49 (0.10 to 2.88)
	21-39	1.31 (0.02 to 2.67)
	25-39	0.76 (-0.27 to 1.93)
	26-40	0.67 (-0.30 to 1.75)
	30-40	0.09 (-0.89 to 1.05)
	31-50	0.10 (0.29 to 0.74)
	40-64	-0.10 (-0.85 to 0.61)
	40-69	-0.04 (-0.82 to 0.811)
	41-55	-0.45 (-1.06 to 0.23)
	41-60	-0.29 (-0.99 to 0.37)
	41-70	-0.12 (-1.06 to 0.76)
**50–64**	40-64	1.13 (0.58 to 1.68)
	40-69	1.22 (0.64 to 1.80)
	41-55	0.72 (-0.09 to 1.53)
	41-60	0.92 (0.30 to 1.55)
	41-70	1.13 (0.49 to 1.77)

	51-70	0.06 (-0.31 to 0.42)
	55-69	-0.42 (-1.00 to 0.15)
	56-70	-0.43 (-1.10 to 0.24)
**65–79**	40-69	2.44 (0.92 to 3.99)
	51-70	1.35 (0.01 to 2.68)
	55-69	0.86 (-0.53 to 2.24)
	56-70	0.84 (-0.47 to 2.16)
	61-80	0.35 (-0.04 to 0.72)
	61-99	0.46 (0.17 to 0.75)
	65-84	0.03 (-0.01 to 0.08)
	70-89	-0.20 (-0.63 to 0.22)
	70-94	-0.20 (-0.62 to 0.24)
	70-95	-0.20 (-0.63 to 0.23)
	71-90	-0.26 (-0.86 to 0.34)
	71-99	-0.16 (-0.75 to 0.44)
	71-200	-0.19 (-0.81 to 0.42)
**80–94**	61-99	1.58 (-0.42 to 3.58)
	65-84	0.92 (-0.89 to 2.73)
	70-89	0.54 (-1.06 to 2.14)
	70-94	0.44 (-1.01 to 1.88)
	70-95	0.44 (-1.00 to 1.89)
	71-90	0.25 (-0.96 to 1.45)
	71-99	0.37 (-0.83 to 1.58)
	71-200	0.41 (-0.88 to 1.71)
	80-200	0.00 (-0.04 to 0.04)
	81-99	-3.92e-16 (-0.04 to 0.03)
	81-200	0.00 (-0.04 to 0.04)
	85-200	-4.37e-24 (-0.04 to 0.04)
	90-99	0.00 (-0.03 to 0.03)
	90-200	0.00 (-0.03 to 0.03)
**35–200**	20-200	1.79 (1.48 to 2.10)
	26-200	1.02 (0.73 to 1.31)
	26-99	1.02 (0.73 to 1.31)
	30-200	0.55 (0.40 to 0.70)
	31-200	0.43 (0.33 to 0.54)
	31-99	0.44 (0.34 to 0.54)
	40-200	-0.49 (-0.58 to -0.39)
	40-99	-0.48 (-0.59 to -0.38)
	41-200	-0.59 (-0.78 to -0.39)
	41-99	-0.58 (-0.78 to -0.39)
**95–200**	61-99	2.42 (0.84 to 4.03)
	71-99	0.65 (-1.14 to 2.43)
	71-200	0.60 (-1.13 to 2.33)
	80-200	0.08 (-0.34 to 0.52)
	81-99	0.08 (-0.35 to 0.50)
	81-200	0.05 (-0.30 to 0.41)
	85-200	0.00 (-0.04 to 0.04)
	90-99	0.00 (-0.02 to 0.02)
	90-200	0.00 (-0.02 to 0.02)
	91-99	0.00 (-0.03 to 0.02)
	91-200	0.00 (-0.02 to 0.02)
	95-99	0.00 (-0.02 to 0.02)
	96-99	0.00 (-0.02 to 0.02)

Table S3. Covariates in Dismod-MR 2.1 Models, exponentiated beta coefficients, with 95% UIs

**Model**	**Covariate name**	**Measure**	**Exponentiated beta (95% UI)**
Hearing loss impairment at 0-19 dB	Socio-demographic Index	Prevalence	1.01 (1.00 — 1.03)
Hearing loss impairment at 35+ dB	Socio-demographic Index	Prevalence	0.20 (0.15 — 0.28)
Hearing loss impairment at 95+ dB	Socio-demographic Index	Prevalence	0.30 (0.16 —0.57)
Hearing aids (proportion of total hearing loss)	Socio-demographic Index	Proportion	2.55 (2.22 — 2.71)

Table S4. Age-standardised prevalence (per 1) and cases of hearing loss (in thousands), by country and severity, 2019, with 95% UIs

Attached to end of document

Table S5. Linear Regression of age-standardised YLD rate and Healthcare Access and Quality (HAQi), model results

	**Estimate**	**Standard Error**	**t statistic**	**p-value**
**Intercept**	**742.8336**	**13.8981**	**53.45**	**<2E-16**
**HAQi**	**-0.41934**	0.**2175**	**-19.28**	**<2E-16**

Table S6. Age-standardised prevalence (per 1) and cases of hearing loss, both sexes, projected from 2020 to 2050, by severity and WHO region

		All hearing loss		Moderate to complete hearing loss (35 dB+)	
Region	Year	Age-standardised rate (%) (95% UI)	Cases (in millions) (95% UI)	Age-standardised rate (%) (95% UI)	Cases (in millions) (95% UI)
African Region	2020	19·3 (18·6–20·0)	138·8 (132·3–145·3)	6·4 (5·8–7·1)	41·9 (36·7–47·5)
Eastern Mediterranean Region	2020	15·6 (14·8–16·4)	86·6 (82·3–91·3)	4·6 (4·1–5·2)	23·0 (19·9–26·1)
European Region	2020	14·1 (13·4–14·8)	195·4 (184·4–206·8)	3·6 (3·1–4·1)	53·6 (46·6–60·7)
Region of the Americas	2020	18·1 (17·2–19·1)	220·3 (208·6–232·2)	4·4 (3·8–4·9)	54·6 (47·9–61·5)
South-East Asia Region	2020	20·6 (19·9–21·6)	397·9 (382·1–415·9)	6·1 (5·4–6·8)	108·3 (95·5–121·1)
Western Pacific Region	2020	22·0 (21·0–23·0)	551·9 (526·8–576·2)	5·5 (4·9–6·1)	138.8 (123·3–155·9)
African Region	2030	19.0 (18·3–19·7)	190·4 (181·8–199·0)	6·1 (5·5–6·8)	55·9 (49·0–63·0)
Eastern Mediterranean Region	2030	15·3 (14·6–16·2)	116·3 (110·6–122·9)	4·4 (3·9–5·0)	30·2 (26·2–34·3)
European Region	2030	13·9 (13·2–14·6)	215·2 (202·8–228·5)	3·5 (3·0–3·9)	60·4 (52·3–68·5)
Region of the Americas	2030	17·9 (17·0–18·8)	260·3 (246·5–274·2)	4·2 (3·7–4·7)	66·3 (57·9–74·7)
South-East Asia Region	2030	20·3 (19·5–21·2)	480·3 (460·4–502·6)	5·8 (5·1–6·4)	129·6 (114·3–144·9)
Western Pacific Region	2030	22·0 (21·0–23·1)	640·8 (609·5–671·3)	5·3 (4·8–6·0)	169·9 (150·0–191·1)
African Region	2040	18·7 (18·0–19·4)	258·1 (247·1–269·2)	5·9 (5·3–6·5)	74·5 (65·6–83·4)
Eastern Mediterranean Region	2040	15·1 (14·3–16·0)	151.6 (143·6–160·5)	4·2 (3·6–4·7)	39·6 (34·4–45·2)
European Region	2040	13·7 (13·0–14·4)	230·7 (217·0–245·6)	3·3 (2·9–3·8)	67·1 (58·1–75·8)
Region of the Americas	2040	17·7 (16·8–18·7)	296·5 (281·2–312·0)	4·0 (3·5–4·5)	77·8 (68·0–87·3)
South-East Asia Region	2040	20·0 (19·1–21·0)	564·3 (540·3–591·6)	5·5 (4·9–6·1)	153·5 (135·5–172·6)
Western Pacific Region	2040	22·1 (20·9–23·2)	699.8 (662·6–735·3)	5·2 (4·6–5·8)	198·1 (175·5–221·9)
African Region	2050	18·4 (17·7–19·1)	341.9 (328·5–355·6)	5·6 (5·0–6·3)	98·1 (86·9–109·1)
Eastern Mediterranean Region	2050	14·8 (14·0–15·7)	188·8 (178·1–201·0)	3·9 (3·4–4·5)	50·4 (43·8–57·6)
European Region	2050	13·5 (12·8–14·2)	237.9 (223·1–253·8)	3·2 (2·8–3·7)	71·0 (61·9–80·3)
Region of the Americas	2050	17·5 (16·6–18·4)	324·7 (308·0–341·6)	3·8 (3·4–4·3)	86·2 (75·3–96·7)
South-East Asia Region	2050	19·7 (18·8–20·7)	641.4 (612·7–673·7)	5·2 (4·6–5·8)	178·2 (157·3–201·0)
Western Pacific Region	2050	22·1 (20·9–23·3)	716.3 (677·7–752·7)	5·0 (4·5–5·6)	214·5 (190·1–239·1)

Table S7. GATHER checklist

Item #	Checklist item	Reported on page #
**Objectives and funding**		
1	Define the indicator(s), populations (including age, sex, and geographic entities), and time period(s) for which estimates were made.	2
2	List the funding sources for the work.	1
**Data Inputs**		
For all data inputs from multiple sources that are synthesized as part of the study:		
3	Describe how the data were identified and how the data were accessed.	2
4	Specify the inclusion and exclusion criteria. Identify all ad-hoc exclusions.	2
5	Provide information on all included data sources and their main characteristics. For each data source used, report reference information or contact name/institution, population represented, data collection method, year(s) of data collection, sex and age range, diagnostic criteria or measurement method, and sample size, as relevant.	5, 8
6	Identify and describe any categories of input data that have potentially important biases (e.g., based on characteristics listed in item 5).	5, 12
For data inputs that contribute to the analysis but were not synthesized as part of the study:		
7	Describe and give sources for any other data inputs.	5
For all data inputs:		
8	Provide all data inputs in a file format from which data can be efficiently extracted (e.g., a spreadsheet rather than a PDF), including all relevant meta-data listed in item 5. For any data inputs that cannot be shared because of ethical or legal reasons, such as third-party ownership, provide a contact name or the name of the institution that retains the right to the data.	8, Appendix p 3
**Data analysis**		
9	Provide a conceptual overview of the data analysis method. A diagram may be helpful.	5-7, Appendix pp. 3-5
10	Provide a detailed description of all steps of the analysis, including mathematical formulae. This description should cover, as relevant, data cleaning, data pre-processing, data adjustments and weighting of data sources, and mathematical or statistical model(s).	3-5, Appendix p 3, Supplemental Figures 4-6
11	Describe how candidate models were evaluated and how the final model(s) were selected.	2
12	Provide the results of an evaluation of model performance, if done, as well as the results of any relevant sensitivity analysis.	Appendix pg. 3
13	Describe methods for calculating uncertainty of the estimates. State which sources of uncertainty were, and were not, accounted for in the uncertainty analysis.	5
14	State how analytic or statistical source code used to generate estimates can be accessed.	Appendix p 3
**Results and Discussion**		
15	Provide published estimates in a file format from which data can be efficiently extracted.	12, Appendix p 3
16	Report a quantitative measure of the uncertainty of the estimates (e.g. uncertainty intervals).	1, 6, 4-7
17	Interpret results in light of existing evidence. If updating a previous set of estimates, describe the reasons for changes in estimates.	7-9
18	Discuss limitations of the estimates. Include a discussion of any modelling assumptions or data limitations that affect interpretation of the estimates.	8-9

Authors' Contributions

Managing the estimation or publications process

Jaimie Steinmetz, Christopher J L Murray, and Theo Vos.

Writing the first draft of the manuscript

Lydia Haile, Kaloyan Kamenov, Jaimie Steinmetz, and Shelly Chadha.

Primary responsibility for this manuscript focused on: applying analytical methods to produce estimates

Lydia Haile, Paul Briant, Aislyn Orji, and Jaimie Steinmetz.

Primary responsibility for this manuscript focused on: seeking, cataloguing, extracting, or cleaning data; designing or coding figures and tables

Lydia Haile, Aislyn Orji, and Jaimie Steinmetz.

Providing data or critical feedback on data sources

Lydia Haile, Jaimie Steinmetz, Amir Abdoli, Mohammad Abdollahi, Haroon Ahmed, Tarik Ahmed Rashid, Hanadi Al Hamad, Fares Alahdab, Fahad Alanezi, Turki Alanzi, Liaqat Ali, Hubert Amu, Jalal Arabloo, Morteza Arab-Zozani, Judie Arulappan, Palash Chandra Banik, Till Bärnighausen, Sadia Bibi, Sonali Chaudhary, Dinh Toi Chu, Rosa Couto, Xiaochen Dai, Lalit Dandona, Rakhi Dandona, Mandira Lamichhane Dhimal, Meghnath Dhimal , Kerrie Doyle, Michael Ekholuenetale, Irina Filip, Richard Franklin, Shilpa Gaidhane, Syed Amir Gilani, Mahaveer Golechha, Bárbara Goulart, Veer Bala Gupta, Vivek Gupta, Howard J Hoffman, Mehdi Hosseinzadeh, Mowafa Househ, Segun Emmanuel Ibitoye, Sheikh Mohammed Shariful Islam, Sathish Kumar Jayapal, Jost B Jonas, Nawzad Kameran Al-Salihi, Gbenga Kayode, Rovshan Khalilov, Mahalaqua Nazli Khatib, Soewarta Kosen, G Anil Kumar, Huong Lan Nguyen, Savita Lasrado, Stephen Lim, Xuefeng Liu, Stany Lobo, Alaa Makki, Walter Mendoza, Kebadnew Mihretie, Ted R Miller, Mokhtar Mohammadi, Abdollah Mohammadian-Hafshejani, Ali H Mokdad, Seyed Sina Naghibi Irvani, Jean Jacques Noubiap, Bogdan Oancea, Andrew T Olagunju, Bolajoko Olusanya, Jacob Olusanya, Hans Orru, Mayowa Owolabi, Dr Jagadish Rao Padubidri, Keyvan Pakshir, Fatemeh Pashazadeh Kan, Maja Pasovic, Shrikant Pawar, Hai Quang Pham, Amir Radfar, Fakher Rahim, Vafa Rahimi-Movaghar, Mohammad Hifz Ur Rahman, Amir Masoud Rahmani, Chythra R Rao, Sowmya J Rao, Vahid Rashedi, David L Rawaf, Salman Rawaf, Andre Renzaho, Violet Rodrigues, Dr Umar Saeed, Abdallah M Samy, Milena Santric Milicevic, Brijesh Sathian, Maheswar Satpathy, Monika Sawhney, Allen Seylani, Masood Ali Shaikh, Mohd Shannawaz, Wondimeneh Shiferaw, Soraya Siabani, Jasvinder Singh, Jitendra Kumar Singh, Deepika Singhal, Valentin Skryabin, Anna Skryabina, Houman Sotoudeh, Animut Tagele Tamiru, Giang Thu Vu, Marcos Roberto Tovani-Palone, Bach Tran Xuan, Saif Ullah, Vasiliy Vlassov, Bay Vo, Ali Yadollahpour, Birhanu Wubale Yirdaw, Naohiro Yonemoto, Chuanhua Yu, Ismaeel Yunusa, Zhi-Jiang Zhang, Christopher J L Murray, Adrian Davis, Theo Vos, and Shelly Chadha.

Developing methods or computational machinery

Lydia Haile, Paul Briant, Aislyn Orji, Jaimie Steinmetz, Tarik Ahmed Rashid, Liaqat Ali, Hubert Amu, Palash Chandra Banik, Sonali Chaudhary, Shilpa Gaidhane, Farhad Ghamari, Ahmad Ghashghaee, Howard J Hoffman, Mehdi Hosseinzadeh, Mowafa Househ, Nawzad Kameran Al-Salihi, Rovshan Khalilov, Mahalaqua Nazli Khatib, Alaa Makki, Mokhtar Mohammadi, Ali H Mokdad, Mohammad Ali Moni, Amir Masoud Rahmani, Abdallah M Samy, Maheswar Satpathy, Deepika Singhal, Bay Vo, Mikhail Zastrozhin, Anastasiia Zastrozhina, Christopher J L Murray, and Theo Vos.

Providing critical feedback on methods or results

Lydia Haile, Paul Briant, Aislyn Orji, Jaimie Steinmetz, Mohammad Abdollahi, Eman Abu-Gharbieh, Haroon Ahmed, Tarik Ahmed Rashid, Yonas Akalu, Hanadi Al Hamad, Fares Alahdab, Fahad Alanezi, Turki Alanzi, Liaqat Ali, Vahid Alipour, Rajaa Al-Raddadi, Hubert Amu, Jalal Arabloo, Morteza Arab-Zozani, Judie Arulappan, Zaheer-Ud-Din Babar, Atif Baig, Palash Chandra Banik, Till Bärnighausen, Amadou Barrow, Rose Bender, Akshaya Bhagavathula, Nikha Bhardwaj, Pankaj Bhardwaj, Sadia Bibi, Ali Bijani, Katrin Burkart, Christopher Cederroth, Jaykaran Charan, Sonali Chaudhary, Sonali Choudhari, Dinh Toi Chu, Rosa Couto, Amare Dagnew, Baye Dagnew, Saad Dahlawi, Xiaochen Dai, Lalit Dandona, Rakhi Dandona, Desta Debalkie Atnafu, Assefa Desalew, Deepak Dhamnetiya, Mandira Lamichhane Dhimal, Meghnath Dhimal , Kerrie Doyle, Michael Ekholuenetale, Irina Filip, Florian Fischer, Richard Franklin, Abhay Gaidhane, Shilpa Gaidhane, Ahmad Ghashghaee, Ghozali Ghozali, Ionela-Roxana Glăvan, Mahaveer Golechha, Bárbara Goulart, Veer Bala Gupta, Vivek Gupta, Samer Hamidi, Billy Hammond, Khezar Hayat, Golnaz Heidari, Howard J Hoffman, Kathleen Hopf, Mehdi Hosseinzadeh, Mowafa Househ, Rabia Hussain, Bing-Fang Hwang, Segun Emmanuel Ibitoye, Olayinka Ilesanmi, Sheikh Mohammed Shariful Islam, Masao Iwagami, Louis Jacob, Sathish Kumar Jayapal, Ravi Prakash Jha, Jost B Jonas, Rohollah Kalhor, Nawzad Kameran Al-Salihi, Himal Kandel, Ayele Semachew Kasa, Gbenga Kayode,

Rovshan Khalilov, Ejaz Khan, Mahalaqua Nazli Khatib, Ai Koyanagi, G Anil Kumar, Huong Lan Nguyen, Savita Lasrado, Xuefeng Liu, Stany Lobo, Alessandra Lugo, Alaa Makki, Walter Mendoza, Amanual Mersha, Ted R Miller, Sanjeev Misra, Teroj Mohamed, Mokhtar Mohammadi, Abdollah Mohammadian-Hafshejani, Arif Mohammed, Ali H Mokdad, Mohammad Ali Moni, Seyed Sina Naghibi Irvani, Sandhya Neupane Kandel, Molly Nixon, Jean Jacques Noubiap, Bogdan Oancea, Victor Oguoma, Andrew T Olagunju, Bolajoko Olusanya, Jacob Olusanya, Hans Orru, Mayowa Owolabi, Dr Jagadish Rao Padubidri, Shahina Pardhan, Maja Pasovic, Shrikant Pawar, Akram Pourshams, Hai Quang Pham, Mohammad Rabiee, Navid Rabiee, Amir Radfar, Fakher Rahim, Vafa Rahimi-Movaghar, Mohammad Hifz Ur Rahman, Mosiur Rahman, Amir Masoud Rahmani, Juwel Rana, Chythra R Rao, Sowmya J Rao, Vahid Rashedi, David L Rawaf, Salman Rawaf, Andre Renzaho, Aziz Rezapour, Rezaul Karim Ripon, Violet Rodrigues, Neeti Rustagi, Dr Umar Saeed, Abdallah M Samy, Milena Santric Milicevic, Brijesh Sathian, Maheswar Satpathy, Monika Sawhney, Allen Seylani, Masood Ali Shaikh, Mohd Shannawaz, Wondimeneh Shiferaw, Soraya Siabani, Anjali Singal, Jasvinder Singh, Jitendra Kumar Singh, Deepika Singhal, Valentin Skryabin, Anna Skryabina, Houman Sotoudeh, Animut Tagele Tamiru, Md Tareque, Rekha Thapar, Giang Thu Vu, Marcos Roberto Tovani-Palone, Bach Tran Xuan, Saif Ullah, Francesco S Violante, Bay Vo, Avina Vongpradith, Jingkai Wei, Biruk Wogayehu, Ali Yadollahpour, Yigizie Yeshaw, Vahit Yiğit, Naohiro Yonemoto, Chuanhua Yu, Ismaeel Yunusa, Mohammad Zamani, Mikhail Zastrozhin, Anastasiia Zastrozhina, Jeff Zhao, Christopher J L Murray, Adrian Davis, Theo Vos, and Shelly Chadha.

Drafting the work or revising is critically for important intellectual content

Lydia Haile, Kaloyan Kamenov, Paul Briant, Aislyn Orji, Jaimie Steinmetz, Eman Abu-Gharbieh, Haroon Ahmed, Yonas Akalu, Fares Alahdab, Liaqat Ali, Jalal Arabloo, Morteza Arab-Zozani, Atif Baig, Till Bärnighausen, Amadou Barrow, Rose Bender, Akshaya Bhagavathula, Christopher Cederroth, Dinh Toi Chu, Rosa Couto, Baye Dagnew, Deepak Dhamnetiya, Mandira Lamichhane Dhimal, Meghnath Dhimal , Bruce Duncan, Irina Filip, Florian Fischer, Silvano Gallus, Farhad Ghamari, Mahaveer Golechha, Bárbara Goulart, Veer Bala Gupta, Vivek Gupta, Simon I Hay, Golnaz Heidari, Ivo Iavicoli, Segun Emmanuel Ibitoye, Olayinka Ilesanmi, Sheikh Mohammed Shariful Islam, Louis Jacob, Sathish Kumar Jayapal, Ravi Prakash Jha, Jost B Jonas, Himal Kandel, Ayele Semachew Kasa, Gbenga Kayode, Rovshan Khalilov, Ejaz Khan, Ai Koyanagi, Huong Lan Nguyen, Iván Landires, Savita Lasrado, Walter Mendoza, Ted R Miller, Teroj Mohamed, Abdollah Mohammadian-Hafshejani, Arif Mohammed, Ali H Mokdad, Mohammad Ali Moni, Seyed Sina Naghibi Irvani, Sandhya Neupane Kandel, Molly Nixon, Jean Jacques Noubiap, Virginia Nuñez-Samudio, Victor Oguoma, Andrew T Olagunju, Bolajoko Olusanya, Jacob Olusanya, Hans Orru, Mayowa Owolabi, Dr Jagadish Rao Padubidri, Shahina Pardhan, Shrikant Pawar, Marina Pinheiro, Hai Quang Pham, Amir Radfar, Fakher Rahim, Vafa Rahimi-Movaghar, Mohammad Hifz Ur Rahman, Chythra R Rao, Sowmya J Rao, David L Rawaf, Salman Rawaf, Andre Renzaho, Rezaul Karim Ripon, Violet Rodrigues, Amirhossein Sahebkar, Abdallah M Samy, Milena Santric Milicevic, Maheswar Satpathy, Winfried Schlee, Maria Schmidt, Allen Seylani, Mohd Shannawaz, Anjali Singal, Jasvinder Singh, Valentin Skryabin, Anna Skryabina, Animut Tagele Tamiru, Giang Thu Vu, Marcos Roberto Tovani-Palone, Bach Tran Xuan, Sahel Valadan Tahbaz, Vasiliy Vlassov, Hossein Yahyazadeh, Yigizie Yeshaw, Vahit Yiğit, Naohiro Yonemoto, Mikhail Zastrozhin, Anastasiia Zastrozhina, Zhi-Jiang Zhang, Christopher J L Murray, Adrian Davis, and Shelly Chadha.

Extracting, cleaning, or cataloging data; designing or coding figures and tables

Lydia Haile, Jaimie Steinmetz, Liaqat Ali, Sonali Chaudhary, Shilpa Gaidhane, Rovshan Khalilov, Mahalaqua Nazli Khatib, Ali H Mokdad, Shrikant Pawar, Abdallah M Samy, Maheswar Satpathy, Deepika Singhal, Emma Spurlock, Avina Vongpradith, and Vahit Yiğit.

Managing the overall research enterprise

Jaimie Steinmetz, Ashkan Afshin, Charlie Ashbaugh, Lalit Dandona, Simon I Hay, Ali H Mokdad, Molly Nixon, Christopher J L Murray, and Theo Vos.

Region	Hearing loss		Mild hearing loss		Moderate hearing loss		Moderately severe hearing loss		Severe hearing loss		Profound hearing loss		Complete hearing loss	
	Age-standardised prevalence rate (%) (95% UI)	Cases (in thousands) (95% UI)	Age-standardised prevalence rate (%) (95% UI)	Cases (in thousands) (95% UI)	Age-standardised prevalence rate (%) (95% UI)	Cases (in thousands) (95% UI)	Age-standardised prevalence rate (%) (95% UI)	Cases (in thousands) (95% UI)	Age-standardised prevalence rate (%) (95% UI)	Cases (in thousands) (95% UI)	Age-standardised prevalence rate (%) (95% UI)	Cases (in thousands) (95% UI)	Age-standardised prevalence rate (%) (95% UI)	Cases (in thousands) (95% UI)
Afghanistan	13·1 (12·5–13·8)	2480 (2340–2620)	8·04 (7·59–8·48)	1670 (1570–1760)	3·25 (2·82–3·67)	507 (436–580)	1·23 (1·03–1·48)	167 (138–202)	0·269 (0·212–0·331)	47 (36·7–59·1)	0·197 (0·158–0·244)	51·7 (38·7–67·2)	0·155 (0·126–0·19)	38·8 (29·1–50·9)
Albania	18 (17·2–18·8)	672 (641–703)	13·7 (13·1–14·4)	506 (480–532)	2·81 (2·46–3·18)	111 (96·5–127)	0·95 (0·784–1·15)	37·7 (30·5–46·5)	0·179 (0·136–0·225)	6·69 (4·94–8·61)	0·186 (0·146–0·234)	5·99 (4·71–7·51)	0·122 (0·0987–0·15)	4·22 (3·41–5·17)
Algeria	12·6 (12–13·2)	4610 (4390–4840)	8·24 (7·81–8·69)	3180 (3010–3350)	2·84 (2·47–3·23)	945 (816–1080)	1·05 (0·86–1·27)	320 (262–391)	0·209 (0·161–0·263)	67·3 (51·8–85·5)	0·166 (0·131–0·206)	60·8 (47·7–76·5)	0·104 (0·0842–0·128)	37·6 (30·1–47·1)
American Samoa	21·2 (20·5–21·9)	10·8 (10·5–11·2)	16·7 (16–17·3)	8·68 (8·33–9·02)	2·91 (2·54–3·28)	1·39 (1·2–1·58)	0·972 (0·81–1·17)	0·438 (0·363–0·53)	0·234 (0·182–0·292)	0·111 (0·0863–0·14)	0·25 (0·197–0·315)	0·133 (0·104–0·169)	0·159 (0·127–0·196)	0·0811 (0·0645–0·102)
Andorra	11 (10·5–11·6)	14 (13·3–14·8)	8·65 (8·2–9·12)	10·7 (10·2–11·4)	1·79 (1·54–2·08)	2·43 (2·1–2·84)	0·424 (0·314–0·542)	0·599 (0·446–0·769)	0·032 (0·0147–0·0526)	0·0448 (0·0199–0·0743)	0·0716 (0·056–0·0903)	0·079 (0·0621–0·0997)	0·0764 (0·061–0·0951)	0·0924 (0·0733–0·114)
Angola	18·6 (17·8–19·3)	3320 (3150–3480)	13·9 (13·3–14·6)	2550 (2430–2680)	2·59 (2·24–2·94)	429 (358–506)	1·23 (1·03–1·45)	178 (145–217)	0·464 (0·379–0·563)	83·1 (64·2–105)	0·174 (0·137–0·219)	42·7 (31·1–56·5)	0·161 (0·131–0·197)	34·4 (25·7–44·8)
Antigua and Barbuda	21·8 (20·7–23·1)	22·4 (21·2–23·7)	17·5 (16·4–18·5)	18·2 (17·1–19·3)	2·89 (2·48–3·34)	2·85 (2·44–3·31)	1 (0·813–1·22)	0·935 (0·748–1·15)	0·199 (0·158–0·248)	0·183 (0·144–0·233)	0·184 (0·146–0·23)	0·168 (0·132–0·212)	0·0725 (0·0586–0·0892)	0·0681 (0·0549–0·0843)
Argentina	13·1 (12·5–13·8)	6770 (6420–7130)	9·15 (8·67–9·63)	4650 (4400–4900)	2·74 (2·38–3·11)	1470 (1270–1680)	0·791 (0·643–0·969)	428 (344–526)	0·144 (0·106–0·189)	76 (55·4–101)	0·136 (0·107–0·17)	67·9 (53·5–84·9)	0·154 (0·125–0·188)	78·1 (63·3–95·7)
Armenia	17·9 (17·2–18·7)	685 (655–716)	13·7 (13·1–14·4)	523 (497–551)	2·76 (2·39–3·14)	108 (92·7–123)	0·962 (0·791–1·16)	37·2 (30·2–45·6)	0·191 (0·146–0·239)	7·05 (5·32–8·93)	0·183 (0·143–0·23)	6·14 (4·83–7·68)	0·118 (0·0953–0·146)	4·2 (3·41–5·17)
Australia	13·5 (12·8–14·3)	4960 (4660–5260)	9·78 (9·22–10·3)	3420 (3200–3640)	2·66 (2·33–3·04)	1100 (954–1250)	0·706 (0·565–0·874)	299 (236–371)	0·115 (0·0717–0·164)	46·6 (27·2–66·3)	0·137 (0·109–0·174)	47·3 (37–60)	0·131 (0·106–0·163)	47·6 (37·9–59)
Austria	10·9 (10·4–11·5)	1620 (1520–1720)	8·58 (8·16–9·03)	1200 (1140–1270)	1·81 (1·57–2·09)	326 (279–378)	0·371 (0·27–0·478)	70·8 (50–92·8)	0·0206 (0·00958–0·0365)	3·84 (1·65–7·26)	0·0731 (0·0572–0·0918)	9·8 (7·62–12·5)	0·081 (0·065–0·1)	11·9 (9·42–14·8)
Azerbaijan	18 (17·2–18·7)	1850 (1770–1930)	13·7 (13–14·4)	1480 (1400–1550)	2·78 (2·42–3·17)	249 (212–285)	0·968 (0·798–1·17)	76·6 (62·1–93·8)	0·193 (0·148–0·246)	16·1 (12·3–20·8)	0·184 (0·144–0·23)	17·4 (13·4–22·2)	0·12 (0·0971–0·149)	11·2 (8·85–14)
Bahrain	12·6 (12–13·3)	168 (159–178)	8·41 (7·95–8·86)	127 (119–134)	2·77 (2·42–3·15)	28·4 (24·1–33·4)	1·01 (0·829–1·22)	8·28 (6·7–10·2)	0·193 (0·147–0·243)	1·8 (1·31–2·4)	0·159 (0·126–0·197)	1·89 (1·46–2·41)	0·0942 (0·0769–0·116)	1·16 (0·902–1·49)
Bangladesh	20·1 (19·4–21)	29000 (27900–30200)	15·2 (14·5–15·9)	22300 (21200–23300)	3·23 (2·8–3·68)	4420 (3820–5050)	1·13 (0·936–1·37)	1460 (1200–1760)	0·205 (0·162–0·257)	272 (212–341)	0·267 (0·21–0·335)	397 (308–502)	0·137 (0·111–0·17)	196 (157–245)
Barbados	21·8 (20·7–23)	93·1 (87·7–98·7)	17·5 (16·4–18·6)	73·5 (68·6–78·7)	2·89 (2·49–3·3)	13·3 (11·4–15·3)	0·965 (0·788–1·19)	4·55 (3·65–5·64)	0·183 (0·143–0·23)	0·81 (0·621–1·05)	0·183 (0·145–0·228)	0·691 (0·54–0·868)	0·0723 (0·0585–0·0889)	0·307 (0·245–0·38)
Belarus	17·9 (17·1–18·7)	2460 (2360–2580)	13·7 (13·1–14·4)	1860 (1760–1950)	2·68 (2·34–3·05)	392 (339–450)	0·99 (0·817–1·2)	149 (121–182)	0·212 (0·167–0·264)	29·7 (23–37·8)	0·178 (0·139–0·221)	20·7 (16·2–25·8)	0·112 (0·091–0·139)	14·4 (11·7–17·6)
Belgium	11·1 (10·5–11·6)	2070 (1950–2190)	8·55 (8·12–9)	1490 (1410–1580)	1·79 (1·55–2·08)	407 (349–473)	0·517 (0·407–0·643)	127 (98·2–161)	0·0617 (0·0408–0·0853)	14·1 (8·61–20·2)	0·0729 (0·0572–0·0924)	12·6 (9·88–16)	0·0807 (0·065–0·0999)	15·2 (12–19)
Belize	22·1 (20·9–23·3)	72·7 (69·1–76·5)	17·4 (16·4–18·4)	58·9 (55·5–62·3)	3·11 (2·7–3·56)	9·17 (7·9–10·5)	1·08 (0·876–1·32)	2·92 (2·4–3·54)	0·22 (0·175–0·273)	0·652 (0·52–0·809)	0·2 (0·159–0·25)	0·715 (0·555–0·902)	0·086 (0·0701–0·106)	0·274 (0·221–0·343)
Benin	18·9 (18·1–19·6)	1450 (1370–1530)	13·7 (13·1–14·3)	1060 (1000–1110)	2·96 (2·56–3·35)	225 (189–265)	1·3 (1·09–1·55)	88 (71·9–108)	0·465 (0·379–0·567)	38·1 (29·5–48·8)	0·235 (0·185–0·298)	25·8 (18·9–34·3)	0·195 (0·156–0·242)	18·7 (13·9–24·6)
Bermuda	21·8 (20·6–22·9)	22·9 (21·5–24·3)	17·6 (16·6–18·7)	17·9 (16·7–19·2)	2·76 (2·38–3·19)	3·29 (2·79–3·81)	0·955 (0·774–1·17)	1·22 (0·966–1·5)	0·187 (0·147–0·236)	0·219 (0·169–0·28)	0·175 (0·138–0·217)	0·161 (0·125–0·204)	0·0657 (0·0529–0·0807)	0·0726 (0·0573–0·0903)
Bhutan	20·3 (19·5–21·1)	133 (128–139)	15·2 (14·5–15·9)	102 (97·3–107)	3·26 (2·83–3·71)	19·9 (17·3–22·7)	1·25 (1·05–1·5)	7·16 (5·95–8·66)	0·212 (0·167–0·263)	1·26 (0·969–1·57)	0·237 (0·188–0·296)	1·62 (1·27–2·06)	0·138 (0·112–0·17)	0·908 (0·725–1·13)
Bolivia	22·1 (20·9–23·4)	2200 (2080–2320)	18 (16·9–19·1)	1830 (1720–1940)	2·28 (1·95–2·64)	207 (177–242)	1·28 (1·05–1·54)	109 (88·8–132)	0·26 (0·206–0·323)	23·3 (18·4–29)	0·199 (0·156–0·248)	20·8 (16–25·8)	0·105 (0·0853–0·129)	10 (8·12–12·4)
Bosnia and Herzegovina	17·9 (17·2–18·7)	900 (859–942)	13·8 (13·1–14·4)	682 (646–716)	2·75 (2·41–3·13)	148 (126–170)	0·925 (0·77–1·12)	49·3 (39·6–60·9)	0·171 (0·129–0·216)	8·48 (6·23–11·1)	0·181 (0·143–0·226)	7·44 (5·76–9·41)	0·116 (0·0942–0·143)	5·24 (4·19–6·45)
Botswana	19 (18·3–19·8)	354 (338–370)	14·4 (13·8–15·2)	279 (265–293)	2·45 (2·11–2·77)	40·5 (34·2–46·4)	1·33 (1·12–1·59)	20·2 (16·6–24·1)	0·411 (0·33–0·501)	6·61 (5·21–8·26)	0·217 (0·171–0·271)	4·31 (3·28–5·51)	0·179 (0·146–0·221)	3·28 (2·55–4·15)
Brazil	19·9 (19·1–20·6)	47200 (45500–49200)	14·8 (14·1–15·5)	35600 (33800–37200)	3·75 (3·27–4·2)	8790 (7640–9920)	0·851 (0·699–1·04)	1940 (1580–2400)	0·182 (0·146–0·227)	406 (324–514)	0·19 (0·15–0·241)	419 (329–528)	0·0567 (0·0457–0·0703)	128 (103–158)
Brunei	13·5 (12·8–14·1)	49 (46·6–51·4)	9·62 (9·12–10·1)	38·2 (36·1–40·4)	2·8 (2·43–3·17)	7·8 (6·68–8·96)	0·669 (0·54–0·818)	1·68 (1·35–2·08)	0·111 (0·0691–0·156)	0·307 (0·197–0·433)	0·137 (0·108–0·172)	0·483 (0·376–0·618)	0·143 (0·116–0·177)	0·491 (0·385–0·627)
Bulgaria	17·8 (17·1–18·6)	2060 (1960–2160)	13·8 (13·2–14·5)	1530 (1450–1610)	2·7 (2·36–3·05)	355 (305–406)	0·902 (0·744–1·09)	123 (98·1–152)	0·163 (0·122–0·206)	20·3 (14·7–26·9)	0·177 (0·139–0·221)	16·8 (13–21·2)	0·11 (0·0892–0·137)	11·9 (9·44–14·8)
Burkina Faso	19·1 (18·4–19·9)	2700 (2560–2840)	13·6 (13–14·2)	1910 (1820–2010)	3·15 (2·73–3·56)	441 (372–521)	1·38 (1·17–1·63)	172 (139–211)	0·505 (0·411–0·613)	76·1 (59·1–97·1)	0·254 (0·199–0·323)	50·7 (36·8–66·7)	0·228 (0·182–0·281)	40·4 (30·3–52·1)
Burundi	19·3 (18·6–20·1)	1400 (1330–1470)	13·7 (13·1–14·3)	1000 (951–1050)	3·34 (2·9–3·78)	243 (204–289)	1·5 (1·26–1·78)	93·9 (75·5–115)	0·403 (0·332–0·488)	28·5 (22·1–35·9)	0·156 (0·123–0·197)	15·4 (11·3–20·4)	0·2 (0·161–0·246)	18·2 (13·5–23·7)
Cambodia	22 (21·3–22·8)	3130 (3020–3250)	16·3 (15·6–16·9)	2390 (2280–2480)	3·64 (3·2–4·09)	470 (408–531)	1·22 (1·02–1·45)	147 (123–178)	0·29 (0·231–0·356)	38·9 (30·2–48·4)	0·338 (0·268–0·426)	53·1 (41·1–68·1)	0·253 (0·203–0·313)	38 (29·5–47·9)
Cameroon	18·6 (17·9–19·3)	3460 (3290–3630)	13·8 (13·2–14·4)	2600 (2470–2730)	2·78 (2·41–3·14)	496 (416–581)	1·22 (1·03–1·44)	192 (157–234)	0·426 (0·345–0·514)	79·9 (62·3–100)	0·217 (0·171–0·273)	55·1 (40·5–72)	0·166 (0·134–0·205)	36·5 (27·8–47·1)
Canada	12 (11·5–12·4)	7000 (6740–7260)	8·52 (8·13–8·91)	4700 (4490–4920)	2·37 (2·08–2·67)	1590 (1390–1800)	0·668 (0·552–0·807)	459 (374–558)	0·143 (0·103–0·186)	89·9 (61·3–121)	0·132 (0·105–0·166)	73·1 (57·1–92·7)	0·145 (0·118–0·176)	82·6 (66–101)
Cape Verde	18·5 (17·8–19·3)	92·4 (88·7–96·4)	13·8 (13·2–14·5)	69·9 (66·7–73)	2·71 (2·35–3·07)	13 (11·3–14·8)	1·19 (1–1·41)	5·58 (4·68–6·58)	0·413 (0·335–0·497)	2·02 (1·62–2·46)	0·212 (0·168–0·268)	1·14 (0·881–1·45)	0·158 (0·127–0·195)	0·817 (0·642–1·01)
Central African Republic	18·9 (18·2–19·7)	632 (601–663)	13·8 (13·2–14·4)	473 (450–496)	2·85 (2·47–3·27)	88·5 (72·9–104)	1·36 (1·14–1·61)	36·6 (29·4–44·9)	0·526 (0·428–0·637)	17·4 (13·4–22·2)	0·196 (0·155–0·247)	8·7 (6·31–11·6)	0·206 (0·166–0·253)	8·25 (6·11–10·7)
Chad	19·3 (18·6–20)	1780 (1680–1880)	13·7 (13·1–14·3)	1240 (1180–1300)	3·21 (2·78–3·63)	303 (251–359)	1·39 (1·18–1·65)	117 (93·9–143)	0·514 (0·419–0·625)	53·7 (41·2–68·7)	0·257 (0·203–0·323)	36·8 (26·5–48·6)	0·233 (0·185–0·288)	29·1 (21·2–37·9)
Chile	13·1 (12·5–13·8)	2980 (2830–3150)	9·13 (8·63–9·63)	2050 (1940–2170)	2·68 (2·32–3·07)	627 (539–720)	0·851 (0·689–1·04)	199 (159–244)	0·186 (0·145–0·235)	42·1 (32·5–53·8)	0·132 (0·105–0·166)	28·1 (22·1–35·6)	0·145 (0·117–0·177)	31·7 (25·5–39)
China	22·6 (21·7–23·5)	427000 (409000–444000)	17·4 (16·6–18·1)	331000 (315000–346000)	3·35 (2·95–3·78)	63400 (55100–72500)	1·12 (0·939–1·32)	19700 (16200–23900)	0·273 (0·219–0·336)	4540 (3580–5680)	0·275 (0·218–0·341)	4160 (3300–5170)	0·204 (0·167–0·249)	3350 (2720–4090)
Colombia	21·9 (20·7–23)	11400 (10800–12000)	17·4 (16·3–18·4)	9010 (8450–9550)	3·03 (2·63–3·46)	1590 (1380–1810)	0·996 (0·814–1·22)	530 (434–647)	0·181 (0·14–0·227)	94·9 (73·6–119)	0·195 (0·153–0·243)	98·1 (77·8–122)	0·0826 (0·0668–0·102)	43 (34·8–53·2)
Comoros	18·8 (18·1–19·6)	110 (106–115)	13·8 (13·2–14·4)	81·7 (77·9–85·5)	3·05 (2·65–3·42)	17·3 (15–19·8)	1·35 (1·14–1·6)	7·14 (5·97–8·54)	0·356 (0·291–0·431)	1·95 (1·56–2·41)	0·14 (0·111–0·176)	0·897 (0·692–1·15)	0·159 (0·13–0·194)	0·975 (0·768–1·22)

Congo (Brazzaville)	18·4 (17·7–19·1)	686 (655–719)	14·1 (13·5–14·7)	544 (519–572)	2·44 (2·1–2·76)	81·1 (68·6–94·1)	1·16 (0·977–1·37)	34·2 (28·3–41·1)	0·425 (0·345–0·517)	14·5 (11·5–18·3)	0·161 (0·126–0·203)	7·05 (5·26–9·2)	0·137 (0·111–0·168)	5·41 (4·13–6·95)
Cook Islands	21 (20·3–21·8)	4·6 (4·44–4·76)	16·7 (16–17·3)	3·61 (3·47–3·75)	2·81 (2·45–3·18)	0·652 (0·567–0·744)	0·933 (0·776–1·13)	0·212 (0·173–0·261)	0·218 (0·169–0·273)	0·0468 (0·0362–0·0592)	0·239 (0·189–0·301)	0·0462 (0·0366–0·0579)	0·145 (0·117–0·179)	0·0297 (0·0241–0·0367)
Costa Rica	21·8 (20·7–22·9)	1120 (1060–1180)	17·4 (16·4–18·5)	899 (846–956)	2·96 (2·54–3·39)	150 (129–173)	0·967 (0·787–1·18)	48·7 (39·7–59·3)	0·172 (0·132–0·217)	8·61 (6·62–10·9)	0·189 (0·15–0·236)	9·26 (7·34–11·5)	0·0775 (0·0627–0·0951)	3·9 (3·17–4·78)
Croatia	17·8 (17·1–18·6)	1230 (1170–1290)	13·8 (13·2–14·5)	916 (870–965)	2·67 (2·33–3·03)	212 (183–244)	0·883 (0·727–1·07)	74·1 (59·1–91·6)	0·156 (0·117–0·2)	12 (8·61–16)	0·174 (0·135–0·217)	10·2 (7·95–12·9)	0·106 (0·0864–0·131)	7·05 (5·64–8·71)
Cuba	22 (20·8–23·2)	3630 (3430–3850)	17·4 (16·4–18·5)	2820 (2640–3010)	2·99 (2·58–3·44)	531 (456–613)	1·06 (0·863–1·29)	199 (160–242)	0·22 (0·176–0·274)	38·4 (30·1–48·3)	0·191 (0·151–0·239)	27·8 (21·7–35·1)	0·0787 (0·0641–0·0972)	13·1 (10·6–16·2)
Cyprus	11 (10·4–11·6)	196 (185–207)	8·56 (8·14–9)	151 (143–159)	1·82 (1·56–2·11)	33·8 (28·6–40)	0·444 (0·335–0·562)	7·89 (5·68–10·3)	0·0365 (0·0175–0·0583)	0·616 (0·27–1·04)	0·0732 (0·0576–0·0917)	1·16 (0·9–1·47)	0·0814 (0·0657–0·101)	1·34 (1·07–1·66)
Czech Republic	17·7 (17–18·5)	2990 (2850–3130)	13·9 (13·2–14·5)	2260 (2140–2370)	2·62 (2·29–2·96)	498 (428–567)	0·85 (0·704–1·02)	168 (135–207)	0·144 (0·104–0·185)	26·3 (18·3–35·3)	0·169 (0·133–0·212)	24 (18·9–30·3)	0·101 (0·0814–0·125)	16·3 (13–20·2)
Côte d'Ivoire	18·9 (18·2–19·6)	3210 (3050–3370)	13·8 (13·2–14·4)	2400 (2280–2520)	2·91 (2·52–3·31)	469 (395–551)	1·27 (1·07–1·5)	180 (146–221)	0·452 (0·368–0·55)	76·8 (59·3–97·7)	0·229 (0·181–0·288)	51·8 (37·9–67·9)	0·184 (0·147–0·227)	36·7 (27·5–47·2)
DR Congo	18·8 (18·1–19·5)	10300 (9770–10800)	13·9 (13·3–14·5)	7760 (7390–8140)	2·71 (2·35–3·09)	1400 (1160–1640)	1·29 (1·09–1·52)	589 (479–711)	0·493 (0·401–0·598)	271 (211–343)	0·184 (0·145–0·233)	136 (100–178)	0·182 (0·147–0·224)	119 (89·1–152)
Denmark	10·8 (10·2–11·4)	1030 (964–1090)	8·64 (8·22–9·09)	781 (736–826)	1·74 (1·49–2·01)	201 (170–234)	0·253 (0·186–0·347)	29·4 (21·2–41·5)	0·00936 (0·00642–0·0156)	1·06 (0·671–1·85)	0·071 (0·0557–0·0895)	6·09 (4·72–7·82)	0·0765 (0·061–0·0951)	7·12 (5·56–8·97)
Djibouti	18·9 (18·2–19·6)	167 (159–175)	13·9 (13·2–14·5)	127 (121–134)	3 (2·59–3·39)	24·6 (20·7–28·6)	1·35 (1·13–1·6)	9·53 (7·78–11·7)	0·354 (0·288–0·427)	2·69 (2·12–3·39)	0·139 (0·109–0·175)	1·37 (1·04–1·78)	0·155 (0·125–0·189)	1·44 (1·09–1·82)
Dominica	21·9 (20·7–23·2)	18·3 (17·2–19·4)	17·5 (16·5–18·6)	14·4 (13·5–15·4)	2·93 (2·52–3·38)	2·55 (2·18–2·96)	1·01 (0·819–1·23)	0·903 (0·723–1·11)	0·202 (0·158–0·251)	0·173 (0·135–0·219)	0·185 (0·147–0·232)	0·145 (0·115–0·182)	0·0739 (0·0601–0·0916)	0·0618 (0·0498–0·0765)
Dominican Republic	22·1 (21–23·2)	2200 (2090–2320)	17·4 (16·4–18·4)	1760 (1660–1860)	3·11 (2·68–3·57)	294 (254–339)	1·08 (0·876–1·31)	97·8 (79·6–119)	0·22 (0·173–0·273)	20·6 (16·3–25·4)	0·2 (0·158–0·25)	20·3 (15·9–25·5)	0·0861 (0·0705–0·106)	8·34 (6·8–10·3)
Ecuador	22·2 (21–23·5)	3580 (3400–3780)	17·9 (16·8–19)	2920 (2740–3100)	2·55 (2·17–2·96)	392 (335–457)	1·15 (0·941–1·41)	170 (139–208)	0·286 (0·225–0·359)	43·5 (34·2–54·3)	0·228 (0·18–0·281)	37·5 (29·5–46·5)	0·129 (0·105–0·159)	20·4 (16·5–25·2)
Egypt	12·7 (12–13·3)	9240 (8780–9710)	8·25 (7·8–8·7)	6500 (6150–6850)	2·87 (2·5–3·26)	1820 (1560–2090)	1·06 (0·873–1·27)	587 (476–711)	0·213 (0·163–0·267)	131 (101–164)	0·167 (0·133–0·208)	131 (101–166)	0·106 (0·0863–0·131)	80·8 (63·4–103)
El Salvador	21·9 (20·8–23·1)	1310 (1250–1380)	17·3 (16·2–18·3)	1030 (969–1090)	3·11 (2·69–3·57)	187 (163–214)	1·03 (0·837–1·25)	63·4 (52·1–76·7)	0·19 (0·149–0·239)	11·8 (9·27–14·7)	0·202 (0·159–0·251)	12·6 (9·96–15·7)	0·0883 (0·0715–0·108)	5·49 (4·46–6·73)
Equatorial Guinea	18·2 (17·5–18·9)	152 (145–159)	14·1 (13·5–14·7)	120 (115–127)	2·32 (2–2·64)	17·7 (14·9–21)	1·1 (0·921–1·3)	7·45 (6·02–9·04)	0·397 (0·317–0·483)	3·25 (2·49–4·19)	0·153 (0·12–0·192)	1·79 (1·3–2·38)	0·123 (0·0998–0·151)	1·21 (0·907–1·59)
Eritrea	19 (18·2–19·7)	814 (774–854)	13·7 (13·1–14·3)	603 (572–635)	3·15 (2·73–3·54)	130 (109–153)	1·39 (1·17–1·65)	49·3 (39·9–61)	0·365 (0·3–0·442)	14·4 (11·2–18·4)	0·145 (0·114–0·183)	7·96 (5·84–10·6)	0·172 (0·14–0·21)	8·64 (6·45–11·1)
Estonia	17·8 (17·1–18·6)	369 (352–388)	13·8 (13·1–14·5)	272 (258–287)	2·57 (2·23–2·92)	62·1 (53·5–71·1)	0·943 (0·774–1·14)	25 (20·2–30·7)	0·196 (0·153–0·245)	4·73 (3·62–6·1)	0·169 (0·133–0·212)	3·08 (2·42–3·85)	0·101 (0·0816–0·125)	2·1 (1·67–2·59)
Ethiopia	19·6 (18·8–20·4)	13000 (12300–13800)	13·7 (13·1–14·3)	9090 (8630–9540)	3·53 (3·07–3·98)	2410 (2020–2840)	1·54 (1·3–1·83)	912 (744–1120)	0·417 (0·341–0·504)	279 (219–356)	0·17 (0·134–0·215)	156 (114–209)	0·209 (0·169–0·256)	175 (131–227)
Federated States of Micronesia	21·5 (20·8–22·2)	18·3 (17·6–19)	16·6 (15·9–17·2)	14·7 (14·1–15·3)	3·11 (2·72–3·49)	2·28 (1·96–2·61)	1·05 (0·88–1·27)	0·695 (0·576–0·855)	0·268 (0·209–0·334)	0·203 (0·155–0·261)	0·275 (0·215–0·343)	0·258 (0·194–0·329)	0·192 (0·155–0·239)	0·169 (0·13–0·214)
Fiji	21·3 (20·6–22·1)	179 (173–186)	16·6 (16–17·3)	145 (139–150)	2·99 (2·61–3·38)	22·3 (19·2–25·5)	1·01 (0·843–1·21)	6·79 (5·59–8·24)	0·247 (0·191–0·308)	1·82 (1·39–2·3)	0·259 (0·204–0·327)	2·18 (1·68–2·79)	0·171 (0·138–0·211)	1·4 (1·1–1·75)
Finland	10·1 (9·47–10·8)	1030 (955–1120)	7·49 (7·03–7·97)	708 (656–766)	1·94 (1·66–2·27)	243 (203–285)	0·476 (0·355–0·607)	62·8 (45·8–80·8)	0·038 (0·0178–0·0618)	4·8 (1·96–8)	0·0737 (0·0576–0·0934)	6·9 (5·32–9·04)	0·0685 (0·0546–0·085)	7·14 (5·58–9·08)
France	11·1 (10·5–11·6)	12200 (11500–12900)	8·51 (8·08–8·96)	8640 (8170–9130)	1·8 (1·55–2·09)	2460 (2090–2870)	0·536 (0·422–0·666)	807 (621–1010)	0·0673 (0·0455–0·0913)	93·4 (58–131)	0·0735 (0·0571–0·0929)	75·6 (58·8–96·6)	0·0825 (0·0655–0·102)	93·2 (73·1–117)
Gabon	18·2 (17·5–18·9)	245 (236–256)	14·1 (13·5–14·7)	196 (186–205)	2·33 (2·01–2·65)	28·6 (24·3–32·8)	1·1 (0·926–1·31)	12·3 (10·3–14·8)	0·398 (0·319–0·482)	4·92 (3·89–6·11)	0·153 (0·12–0·193)	2·32 (1·75–3)	0·124 (0·101–0·152)	1·72 (1·34–2·16)
Georgia	17·9 (17·2–18·7)	921 (882–967)	13·7 (13–14·4)	685 (652–721)	2·76 (2·41–3·12)	155 (134–176)	0·96 (0·794–1·16)	57 (46·5–69·7)	0·19 (0·145–0·239)	10·5 (7·94–13·3)	0·182 (0·143–0·228)	8·23 (6·52–10·3)	0·118 (0·0956–0·144)	5·88 (4·76–7·17)
Germany	11 (10·4–11·5)	16700 (15700–17800)	8·59 (8·15–9·05)	12200 (11400–12900)	1·76 (1·52–2·02)	3380 (2870–3920)	0·433 (0·322–0·547)	887 (640–1140)	0·0363 (0·0176–0·0579)	69·3 (29–115)	0·0708 (0·0555–0·0889)	98·2 (76·1–125)	0·0756 (0·061–0·0937)	116 (91·3–146)
Ghana	18·4 (17·7–19·2)	4160 (3970–4360)	13·8 (13·2–14·4)	3190 (3030–3350)	2·66 (2·29–3·01)	567 (479–653)	1·17 (0·983–1·38)	222 (184–269)	0·403 (0·328–0·488)	87·6 (69·2–109)	0·208 (0·162–0·264)	57·8 (43·5–75·3)	0·151 (0·122–0·188)	37·5 (29–47·9)
Greece	11·2 (10·6–11·8)	2100 (1970–2230)	8·48 (8·04–8·93)	1450 (1370–1540)	1·81 (1·55–2·1)	429 (362–501)	0·628 (0·503–0·774)	167 (131–209)	0·0984 (0·0756–0·124)	23·2 (17·2–30·5)	0·0752 (0·0591–0·0945)	13 (10–16·6)	0·0865 (0·0692–0·107)	16·4 (12·9–20·6)
Greenland	13·5 (12·9–14·2)	9·16 (8·68–9·68)	9·24 (8·75–9·75)	6·45 (6·08–6·83)	2·93 (2·56–3·35)	1·9 (1·63–2·21)	0·821 (0·668–1)	0·484 (0·386–0·603)	0·184 (0·133–0·24)	0·113 (0·0818–0·149)	0·156 (0·123–0·195)	0·0967 (0·0743–0·125)	0·184 (0·15–0·227)	0·117 (0·0933–0·148)
Grenada	22 (20·9–23·2)	24·9 (23·6–26·4)	17·4 (16·4–18·5)	20·1 (18·8–21·4)	3·02 (2·62–3·46)	3·3 (2·83–3·82)	1·05 (0·848–1·28)	1·07 (0·853–1·32)	0·211 (0·167–0·261)	0·213 (0·167–0·269)	0·192 (0·152–0·24)	0·201 (0·157–0·252)	0·08 (0·0647–0·0988)	0·0835 (0·0675–0·104)
Guam	21 (20·4–21·7)	38·4 (37·2–39·8)	16·8 (16·1–17·4)	30·7 (29·5–31·9)	2·78 (2·43–3·11)	5·07 (4·4–5·71)	0·915 (0·764–1·1)	1·64 (1·37–1·98)	0·209 (0·161–0·261)	0·37 (0·284–0·463)	0·235 (0·184–0·294)	0·405 (0·32–0·506)	0·139 (0·112–0·17)	0·246 (0·198–0·301)
Guatemala	22 (20·9–23·2)	2910 (2770–3050)	17·2 (16·2–18·3)	2330 (2200–2460)	3·2 (2·77–3·66)	388 (336–446)	1·06 (0·87–1·31)	120 (99–146)	0·199 (0·156–0·25)	24·2 (18·7–30·5)	0·209 (0·165–0·259)	31·9 (24·7–40·7)	0·0943 (0·0767–0·116)	12·6 (10·1–15·8)
Guinea	19 (18·3–19·8)	1530 (1450–1600)	13·7 (13·1–14·3)	1090 (1040–1140)	3·08 (2·66–3·49)	247 (208–289)	1·34 (1·13–1·59)	98 (80·3–119)	0·489 (0·397–0·597)	42·1 (33–52·9)	0·245 (0·192–0·308)	27·6 (20·1–36·2)	0·212 (0·17–0·265)	21·1 (15·9–27·4)
Guinea-Bissau	18·9 (18·2–19·7)	224 (213–236)	13·7 (13·1–14·3)	163 (155–171)	3·02 (2·63–3·42)	34·7 (29·1–41·1)	1·32 (1·12–1·58)	13·4 (10·9–16·5)	0·477 (0·388–0·582)	5·84 (4·52–7·43)	0·242 (0·19–0·306)	4·04 (2·97–5·39)	0·205 (0·166–0·253)	3 (2·25–3·86)
Guyana	22 (20·9–23·2)	153 (145–162)	17·4 (16·4–18·5)	124 (117–132)	3·08 (2·66–3·54)	19·5 (16·7–22·6)	1·07 (0·871–1·3)	6·08 (4·9–7·47)	0·218 (0·172–0·272)	1·3 (1·03–1·65)	0·198 (0·157–0·247)	1·38 (1·07–1·74)	0·0846 (0·0684–0·104)	0·543 (0·435–0·673)
Haiti	22·2 (21·1–23·5)	1990 (1890–2090)	17·1 (16·2–18·2)	1600 (1510–1690)	3·36 (2·91–3·88)	261 (224–301)	1·17 (0·951–1·43)	78·5 (63·8–95·9)	0·245 (0·197–0·301)	19·1 (15·2–23·9)	0·221 (0·175–0·274)	22·5 (17·4–28·7)	0·105 (0·0855–0·129)	9·14 (7·19–11·4)
Honduras	22·1 (21–23·2)	1610 (1530–1690)	17·2 (16·2–18·2)	1290 (1220–1360)	3·27 (2·81–3·72)	215 (185–246)	1·08 (0·877–1·32)	64·6 (53·2–78·7)	0·205 (0·161–0·256)	13·3 (10·4–16·7)	0·213 (0·168–0·267)	17·8 (13·7–22·8)	0·0987 (0·0797–0·122)	7·17 (5·7–9·01)
Hungary	17·8 (17–18·6)	2760 (2630–2890)	13·8 (13·1–14·5)	2060 (1960–2170)	2·66 (2·32–3·02)	466 (401–534)	0·881 (0·729–1·06)	162 (130–200)	0·155 (0·115–0·198)	26·4 (19–35·1)	0·174 (0·137–0·217)	22·6 (17·7–28·5)	0·106 (0·0855–0·133)	15·7 (12·6–19·4)
Iceland	11 (10·4–11·6)	53·4 (50·4–56·6)	8·6 (8·18–9·06)	40·1 (38–42·4)	1·8 (1·54–2·07)	9·93 (8·44–11·5)	0·427 (0·32–0·546)	2·49 (1·87–3·2)	0·0328 (0·015–0·0546)	0·188 (0·0839–0·315)	0·0719 (0·0565–0·0905)	0·327 (0·258–0·414)	0·0782 (0·062–0·0972)	0·38 (0·301–0·471)
India	21·1 (20·4–22)	268000 (257000–279000)	15 (14·3–15·7)	194000 (185000–203000)	4·09 (3·59–4·59)	49800 (43300–56000)	1·4 (1·17–1·65)	15800 (13100–19000)	0·3 (0·243–0·372)	3500 (2800–4380)	0·229 (0·181–0·287)	3000 (2320–3830)	0·133 (0·108–0·164)	1670 (1320–2080)
Indonesia	21·8 (21·1–22·6)	54400 (52400–56500)	16·5 (15·9–17·2)	42800 (41000–44600)	3·4 (2·99–3·8)	7530 (6540–8480)	1·11 (0·935–1·33)	2260 (1860–2730)	0·246 (0·199–0·302)	534 (424–669)	0·311 (0·246–0·388)	764 (593–963)	0·206 (0·166–0·254)	495 (392–619)

Iran	12·8 (12·2–13·4)	10100 (9660–10600)	8·28 (7·87–8·74)	6880 (6530–7260)	2·95 (2·6–3·33)	2140 (1870–2420)	1·06 (0·886–1·27)	727 (605–866)	0·207 (0·164–0·258)	147 (116–183)	0·178 (0·142–0·219)	139 (111–173)	0·109 (0·0883–0·133)	85 (67·9–106)
Iraq	12·6 (11·9–13·2)	3560 (3390–3740)	8·24 (7·8–8·71)	2530 (2390–2670)	2·82 (2·44–3·2)	679 (593–772)	1·04 (0·853–1·26)	222 (184–268)	0·205 (0·158–0·257)	49·9 (38·2–62·3)	0·164 (0·131–0·203)	52·8 (41·1–67·1)	0·102 (0·0827–0·125)	31·3 (24·4–39·4)
Ireland	11 (10·4–11·6)	734 (693–775)	8·58 (8·14–9·03)	559 (530–589)	1·8 (1·55–2·09)	131 (113–153)	0·431 (0·323–0·547)	31·8 (23·3–41)	0·0339 (0·0161–0·0558)	2·41 (1·06–4·07)	0·072 (0·0563–0·0911)	4·37 (3·44–5·61)	0·0788 (0·0631–0·0972)	5·07 (4·03–6·26)
Israel	11·1 (10·5–11·6)	1180 (1120–1250)	8·53 (8·11–8·98)	885 (840–932)	1·83 (1·56–2·11)	212 (181–246)	0·492 (0·38–0·617)	58·9 (45·2–74·1)	0·053 (0·0319–0·0763)	6·19 (3·65–8·96)	0·0747 (0·0581–0·0945)	7·78 (6·13–9·8)	0·085 (0·068–0·105)	9·11 (7·28–11·3)
Italy	11·4 (10·9–12)	12800 (12100–13600)	8·46 (8·03–8·92)	8640 (8160–9160)	1·87 (1·6–2·16)	2600 (2190–3030)	0·777 (0·624–0·951)	1210 (961–1490)	0·124 (0·0984–0·154)	171 (131–217)	0·0813 (0·0642–0·101)	80·8 (62·6–103)	0·111 (0·0895–0·136)	121 (95·8–152)
Jamaica	21·9 (20·8–23·1)	653 (620–689)	17·4 (16·4–18·5)	518 (487–550)	2·99 (2·58–3·44)	89 (77–102)	1·04 (0·837–1·27)	31·7 (26–38·5)	0·208 (0·164–0·259)	6·33 (5·02–7·86)	0·19 (0·151–0·237)	5·6 (4·45–6·96)	0·0776 (0·0628–0·0952)	2·36 (1·92–2·89)
Japan	13·6 (13–14·3)	36200 (34200–38200)	9·64 (9·17–10·2)	22400 (21200–23700)	2·9 (2·54–3·26)	9980 (8780–11200)	0·693 (0·572–0·835)	2630 (2150–3160)	0·104 (0·081–0·133)	372 (282–477)	0·147 (0·117–0·183)	367 (283–469)	0·149 (0·121–0·184)	414 (328–516)
Jordan	12·5 (11·9–13·1)	1020 (967–1070)	8·31 (7·85–8·77)	735 (695–776)	2·74 (2·39–3·11)	187 (162–213)	0·997 (0·816–1·21)	59·7 (48·9–72·3)	0·193 (0·147–0·244)	13·1 (10–16·5)	0·158 (0·126–0·197)	14·2 (10·9–18)	0·094 (0·0765–0·116)	8·03 (6·34–10·1)
Kazakhstan	17·9 (17·2–18·7)	3250 (3110–3390)	13·7 (13·1–14·4)	2560 (2440–2690)	2·74 (2·39–3·09)	456 (394–519)	0·95 (0·788–1·15)	146 (120–179)	0·187 (0·142–0·237)	29·8 (22·6–37·7)	0·181 (0·143–0·226)	31·2 (24·5–39·1)	0·116 (0·094–0·143)	19·7 (15·8–24·4)
Kenya	23·3 (22·3–24·5)	7760 (7330–8200)	16 (15·1–16·9)	5340 (5050–5650)	4·52 (3·95–5·09)	1510 (1280–1770)	1·91 (1·6–2·27)	560 (462–678)	0·502 (0·412–0·608)	163 (129–204)	0·213 (0·169–0·269)	92·3 (68·7–121)	0·234 (0·19–0·285)	91·8 (69·8–117)
Kiribati	21·7 (20·9–22·4)	19·6 (18·8–20·3)	16·4 (15·7–17·1)	15·5 (14·8–16·1)	3·29 (2·89–3·72)	2·51 (2·14–2·87)	1·13 (0·944–1·35)	0·779 (0·643–0·948)	0·3 (0·236–0·37)	0·252 (0·19–0·323)	0·297 (0·234–0·371)	0·318 (0·24–0·41)	0·228 (0·183–0·281)	0·225 (0·172–0·284)
Kuwait	12·4 (11·7–13)	447 (423–472)	8·42 (7·96–8·88)	337 (317–358)	2·62 (2·29–2·97)	73·5 (63·1–84·1)	0·934 (0·764–1·13)	23·3 (19·3–27·9)	0·172 (0·127–0·22)	4·82 (3·59–6·25)	0·149 (0·118–0·185)	5·27 (4·09–6·71)	0·081 (0·0659–0·1)	2·9 (2·25–3·67)
Kyrgyzstan	18·1 (17·4–19)	968 (928–1010)	13·6 (13–14·3)	754 (717–792)	2·92 (2·54–3·32)	140 (120–159)	1·03 (0·862–1·25)	45·7 (38–54·5)	0·215 (0·169–0·268)	10·4 (8·12–13)	0·196 (0·153–0·246)	11·3 (8·68–14·5)	0·137 (0·111–0·169)	7·46 (5·92–9·29)
Laos	22 (21·3–22·8)	1260 (1210–1310)	16·3 (15·6–17)	965 (923–1010)	3·62 (3·16–4·07)	181 (156–205)	1·21 (1·01–1·45)	56·6 (47·3–67·8)	0·287 (0·227–0·351)	15·5 (12–19·4)	0·335 (0·264–0·42)	22·3 (16·8–28·7)	0·249 (0·198–0·307)	15·5 (11·7–19·7)
Latvia	17·8 (17–18·5)	555 (531–582)	13·8 (13·1–14·4)	408 (386–430)	2·59 (2·25–2·96)	94·2 (80·9–108)	0·95 (0·78–1·16)	37·8 (30·2–46·5)	0·198 (0·155–0·248)	7·15 (5·44–9·25)	0·171 (0·134–0·213)	4·61 (3·62–5·76)	0·103 (0·083–0·127)	3·17 (2·55–3·92)
Lebanon	12·4 (11·8–13·1)	652 (620–687)	8·24 (7·79–8·69)	435 (410–459)	2·73 (2·38–3·1)	143 (124–162)	0·996 (0·821–1·21)	51·7 (42·4–63)	0·192 (0·147–0·243)	9·98 (7·58–12·7)	0·158 (0·125–0·198)	8·23 (6·48–10·3)	0·0941 (0·0767–0·116)	4·88 (3·99–6·05)
Lesotho	19·3 (18·6–20·1)	313 (300–328)	14·3 (13·7–15)	239 (228–250)	2·63 (2·29–2·98)	39·5 (33·6–45·5)	1·44 (1·21–1·7)	20 (16·6–23·8)	0·454 (0·368–0·553)	6·69 (5·34–8·36)	0·238 (0·189–0·298)	4·38 (3·34–5·62)	0·216 (0·176–0·265)	3·66 (2·83–4·61)
Liberia	19 (18·3–19·8)	609 (578–640)	13·8 (13·2–14·4)	448 (426–471)	3 (2·59–3·39)	92·1 (77·2–108)	1·3 (1·1–1·55)	35·9 (29·2–43·8)	0·471 (0·382–0·578)	15·2 (11·8–19·3)	0·237 (0·185–0·301)	10 (7·41–13·3)	0·198 (0·158–0·244)	7·47 (5·61–9·71)
Libya	12·4 (11·9–13·1)	730 (696–767)	8·3 (7·84–8·76)	519 (490–548)	2·72 (2·37–3·09)	140 (122–159)	0·99 (0·815–1·19)	47·6 (39·4–57·3)	0·19 (0·145–0·242)	9·75 (7·51–12·2)	0·157 (0·125–0·195)	9·28 (7·27–11·6)	0·0925 (0·0753–0·114)	5·48 (4·36–6·85)
Lithuania	17·8 (17–18·6)	804 (767–845)	13·8 (13·1–14·5)	594 (563–625)	2·57 (2·23–2·93)	135 (117–155)	0·938 (0·771–1·13)	54·2 (43·7–66·4)	0·194 (0·152–0·242)	10·2 (7·71–13·1)	0·169 (0·133–0·21)	6·63 (5·23–8·32)	0·0999 (0·0808–0·124)	4·51 (3·6–5·56)
Luxembourg	10·9 (10·3–11·5)	97 (91·7–102)	8·64 (8·21–9·1)	74·6 (70·8–78·7)	1·78 (1·53–2·06)	17·7 (15·1–20·5)	0·308 (0·221–0·423)	3·22 (2·3–4·4)	0·0128 (0·00733–0·0254)	0·132 (0·0722–0·269)	0·0711 (0·0559–0·0905)	0·576 (0·454–0·732)	0·0764 (0·0607–0·0948)	0·667 (0·529–0·83)
Madagascar	20·5 (19·7–21·4)	3630 (3430–3850)	13·4 (12·7–14)	2350 (2230–2470)	4·46 (3·88–5·06)	813 (687–963)	1·78 (1·51–2·11)	281 (227–346)	0·488 (0·397–0·596)	86·7 (66·9–111)	0·204 (0·161–0·256)	48·3 (35·1–63·8)	0·236 (0·19–0·291)	51·5 (38·3–66·6)
Malawi	19 (18·3–19·8)	2160 (2050–2270)	13·7 (13·1–14·3)	1560 (1480–1640)	3·21 (2·78–3·61)	367 (309–433)	1·42 (1·2–1·69)	141 (114–174)	0·376 (0·308–0·453)	41·7 (32·5–53·1)	0·148 (0·116–0·186)	22·8 (16·6–30·3)	0·18 (0·146–0·22)	25·2 (18·9–32·7)
Malaysia	21·5 (20·8–22·3)	6420 (6200–6660)	16·6 (15·9–17·2)	5080 (4860–5290)	3·21 (2·8–3·6)	879 (762–998)	1·04 (0·868–1·25)	266 (221–322)	0·223 (0·173–0·277)	60·1 (46·1–75·6)	0·282 (0·221–0·356)	84·7 (65·7–108)	0·173 (0·139–0·215)	50·8 (40·1–63·5)
Maldives	21·9 (21·2–22·7)	97·2 (92·9–101)	16·5 (15·8–17·2)	77·4 (73·6–81·2)	3·49 (3·04–3·93)	12·5 (10·8–14·1)	1·15 (0·96–1·38)	3·91 (3·29–4·68)	0·263 (0·208–0·326)	1 (0·773–1·29)	0·314 (0·249–0·394)	1·42 (1·09–1·81)	0·216 (0·175–0·268)	0·957 (0·74–1·2)
Mali	19·2 (18·5–20)	2530 (2390–2670)	13·7 (13·1–14·3)	1790 (1700–1870)	3·17 (2·74–3·6)	421 (352–499)	1·38 (1·16–1·63)	164 (132–198)	0·507 (0·412–0·617)	73 (56·7–93·4)	0·254 (0·199–0·322)	49 (35·5–64·9)	0·228 (0·181–0·282)	38·8 (28·5–50·7)
Malta	11·1 (10·5–11·6)	84 (78·9–89·2)	8·56 (8·13–9·02)	61·4 (57·9–65)	1·85 (1·59–2·14)	16·8 (14·3–19·7)	0·462 (0·347–0·586)	4·29 (3·14–5·6)	0·0406 (0·0207–0·0627)	0·356 (0·161–0·575)	0·0751 (0·0586–0·095)	0·503 (0·387–0·65)	0·0856 (0·0681–0·106)	0·624 (0·491–0·776)
Marshall Islands	21·5 (20·8–22·3)	9·74 (9·35–10·1)	16·5 (15·9–17·2)	7·85 (7·49–8·19)	3·15 (2·77–3·56)	1·18 (1·02–1·36)	1·07 (0·901–1·28)	0·359 (0·296–0·437)	0·277 (0·219–0·341)	0·11 (0·0839–0·141)	0·279 (0·22–0·35)	0·142 (0·108–0·182)	0·2 (0·161–0·245)	0·0946 (0·0731–0·119)
Mauritania	18·6 (18–19·4)	521 (497–544)	13·8 (13·2–14·4)	388 (369–406)	2·8 (2·43–3·16)	76·8 (65·5–88·9)	1·22 (1·03–1·44)	30·7 (25·2–37·1)	0·43 (0·348–0·521)	12·2 (9·69–15·2)	0·219 (0·172–0·277)	7·92 (5·88–10·3)	0·167 (0·135–0·206)	5·36 (4·09–6·9)
Mauritius	21·5 (20·8–22·3)	346 (335–358)	16·5 (15·8–17·1)	267 (255–276)	3·27 (2·86–3·68)	53·2 (46·1–60·6)	1·06 (0·886–1·28)	16·4 (13·5–20·1)	0·232 (0·18–0·29)	3·39 (2·62–4·3)	0·291 (0·229–0·367)	3·9 (3·09–4·87)	0·183 (0·146–0·227)	2·64 (2·11–3·24)
Mexico	22·1 (21–23·3)	27200 (25900–28700)	17·3 (16·3–18·3)	21600 (20400–22900)	3·23 (2·81–3·69)	3800 (3290–4340)	1·08 (0·887–1·31)	1220 (990–1470)	0·206 (0·163–0·257)	236 (187–295)	0·216 (0·17–0·268)	261 (204–327)	0·0905 (0·0738–0·111)	107 (86·4–131)
Moldova	18 (17·2–18·8)	927 (887–969)	13·7 (13–14·4)	699 (663–735)	2·76 (2·39–3·15)	148 (127–170)	1·02 (0·844–1·23)	55·3 (45–67·8)	0·221 (0·175–0·274)	11·2 (8·75–14·2)	0·184 (0·144–0·229)	8·02 (6·26–10)	0·12 (0·0973–0·149)	5·71 (4·63–6·98)
Monaco	10·9 (10·4–11·5)	8·11 (7·62–8·63)	8·59 (8·17–9·04)	5·87 (5·54–6·23)	1·76 (1·51–2·02)	1·67 (1·41–1·95)	0·413 (0·31–0·526)	0·424 (0·313–0·553)	0·0306 (0·0142–0·0516)	0·0301 (0·013–0·0534)	0·0704 (0·0553–0·0894)	0·0469 (0·0362–0·0605)	0·075 (0·0595–0·0929)	0·0562 (0·044–0·0709)
Mongolia	18 (17·3–18·8)	512 (489–536)	13·7 (13–14·3)	409 (388–430)	2·85 (2·46–3·27)	67·7 (57·9–77·8)	1 (0·831–1·21)	21 (17·2–25·5)	0·204 (0·157–0·256)	4·79 (3·64–6·07)	0·19 (0·15–0·238)	5·45 (4·12–6·96)	0·129 (0·104–0·158)	3·49 (2·75–4·42)
Montenegro	17·8 (17–18·6)	153 (146–160)	13·8 (13·2–14·5)	118 (112–124)	2·67 (2·33–3·02)	24·1 (20·8–27·7)	0·884 (0·729–1·07)	7·88 (6·31–9·76)	0·157 (0·117–0·201)	1·32 (0·957–1·76)	0·174 (0·138–0·216)	1·27 (0·998–1·58)	0·106 (0·0859–0·13)	0·837 (0·677–1·03)
Morocco	12·8 (12·2–13·4)	4200 (3990–4410)	8·16 (7·73–8·61)	2820 (2670–2980)	2·99 (2·59–3·4)	902 (778–1040)	1·11 (0·923–1·35)	308 (252–373)	0·23 (0·18–0·286)	66·6 (51·7–82·6)	0·176 (0·14–0·219)	57·4 (45·1–72·1)	0·12 (0·0981–0·148)	39 (31·4–48·8)
Mozambique	19·2 (18·4–19·9)	3340 (3160–3510)	13·7 (13–14·3)	2380 (2260–2500)	3·3 (2·88–3·72)	585 (493–690)	1·46 (1·23–1·72)	224 (182–277)	0·389 (0·32–0·472)	68 (52·4–86·1)	0·153 (0·121–0·193)	37·3 (27·3–49·7)	0·193 (0·157–0·239)	42·9 (31·8–56·3)
Myanmar	22·5 (21·7–23·3)	11600 (11200–12000)	16·1 (15·5–16·8)	8520 (8160–8870)	4·04 (3·57–4·52)	1950 (1700–2190)	1·32 (1·12–1·58)	605 (503–724)	0·32 (0·252–0·396)	155 (121–192)	0·396 (0·311–0·499)	209 (163–265)	0·279 (0·222–0·347)	145 (113–182)
Namibia	19·1 (18·3–19·9)	346 (330–361)	14·4 (13·8–15·1)	267 (254–279)	2·48 (2·16–2·82)	42·2 (36·2–48·6)	1·35 (1·13–1·59)	21·7 (18–25·9)	0·418 (0·338–0·512)	7·12 (5·65–8·79)	0·22 (0·174–0·279)	4·62 (3·56–5·97)	0·184 (0·151–0·226)	3·53 (2·76–4·47)
Nauru	21·3 (20·6–22·1)	1·49 (1·42–1·55)	16·6 (15·9–17·3)	1·23 (1·17–1·28)	3·01 (2·63–3·4)	0·156 (0·13–0·183)	1·02 (0·85–1·23)	0·0475 (0·0382–0·0591)	0·254 (0·198–0·317)	0·0159 (0·0118–0·0212)	0·264 (0·206–0·329)	0·0237 (0·0174–0·0309)	0·177 (0·142–0·218)	0·0139 (0·0104–0·0181)
Nepal	19·8 (19–20·6)	5020 (4800–5230)	15·2 (14·5–16)	3950 (3760–4140)	2·8 (2·44–3·2)	662 (568–761)	1·28 (1·05–1·53)	279 (230–335)	0·0927 (0·0725–0·115)	21·3 (16·6–26·6)	0·245 (0·194–0·306)	67·2 (51·8–85·3)	0·144 (0·117–0·178)	37·1 (29·5–46·5)
Netherlands	10·9 (10·4–11·5)	3090 (2920–3280)	8·61 (8·17–9·06)	2320 (2190–2450)	1·79 (1·54–2·07)	601 (510–697)	0·366 (0·263–0·482)	125 (88·3–169)	0·02 (0·00918–0·0375)	6·55 (2·85–12·9)	0·0716 (0·056–0·0901)	18·1 (14–23·1)	0·0775 (0·0615–0·0967)	21·3 (16·5–26·6)
New Zealand	13·6 (12·9–14·4)	925 (870–984)	9·73 (9·21–10·3)	630 (594–671)	2·78 (2·44–3·16)	212 (184–243)	0·73 (0·601–0·876)	56·6 (46·3–68·1)	0·112 (0·0865–0·145)	8·32 (6·31–10·8)	0·151 (0·12–0·19)	9·4 (7·41–12)	0·143 (0·117–0·175)	9·49 (7·63–11·6)

Nicaragua	22 (20·9–23·3)	1160 (1100–1220)	17·2 (16·2–18·3)	934 (880–988)	3·21 (2·78–3·66)	151 (131–173)	1·06 (0·862–1·31)	45·3 (36·9–55·2)	0·199 (0·156–0·248)	9·03 (7·03–11·4)	0·208 (0·163–0·26)	11·7 (9·11–14·9)	0·0944 (0·0772–0·116)	4·75 (3·81–5·91)
Niger	19·4 (18·6–20·2)	2530 (2380–2670)	13·6 (12·9–14·1)	1730 (1640–1810)	3·33 (2·88–3·77)	446 (368–530)	1·45 (1·23–1·72)	171 (138–211)	0·542 (0·443–0·659)	80·5 (61·3–104)	0·271 (0·213–0·342)	55·5 (39·9–73·6)	0·261 (0·21–0·326)	46·9 (34·4–62)
Nigeria	18·8 (18·1–19·6)	26500 (25000–28000)	12·8 (12·2–13·3)	17700 (16800–18600)	3·31 (2·87–3·74)	4740 (3950–5640)	1·44 (1·22–1·7)	1880 (1530–2310)	0·781 (0·643–0·953)	1250 (963–1590)	0·285 (0·224–0·363)	571 (418–759)	0·217 (0·173–0·269)	383 (286–491)
Niue	21·2 (20·5–21·9)	0·416 (0·402–0·43)	16·7 (16–17·3)	0·326 (0·313–0·338)	2·88 (2·51–3·24)	0·0589 (0·0512–0·0668)	0·959 (0·802–1·15)	0·0194 (0·016–0·0234)	0·228 (0·177–0·286)	0·00439 (0·0034–0·00557)	0·247 (0·194–0·312)	0·00437 (0·00343–0·00547)	0·155 (0·125–0·193)	0·00288 (0·00236–0·00353)
North Korea	21·6 (20·9–22·3)	6680 (6460–6920)	16·7 (16–17·4)	5210 (5000–5430)	3·09 (2·7–3·5)	948 (825–1070)	1·07 (0·893–1·28)	313 (256–382)	0·298 (0·236–0·368)	84·3 (66·1–105)	0·259 (0·206–0·321)	69·6 (55·4–86·4)	0·194 (0·156–0·238)	54·7 (44–67·3)
North Macedonia	17·9 (17·1–18·7)	521 (498–545)	13·8 (13·1–14·5)	405 (385–426)	2·73 (2·37–3·11)	79·6 (67·9–91·6)	0·913 (0·749–1·1)	25·1 (20–31)	0·166 (0·125–0·211)	4·37 (3·16–5·76)	0·179 (0·14–0·225)	4·31 (3·34–5·41)	0·113 (0·0914–0·139)	2·89 (2·32–3·58)
Northern Mariana Islands	21·1 (20·4–21·9)	10·9 (10·5–11·3)	16·7 (16·1–17·4)	8·89 (8·54–9·24)	2·85 (2·5–3·21)	1·34 (1·15–1·57)	0·948 (0·79–1·14)	0·387 (0·313–0·482)	0·223 (0·173–0·28)	0·0908 (0·069–0·118)	0·243 (0·192–0·305)	0·101 (0·0794–0·128)	0·15 (0·121–0·184)	0·0658 (0·0522–0·0821)
Norway	12·5 (11·8–13·3)	1040 (970–1110)	9·12 (8·61–9·66)	717 (671–770)	2·58 (2·23–2·95)	243 (209–278)	0·573 (0·468–0·692)	56·2 (45·6–68)	0·0501 (0·0382–0·0655)	4·85 (3·63–6·35)	0·0812 (0·0636–0·102)	6·11 (4·77–7·81)	0·107 (0·0864–0·133)	8·68 (6·92–10·8)
Oman	12·5 (11·9–13·2)	376 (356–398)	8·43 (7·99–8·89)	290 (272–310)	2·7 (2·36–3·06)	57·7 (49·7–66·4)	0·975 (0·806–1·17)	17 (13·8–20·7)	0·184 (0·14–0·233)	3·94 (2·87–5·19)	0·155 (0·123–0·192)	5·09 (3·84–6·6)	0·089 (0·0724–0·11)	2·83 (2·15–3·62)
Pakistan	20·2 (19·4–21)	30200 (28900–31500)	15·3 (14·7–16)	23700 (22600–24800)	2·94 (2·56–3·32)	3960 (3390–4540)	1·2 (1·01–1·42)	1450 (1200–1730)	0·378 (0·306–0·464)	519 (413–648)	0·212 (0·17–0·265)	398 (298–516)	0·123 (0·101–0·151)	201 (155–255)
Palau	21·2 (20·5–22)	4·56 (4·41–4·72)	16·7 (16·1–17·4)	3·72 (3·57–3·87)	2·89 (2·53–3·26)	0·561 (0·478–0·647)	0·962 (0·8–1·16)	0·164 (0·133–0·201)	0·229 (0·175–0·283)	0·0392 (0·0299–0·0503)	0·247 (0·195–0·31)	0·0427 (0·0333–0·0536)	0·155 (0·126–0·191)	0·0284 (0·0226–0·0353)
Palestine	12·7 (12·1–13·3)	387 (368–407)	8·2 (7·75–8·65)	272 (258–287)	2·89 (2·53–3·26)	74·4 (64·6–84·4)	1·07 (0·89–1·29)	24·3 (20·2–28·9)	0·215 (0·167–0·268)	5·7 (4·39–7·18)	0·169 (0·135–0·211)	6·17 (4·71–7·91)	0·109 (0·0888–0·135)	3·76 (2·91–4·81)
Panama	21·8 (20·7–23)	911 (865–958)	17·5 (16·4–18·5)	730 (687–773)	2·96 (2·57–3·39)	123 (107–140)	0·968 (0·789–1·19)	40·3 (33–49·4)	0·172 (0·132–0·218)	7·18 (5·5–9·05)	0·188 (0·149–0·236)	7·86 (6·22–9·83)	0·0772 (0·0625–0·0945)	3·24 (2·61–3·96)
Papua New Guinea	21·8 (21·1–22·6)	1520 (1450–1580)	16·4 (15·7–17)	1190 (1140–1240)	3·4 (2·97–3·83)	195 (167–223)	1·17 (0·983–1·4)	62·1 (51·1–75·3)	0·318 (0·254–0·387)	21·3 (16·4–27·2)	0·309 (0·244–0·389)	27·1 (20·2–35·3)	0·247 (0·196–0·306)	20 (15–25·6)
Paraguay	22·2 (21·2–23·4)	1360 (1300–1430)	16·7 (15·7–17·7)	1040 (984–1110)	4·15 (3·59–4·71)	240 (208–273)	0·955 (0·768–1·18)	51·8 (41·7–64·1)	0·195 (0·156–0·242)	11·1 (8·92–13·8)	0·191 (0·151–0·236)	12·1 (9·46–15)	0·0596 (0·048–0·0739)	3·46 (2·82–4·31)
Peru	21·8 (20·6–23·1)	7210 (6820–7630)	18·3 (17·2–19·5)	6090 (5720–6460)	1·82 (1·53–2·12)	588 (499–686)	1·24 (1·01–1·5)	401 (329–484)	0·2 (0·158–0·253)	65·4 (52·1–81·8)	0·151 (0·119–0·188)	50·3 (39·7–62·2)	0·066 (0·0529–0·0813)	21·8 (17·6–26·8)
Philippines	22·2 (21·4–22·9)	20900 (20100–21700)	16·4 (15·8–17·1)	15900 (15300–16600)	3·66 (3·22–4·09)	3120 (2720–3520)	1·2 (1·02–1·44)	963 (801–1160)	0·277 (0·224–0·341)	247 (197–310)	0·346 (0·275–0·432)	365 (282–464)	0·239 (0·192–0·293)	238 (186–296)
Poland	18·2 (17·4–19)	10600 (10100–11000)	13·8 (13·1–14·5)	7780 (7390–8200)	2·82 (2·47–3·2)	1780 (1540–2050)	1·04 (0·867–1·25)	692 (566–840)	0·227 (0·183–0·281)	138 (110–174)	0·192 (0·152–0·24)	93·5 (74·4–117)	0·117 (0·0947–0·144)	64·3 (51·8–78·4)
Portugal	11·1 (10·5–11·7)	2130 (2010–2260)	8·49 (8·06–8·92)	1510 (1430–1600)	1·89 (1·61–2·18)	451 (383–524)	0·488 (0·38–0·614)	125 (94·7–161)	0·0468 (0·0263–0·0699)	11·1 (5·43–17·2)	0·0773 (0·0608–0·097)	13·3 (10·2–17·1)	0·0924 (0·0743–0·115)	17·4 (13·6–21·9)
Puerto Rico	21·7 (20·6–22·9)	1210 (1130–1280)	17·6 (16·5–18·6)	930 (868–995)	2·77 (2·39–3·19)	181 (155–208)	0·959 (0·775–1·17)	69·5 (55·8–86·1)	0·188 (0·149–0·235)	12·6 (9·75–16·1)	0·176 (0·139–0·218)	9·11 (7·12–11·4)	0·0663 (0·0535–0·082)	4·14 (3·27–5·14)
Qatar	12·7 (12·1–13·3)	252 (237–268)	8·62 (8·13–9·08)	201 (188–215)	2·69 (2·34–3·09)	35 (29·3–40·9)	0·959 (0·787–1·17)	9·56 (7·53–12·1)	0·178 (0·133–0·228)	2·19 (1·47–2·99)	0·151 (0·12–0·188)	3·1 (2·3–4·03)	0·0845 (0·0685–0·104)	1·7 (1·27–2·24)
Romania	17·8 (17·1–18·6)	5380 (5140–5640)	13·8 (13·2–14·4)	4020 (3810–4220)	2·7 (2·37–3·06)	915 (796–1040)	0·899 (0·745–1·09)	321 (256–394)	0·162 (0·123–0·206)	53·3 (38·7–70·2)	0·176 (0·139–0·221)	45 (35·5–56·3)	0·11 (0·0887–0·136)	31·7 (25·3–38·9)
Russia	18·1 (17·4–18·9)	37300 (35700–39000)	13·8 (13·1–14·4)	28000 (26600–29400)	2·81 (2·45–3·19)	6070 (5260–6950)	1·02 (0·846–1·22)	2240 (1830–2730)	0·214 (0·172–0·265)	441 (348–557)	0·192 (0·152–0·24)	332 (264–415)	0·117 (0·0948–0·144)	224 (181–273)
Rwanda	18·9 (18·2–19·7)	1640 (1560–1720)	13·7 (13·1–14·4)	1210 (1150–1270)	3·1 (2·69–3·51)	262 (222–307)	1·36 (1·15–1·62)	101 (82·1–122)	0·359 (0·292–0·437)	28·8 (22·7–36·5)	0·142 (0·111–0·179)	15·2 (11·4–20)	0·165 (0·134–0·202)	16·2 (12·3–20·8)
Saint Kitts and Nevis	21·9 (20·8–23·1)	15 (14·2–16)	17·5 (16·5–18·6)	12·4 (11·6–13·2)	2·89 (2·48–3·33)	1·83 (1·54–2·14)	0·999 (0·807–1·22)	0·566 (0·448–0·71)	0·198 (0·157–0·247)	0·112 (0·0871–0·142)	0·183 (0·146–0·23)	0·109 (0·0848–0·138)	0·0719 (0·0583–0·0883)	0·0427 (0·034–0·0528)
Saint Lucia	22 (20·8–23·1)	46·1 (43·7–48·7)	17·4 (16·4–18·5)	36·7 (34·5–39)	3 (2·58–3·46)	6·25 (5·32–7·22)	1·04 (0·838–1·27)	2·14 (1·71–2·63)	0·209 (0·166–0·262)	0·418 (0·327–0·528)	0·192 (0·151–0·238)	0·363 (0·282–0·453)	0·0791 (0·0638–0·0978)	0·158 (0·127–0·195)
Saint Vincent and the Grenadines	22 (20·9–23·2)	28·9 (27·3–30·5)	17·4 (16·4–18·5)	22·9 (21·5–24·4)	3·07 (2·63–3·54)	4·02 (3·43–4·66)	1·06 (0·863–1·3)	1·36 (1·08–1·67)	0·216 (0·171–0·268)	0·267 (0·211–0·337)	0·195 (0·153–0·243)	0·235 (0·182–0·295)	0·0828 (0·0667–0·102)	0·103 (0·0826–0·127)
Samoa	21·5 (20·8–22·2)	36·8 (35·4–38·1)	16·6 (15·9–17·2)	28·9 (27·7–30·1)	3·12 (2·73–3·51)	4·88 (4·25–5·51)	1·06 (0·885–1·27)	1·6 (1·33–1·91)	0·27 (0·212–0·336)	0·455 (0·353–0·57)	0·275 (0·215–0·345)	0·548 (0·419–0·703)	0·194 (0·155–0·238)	0·359 (0·278–0·449)
San Marino	11 (10·4–11·5)	5·83 (5·51–6·18)	8·57 (8·14–9·03)	4·28 (4·05–4·52)	1·78 (1·53–2·06)	1·16 (0·98–1·34)	0·425 (0·317–0·543)	0·298 (0·221–0·382)	0·0328 (0·0156–0·0535)	0·0222 (0·01–0·037)	0·0712 (0·0557–0·0898)	0·0352 (0·0277–0·0446)	0·0773 (0·0614–0·0954)	0·0418 (0·0331–0·052)
Saudi Arabia	12·5 (11·9–13·1)	3270 (3090–3450)	8·42 (7·96–8·88)	2500 (2350–2650)	2·7 (2·35–3·06)	521 (444–598)	0·971 (0·795–1·18)	155 (126–187)	0·182 (0·138–0·229)	34·5 (25·6–44·6)	0·154 (0·123–0·191)	41·5 (31·5–53·2)	0·0878 (0·071–0·109)	23·3 (17·7–30·1)
Senegal	18·9 (18·2–19·6)	1960 (1870–2050)	13·7 (13·1–14·3)	1430 (1360–1500)	2·97 (2·58–3·36)	303 (259–352)	1·3 (1·1–1·54)	120 (98·8–145)	0·466 (0·379–0·569)	49·4 (39–62·2)	0·235 (0·185–0·297)	32·1 (23·6–41·6)	0·195 (0·155–0·239)	23·7 (18–30·4)
Serbia	17·8 (17·1–18·7)	2340 (2230–2460)	13·8 (13·2–14·5)	1770 (1670–1860)	2·7 (2·35–3·07)	390 (335–451)	0·898 (0·74–1·08)	130 (103–161)	0·162 (0·123–0·207)	21·7 (15·8–29·2)	0·176 (0·138–0·219)	19·5 (15·2–24·6)	0·11 (0·0889–0·135)	13·2 (10·6–16·5)
Seychelles	21·6 (20·9–22·3)	24·4 (23·6–25·3)	16·5 (15·9–17·2)	19·2 (18·3–19·9)	3·25 (2·85–3·64)	3·46 (3·01–3·92)	1·05 (0·882–1·27)	1·06 (0·881–1·29)	0·228 (0·176–0·285)	0·23 (0·176–0·289)	0·287 (0·226–0·364)	0·287 (0·225–0·362)	0·18 (0·145–0·222)	0·187 (0·15–0·232)
Sierra Leone	20·3 (19·4–21·3)	1060 (1000–1110)	15·4 (14·6–16·3)	818 (774–862)	2·88 (2·5–3·27)	143 (121–166)	1·27 (1·06–1·51)	55·8 (45·8–66·8)	0·296 (0·239–0·361)	15·1 (11·7–19)	0·214 (0·167–0·27)	14·8 (11–19·3)	0·187 (0·152–0·23)	11·5 (8·81–14·8)
Singapore	13·5 (12·8–14·1)	1030 (975–1080)	9·68 (9·2–10·2)	747 (707–788)	2·76 (2·4–3·13)	206 (177–235)	0·659 (0·537–0·814)	47·2 (38·1–58·8)	0·104 (0·0629–0·147)	7·38 (4·42–10·6)	0·133 (0·105–0·166)	8·97 (6·95–11·5)	0·135 (0·109–0·167)	9·63 (7·7–11·9)
Slovakia	17·7 (17–18·5)	1410 (1350–1480)	13·8 (13·2–14·5)	1080 (1030–1140)	2·62 (2·29–2·97)	221 (191–254)	0·866 (0·713–1·05)	73·3 (58·7–90·7)	0·151 (0·112–0·194)	12 (8·75–16·1)	0·171 (0·134–0·214)	11·3 (8·9–14·2)	0·103 (0·0829–0·127)	7·53 (6·08–9·28)
Slovenia	17·6 (16·8–18·4)	597 (569–627)	13·9 (13·2–14·6)	453 (430–475)	2·63 (2·3–2·98)	105 (91·3–119)	0·683 (0·543–0·846)	29·1 (22·6–36·5)	0·0647 (0·0323–0·102)	2·66 (1·16–4·41)	0·168 (0·132–0·21)	4·9 (3·87–6·16)	0·0996 (0·0804–0·123)	3·36 (2·68–4·13)
Solomon Islands	21·8 (21·1–22·6)	97·8 (93·7–102)	16·4 (15·7–17·1)	76·8 (73·3–80·2)	3·37 (2·95–3·79)	12·5 (10·7–14·3)	1·16 (0·975–1·38)	3·96 (3·27–4·79)	0·314 (0·251–0·382)	1·38 (1·05–1·76)	0·306 (0·242–0·383)	1·78 (1·32–2·3)	0·243 (0·195–0·299)	1·29 (0·966–1·63)
Somalia	19·8 (19–20·7)	2330 (2190–2460)	13·5 (12·9–14·1)	1570 (1490–1660)	3·76 (3·27–4·24)	453 (374–535)	1·66 (1·39–1·98)	172 (136–210)	0·455 (0·373–0·553)	54·8 (41·9–70·9)	0·179 (0·14–0·225)	30·4 (22–40·8)	0·27 (0·217–0·333)	43·7 (31·9–57·3)
South Africa	20·3 (19·5–21·1)	10400 (9930–10800)	14·1 (13·5–14·8)	7410 (7060–7770)	3·58 (3·13–4·01)	1740 (1510–1970)	1·53 (1·3–1·79)	710 (597–839)	0·499 (0·409–0·609)	239 (194–293)	0·301 (0·241–0·379)	158 (124–201)	0·237 (0·192–0·291)	120 (95·5–149)
South Korea	13·4 (12·7–14)	10900 (10300–11500)	9·65 (9·16–10·1)	7780 (7350–8210)	2·72 (2·36–3·11)	2330 (2000–2680)	0·644 (0·52–0·793)	532 (419–669)	0·1 (0·0611–0·143)	80 (45·3–117)	0·132 (0·105–0·164)	95·4 (73·6–123)	0·133 (0·108–0·164)	102 (80·9–127)

South Sudan	19 (18·3–19·8)	1110 (1050–1160)	13·7 (13·1–14·4)	805 (767–845)	3·17 (2·76–3·57)	185 (156–217)	1·42 (1·2–1·68)	71·9 (58·5–87·9)	0·381 (0·313–0·464)	21·3 (16·6–27)	0·147 (0·116–0·184)	11·3 (8·33–15·1)	0·176 (0·143–0·216)	12·4 (9·33–16·1)
Spain	11·2 (10·6–11·8)	8760 (8280–9280)	8·48 (8·06–8·92)	6170 (5830–6530)	1·84 (1·57–2·13)	1740 (1480–2020)	0·616 (0·49–0·763)	643 (505–801)	0·0933 (0·0699–0·119)	88·6 (64·2–117)	0·0764 (0·0598–0·0967)	54·1 (42·3–68·6)	0·0895 (0·0719–0·112)	69·8 (55·2–87·7)
Sri Lanka	21·8 (21·1–22·6)	5350 (5180–5540)	16·4 (15·7–17)	4050 (3880–4200)	3·53 (3·09–3·96)	866 (753–980)	1·14 (0·955–1·37)	265 (218–323)	0·255 (0·199–0·317)	57·8 (44·5–73·3)	0·323 (0·251–0·406)	71·4 (56·1–89·3)	0·205 (0·165–0·256)	46·7 (37·5–57·7)
Sudan	12·9 (12·3–13·5)	3140 (2980–3300)	8·17 (7·73–8·61)	2150 (2030–2260)	3·03 (2·65–3·43)	636 (552–718)	1·14 (0·936–1·38)	215 (178–257)	0·236 (0·186–0·296)	52·2 (40·7–65·4)	0·179 (0·143–0·222)	54 (41·5–69·3)	0·124 (0·101–0·152)	35·4 (27·4–44·7)
Suriname	22 (20·9–23·2)	134 (127–142)	17·4 (16·3–18·4)	107 (101–114)	3·04 (2·63–3·47)	18 (15·4–20·6)	1·06 (0·855–1·29)	5·99 (4·83–7·33)	0·214 (0·169–0·265)	1·21 (0·959–1·52)	0·195 (0·155–0·243)	1·12 (0·89–1·41)	0·0822 (0·0667–0·101)	0·474 (0·385–0·583)
Sweden	9·52 (9·09–9·95)	1540 (1470–1610)	7·58 (7·21–7·95)	1140 (1090–1200)	1·57 (1·36–1·8)	321 (277–370)	0·22 (0·177–0·274)	46·8 (36·3–60)	0·00776 (0·00584–0·0111)	1·55 (1·1–2·4)	0·0744 (0·0587–0·094)	10·5 (8·25–13·4)	0·0681 (0·0523–0·0867)	11·1 (8·54–13·9)
Switzerland	10·8 (10·2–11·4)	1560 (1470–1660)	8·64 (8·2–9·09)	1180 (1120–1250)	1·73 (1·47–2)	306 (259–356)	0·283 (0·198–0·387)	53·9 (37·3–74·3)	0·0112 (0·00687–0·0203)	2·13 (1·2–4)	0·0694 (0·0544–0·0872)	9·19 (7·09–11·6)	0·0727 (0·0573–0·0897)	10·6 (8·36–13·2)
Syria	12·6 (12–13·3)	1630 (1550–1720)	8·2 (7·74–8·64)	1120 (1060–1180)	2·87 (2·49–3·26)	339 (291–389)	1·06 (0·874–1·28)	112 (91·2–136)	0·214 (0·165–0·268)	23·7 (18·2–30·2)	0·167 (0·134–0·208)	21·5 (16·7–27)	0·107 (0·0868–0·131)	13·5 (10·7–17)
São Tomé and PrÍncipe	18·6 (18–19·4)	27·6 (26·3–28·9)	13·8 (13·2–14·4)	20·9 (19·9–21·9)	2·78 (2·41–3·14)	3·92 (3·33–4·5)	1·21 (1·02–1·44)	1·55 (1·28–1·87)	0·426 (0·346–0·516)	0·614 (0·487–0·764)	0·217 (0·17–0·272)	0·399 (0·299–0·517)	0·165 (0·132–0·204)	0·272 (0·207–0·35)
Taiwan (province of China)	20·5 (19·8–21·2)	6880 (6670–7100)	16·7 (16–17·3)	5530 (5320–5730)	2·45 (2·13–2·76)	898 (775–1020)	0·834 (0·701–1)	300 (248–362)	0·216 (0·165–0·269)	70·9 (54–89·6)	0·201 (0·159–0·252)	54·7 (43·1–68·4)	0·108 (0·0875–0·133)	33·5 (27·1–41)
Tajikistan	18·2 (17·5–19)	1230 (1170–1290)	13·6 (13–14·2)	970 (921–1020)	2·99 (2·62–3·4)	168 (143–193)	1·06 (0·882–1·28)	51·2 (41·9–62·3)	0·223 (0·175–0·279)	12·9 (9·84–16·5)	0·202 (0·158–0·254)	15·8 (12–20·3)	0·145 (0·117–0·178)	10·2 (7·9–13)
Tanzania	18·3 (17·6–19)	6520 (6220–6830)	14 (13·3–14·6)	5070 (4820–5310)	2·5 (2·19–2·85)	869 (738–1020)	1·2 (1·01–1·42)	367 (304–448)	0·316 (0·258–0·385)	106 (83·9–134)	0·119 (0·0943–0·15)	53·9 (40·5–71)	0·141 (0·115–0·173)	58 (44·4–74·8)
Thailand	21·3 (20·6–22·1)	19800 (19100–20500)	16·6 (15·9–17·2)	15400 (14800–16000)	3·07 (2·67–3·48)	2910 (2510–3320)	1·01 (0·837–1·22)	934 (762–1140)	0·22 (0·171–0·273)	190 (147–239)	0·267 (0·21–0·335)	201 (158–251)	0·172 (0·138–0·213)	143 (116–175)
The Bahamas	21·8 (20·6–23)	88·9 (84·1–94)	17·6 (16·5–18·6)	73·2 (68·7–77·7)	2·8 (2·43–3·23)	10·7 (9·15–12·4)	0·969 (0·784–1·19)	3·45 (2·76–4·26)	0·191 (0·151–0·237)	0·682 (0·537–0·858)	0·178 (0·141–0·223)	0·665 (0·524–0·832)	0·0678 (0·0551–0·0834)	0·251 (0·203–0·312)
The Gambia	18·9 (18·2–19·7)	272 (259–286)	13·7 (13·2–14·4)	199 (189–208)	2·95 (2·55–3·33)	42 (35·4–49·1)	1·29 (1·09–1·53)	16·6 (13·6–20·3)	0·46 (0·373–0·559)	6·97 (5·42–8·86)	0·233 (0·182–0·297)	4·67 (3·4–6·26)	0·191 (0·153–0·237)	3·36 (2·53–4·4)
Timor-Leste	22 (21·3–22·8)	218 (210–227)	16·3 (15·7–17)	164 (157–170)	3·61 (3·16–4·03)	33·7 (29·1–38)	1·2 (1·01–1·44)	10·6 (8·75–12·8)	0·286 (0·226–0·353)	2·89 (2·2–3·68)	0·334 (0·262–0·418)	4·22 (3·18–5·48)	0·244 (0·196–0·303)	2·81 (2·14–3·61)
Togo	18·7 (18–19·5)	1010 (965–1070)	13·7 (13·1–14·3)	757 (720–795)	2·88 (2·5–3·25)	148 (126–173)	1·27 (1·07–1·51)	57·5 (46·9–70)	0·449 (0·368–0·547)	23·9 (18·7–30·4)	0·229 (0·18–0·29)	15·9 (11·7–20·8)	0·184 (0·148–0·227)	11·3 (8·54–14·6)
Tokelau	21·3 (20·6–22·1)	0·285 (0·276–0·296)	16·6 (16–17·3)	0·224 (0·215–0·233)	2·99 (2·62–3·37)	0·0394 (0·0343–0·0448)	1·01 (0·844–1·21)	0·0127 (0·0106–0·0154)	0·249 (0·193–0·309)	0·00325 (0·00252–0·00406)	0·259 (0·203–0·323)	0·00353 (0·00276–0·00441)	0·171 (0·138–0·212)	0·00229 (0·00183–0·00284)
Tonga	21·3 (20·6–22·1)	18·5 (17·9–19·2)	16·6 (15·9–17·2)	14·5 (13·9–15·1)	3·04 (2·67–3·42)	2·53 (2·21–2·84)	1·03 (0·86–1·23)	0·851 (0·714–1·01)	0·256 (0·201–0·318)	0·227 (0·177–0·283)	0·266 (0·211–0·331)	0·26 (0·202–0·327)	0·18 (0·145–0·221)	0·167 (0·132–0·209)
Trinidad and Tobago	21·9 (20·7–23·1)	383 (362–406)	17·5 (16·5–18·6)	307 (287–328)	2·87 (2·47–3·3)	50·9 (43·4–59·1)	0·99 (0·801–1·21)	17·2 (13·7–21·3)	0·196 (0·154–0·243)	3·27 (2·55–4·14)	0·182 (0·144–0·228)	2·82 (2·21–3·54)	0·0709 (0·0573–0·0875)	1·18 (0·952–1·46)
Tunisia	12·5 (11·9–13·2)	1570 (1490–1650)	8·23 (7·78–8·66)	1050 (998–1110)	2·8 (2·44–3·18)	338 (292–385)	1·03 (0·846–1·25)	118 (96·8–145)	0·202 (0·155–0·253)	23·4 (17·8–29·5)	0·163 (0·13–0·202)	19·1 (15–23·8)	0·1 (0·0817–0·124)	11·9 (9·63–14·8)
Turkey	12·4 (11·8–13·1)	11000 (10500–11600)	8·27 (7·82–8·71)	7460 (7050–7870)	2·72 (2·38–3·09)	2330 (2030–2660)	0·992 (0·817–1·2)	825 (677–1000)	0·191 (0·146–0·242)	159 (122–203)	0·158 (0·126–0·195)	133 (105–165)	0·0929 (0·0756–0·114)	79·4 (64·4–98·5)
Turkmenistan	17·9 (17·2–18·7)	793 (760–828)	13·7 (13·1–14·4)	631 (600–662)	2·75 (2·4–3·14)	107 (93–124)	0·956 (0·79–1·15)	34·3 (28·5–41·2)	0·189 (0·145–0·237)	7·32 (5·61–9·2)	0·182 (0·144–0·228)	8·22 (6·32–10·4)	0·117 (0·0947–0·144)	5·04 (3·98–6·27)
Tuvalu	21·4 (20·8–22·2)	2·34 (2·27–2·43)	16·6 (15·9–17·3)	1·84 (1·77–1·92)	3·09 (2·71–3·48)	0·32 (0·277–0·364)	1·05 (0·876–1·25)	0·101 (0·0839–0·122)	0·265 (0·209–0·325)	0·027 (0·0208–0·0334)	0·271 (0·213–0·34)	0·0309 (0·0239–0·0393)	0·188 (0·152–0·232)	0·0207 (0·0163–0·0258)
UK	13 (12·4–13·6)	13100 (12400–13800)	9·23 (8·8–9·68)	8690 (8250–9130)	2·92 (2·58–3·26)	3490 (3060–3910)	0·5 (0·417–0·601)	606 (497–740)	0·0246 (0·0181–0·0337)	28·2 (20·3–40·4)	0·15 (0·119–0·189)	134 (106–169)	0·176 (0·143–0·216)	169 (136–206)
USA	14·7 (13·9–15·5)	72900 (68500–77300)	9·9 (9·3–10·5)	46900 (43900–50100)	3·33 (2·91–3·76)	18200 (15900–20600)	0·908 (0·753–1·09)	5020 (4140–6060)	0·191 (0·149–0·243)	985 (765–1260)	0·182 (0·143–0·228)	838 (653–1070)	0·201 (0·164–0·247)	962 (776–1180)
Uganda	16·2 (15·7–16·9)	3830 (3640–4010)	12·9 (12·3–13·4)	3070 (2910–3220)	2·01 (1·73–2·26)	465 (385–549)	0·908 (0·756–1·08)	180 (146–222)	0·239 (0·195–0·289)	53·6 (41·6–68·8)	0·0914 (0·0717–0·116)	29·7 (21·4–39·7)	0·109 (0·0885–0·133)	31·5 (23·5–41·5)
Ukraine	18·2 (17·4–19)	11900 (11300–12400)	13·7 (13·1–14·4)	8780 (8330–9240)	2·88 (2·51–3·29)	2010 (1730–2300)	1·05 (0·869–1·26)	754 (612–915)	0·226 (0·179–0·278)	149 (117–189)	0·198 (0·157–0·25)	107 (84·8–133)	0·126 (0·102–0·155)	75·6 (60·8–92·9)
United Arab Emirates	12·6 (11·9–13·2)	943 (879–1010)	8·61 (8·14–9·08)	760 (705–820)	2·62 (2·27–2·99)	126 (105–152)	0·926 (0·757–1·13)	33·6 (25·5–43·7)	0·168 (0·123–0·216)	7·52 (4·85–10·7)	0·147 (0·117–0·183)	9·94 (7·32–13·2)	0·0793 (0·0645–0·0974)	5·58 (4·14–7·4)
Uruguay	13·1 (12·5–13·8)	624 (591–657)	9·11 (8·62–9·61)	409 (386–432)	2·73 (2·38–3·12)	146 (128–166)	0·818 (0·661–1)	46·6 (37·3–57·3)	0·164 (0·124–0·211)	8·82 (6·53–11·6)	0·136 (0·109–0·171)	6·17 (4·85–7·74)	0·154 (0·125–0·189)	7·28 (5·87–8·9)
Uzbekistan	18 (17·3–18·9)	4770 (4550–4990)	13·7 (13–14·3)	3840 (3640–4040)	2·85 (2·48–3·24)	617 (526–713)	0·999 (0·822–1·21)	181 (146–225)	0·203 (0·157–0·254)	42·9 (32·5–55·3)	0·19 (0·15–0·237)	53·3 (40·5–68·4)	0·128 (0·104–0·158)	33 (25·6–42)
Vanuatu	21·6 (20·9–22·4)	47·5 (45·7–49·4)	16·5 (15·8–17·1)	37·2 (35·6–38·8)	3·24 (2·83–3·65)	6·34 (5·51–7·21)	1·11 (0·928–1·33)	2·03 (1·68–2·45)	0·292 (0·233–0·36)	0·631 (0·491–0·806)	0·29 (0·229–0·366)	0·783 (0·59–1·01)	0·216 (0·173–0·268)	0·534 (0·405–0·681)
Venezuela	21·9 (20·8–23·1)	6510 (6170–6860)	17·4 (16·3–18·4)	5210 (4900–5540)	3·08 (2·66–3·53)	884 (762–1020)	1·01 (0·824–1·24)	280 (229–344)	0·185 (0·143–0·233)	51 (39·6–64·4)	0·197 (0·156–0·247)	55·4 (43·4–69·2)	0·0849 (0·0684–0·104)	24 (19·3–29·5)
Vietnam	21·4 (20·7–22·2)	21500 (20800–22300)	16·6 (15·9–17·2)	17100 (16400–17800)	3·11 (2·7–3·5)	2850 (2450–3240)	1·03 (0·865–1·25)	895 (744–1090)	0·23 (0·181–0·285)	205 (159–256)	0·272 (0·215–0·342)	253 (197–319)	0·185 (0·149–0·229)	175 (140–217)
Virgin Islands	21·7 (20·5–22·9)	33·8 (31·8–36)	17·6 (16·5–18·6)	26·9 (25·1–28·9)	2·75 (2·37–3·14)	4·72 (3·95–5·48)	0·951 (0·77–1·17)	1·6 (1·25–2·03)	0·186 (0·147–0·234)	0·29 (0·223–0·381)	0·174 (0·138–0·219)	0·235 (0·179–0·301)	0·0651 (0·0524–0·0802)	0·0985 (0·0774–0·124)
Yemen	13 (12·4–13·6)	2340 (2210–2460)	8·09 (7·63–8·55)	1580 (1500–1670)	3·14 (2·73–3·55)	481 (419–546)	1·18 (0·983–1·44)	159 (132–190)	0·252 (0·196–0·317)	40·9 (31·9–51·1)	0·188 (0·147–0·233)	42·8 (32·5–55·4)	0·139 (0·113–0·172)	30 (23·2–38·9)
Zambia	18·7 (18–19·5)	2060 (1960–2170)	13·8 (13·2–14·4)	1550 (1470–1620)	2·96 (2·57–3·33)	319 (269–373)	1·31 (1·11–1·56)	122 (98·6–148)	0·343 (0·28–0·418)	35·6 (27·5–45)	0·135 (0·106–0·169)	19·9 (14·4–26·5)	0·149 (0·121–0·182)	19·5 (14·7–25·2)
Zimbabwe	18·6 (17·9–19·4)	1850 (1760–1930)	14·5 (13·9–15·2)	1480 (1410–1550)	1·98 (1·71–2·26)	177 (151–206)	1·33 (1·12–1·55)	109 (90·4–130)	0·39 (0·314–0·472)	34·8 (27·5–43·5)	0·193 (0·152–0·241)	23·4 (17·7–30·4)	0·182 (0·149–0·222)	19·6 (15·1–24·8)
eSwatini	19·1 (18·4–19·9)	154 (147–161)	14·4 (13·7–15)	119 (113–124)	2·52 (2·18–2·86)	18·7 (15·9–21·7)	1·37 (1·15–1·62)	9·31 (7·66–11·2)	0·426 (0·339–0·523)	3·14 (2·48–3·93)	0·225 (0·177–0·283)	2·18 (1·65–2·8)	0·194 (0·158–0·237)	1·67 (1·29–2·12)
